# *CA9* Silencing Promotes Mitochondrial Biogenesis, Increases Putrescine Toxicity and Decreases Cell Motility to Suppress ccRCC Progression

**DOI:** 10.3390/ijms21165939

**Published:** 2020-08-18

**Authors:** Jiatong Xu, Songbiao Zhu, Lina Xu, Xiaohui Liu, Wenxi Ding, Qingtao Wang, Yuling Chen, Haiteng Deng

**Affiliations:** 1 MOE Key Laboratory of Bioinformatics, Center for Synthetic and Systematic Biology, School of Life Sciences, Tsinghua University, Beijing 100084, China; xjt15@mails.tsinghua.edu.cn (J.X.); songbiao_zhu@163.com (S.Z.); xulina@mail.tsinghua.edu.cn (L.X.); xiaohuiliu@biomed.tsinghua.edu.cn (X.L.); dwx18@mails.tsinghua.edu.cn (W.D.); 2 Beijing Chaoyang Hospital Affiliated to Capital Medical University, Beijing 100043, China; wqt36@163.com

**Keywords:** CA9, ccRCC, proteomics, metabolomics, surfaceomics, mitochondrial biogenesis, putrescine toxicity, motility

## Abstract

Carbonic anhydrase IX (CA9), a pH-regulating transmembrane protein, is highly expressed in solid tumors, and particularly in clear cell renal cell carcinoma (ccRCC). The catalytic mechanisms of CA9 are well defined, but its roles in mediating cell migration/invasion and survival in ccRCC remain to be determined. Here, we confirmed that the mRNA expression of *CA9* in ccRCC was significantly higher than that in para-carcinoma tissues from analysis of the datasets in The Cancer Genome Atlas. *CA9* knockdown upregulated oxidative phosphorylation-associated proteins and increased mitochondrial biogenesis, resulting in the reversal of the Warburg phenotype and the inhibition of cell growth. Our study revealed that *CA9* knockdown upregulated mitochondrial arginase 2 (ARG2), leading to the accumulation of putrescine, which suppressed ccRCC proliferation. Surfaceomics analysis revealed that *CA9* knockdown downregulated proteins associated with extracellular matrix (ECM)—receptor interaction and cell adhesion, resulting in decreased cell migration. *CA9* silencing also downregulated amino acid transporters, leading to reduced cellular amino acids. Collectively, our data show that *CA9* knockdown suppresses proliferation via metabolic reprogramming and reduced cell migration, reaffirming that CA9 is a potential therapeutic target for ccRCC treatment.

## 1. Introduction

Carbonic anhydrase IX (CA9) is a transmembrane carbonic anhydrase (CA), catalyzing the reversible hydration of carbon dioxide (H_2_O+CO_2_⇔H^+^+HCO_3_^−^) to maintain the intracellular pH homeostasis [1,2]. Extracellular bicarbonate ions produced by CA9 are imported into cytoplasm by bicarbonate transporters, such as anion exchangers (AEs) and Na+/bicarbonate cotransporters (NBCs), for the regulation of intracellular pH [3,4,5,6]. Previous studies demonstrated that CA9 formed a transport metabolon with AEs, NBCs, and monocarboxylate transporters (MCTs) to maintain pH homeostasis in tumor cells [7,8,9]. Excess extracellular protons produced by CA9 can cause extracellular acidosis. CA9 is expressed in several normal tissues, such as gut epithelium and pancreatic duct, while it is overexpressed in tumors such as breast, lung, kidney, colon/rectum, cervix uteri, oral cavity (head/neck), gallbladder, and liver carcinomas [2,10,11,12,13]. Intracellular pH and the acidic extracellular microenvironment regulate tumor proliferation, invasion, metastasis, and responses to chemotherapy and radiotherapy [14,15,16,17,18,19]. Extensive studies have proposed that CA9 is a potential diagnostic biomarker and a therapeutic target for tumors [5,20].

Clear cell renal cell carcinoma (ccRCC) is the most common form of renal cancer, accounting for 70–80% of all renal cell cancer cases [21,22]. *VHL* (von Hippel—Lindau disease gene) is ubiquitously inactivated by mutation or promoter hypermethylation in ccRCC [23,24,25], which results in the persistent stabilization and activation of hypoxia-inducible factor (HIF) [26,27]. ccRCC has the typical Warburg phenotype with enhanced glycolysis and deactivated tricarboxylic acid cycle (TCA) [28,29,30,31]. Our previous studies demonstrated that uncoupling between glycolysis and TCA switched mitochondrial function from ATP production to glutamine-dependent biosynthesis, suggesting that metabolic reprogramming occurred in ccRCC progression [32]. As one of the gene targets regulated by HIF, *CA9* is highly expressed, even under normoxia in most ccRCC [33]. Studies have shown that the high expression of CA9 promotes viability and growth of tumor cells in melanoma, breast, and colorectal cancers [34,35], and enhances tumor invasion and migration by promoting extracellular matrix degradation [36,37]. Furthermore, CA9 inhibition sensitizes colorectal carcinoma and renal cell carcinoma to irradiation [38,39], and induces ferroptosis in malignant mesothelioma [40]. Significant progress has been made in recent years for the characterization of CA9 as a potential diagnosis, prognosis, and therapeutic target. [41,42,43]. However, few studies have focused on the effects of CA9 on cellular metabolism in ccRCC.

Herein, we manipulated *CA9* expressions by knockdown and overexpression in ccRCC cells and systematically analyzed effects of CA9 on promoting cell survival and migration. We found that *CA9* silencing resulted in the accumulation of putrescine and increased mitochondrial biogenesis, leading to decreased cell proliferation. We carried out a quantitative surfaceomics analysis and found that *CA9* silencing downregulated amino acid transporters and proteins associated with cell motility.

## 2. Results

### 2.1. CA9 Knockdown Inhibits Cell Growth in ccRCC Cells

**Figure 1:**
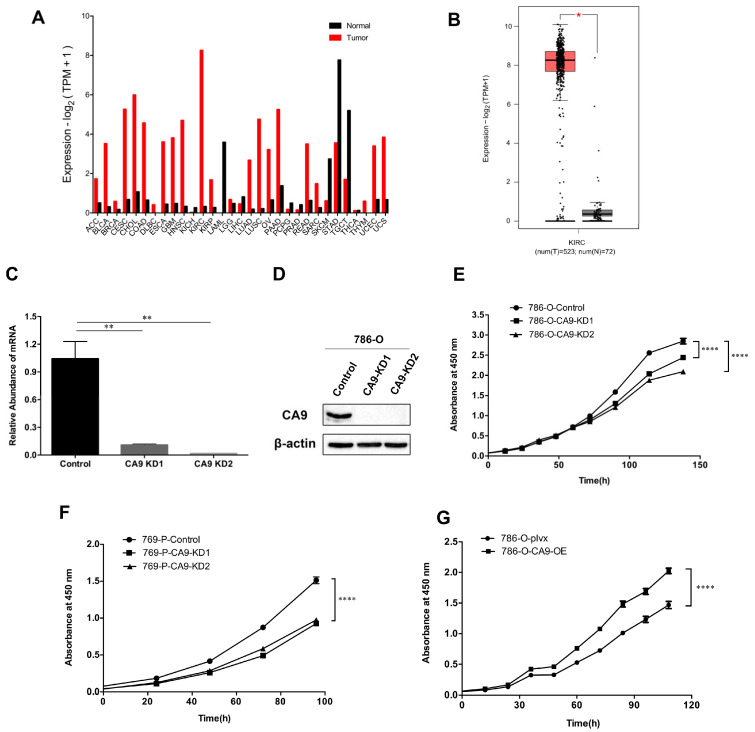
*CA9* knockdown inhibits cell growth in clear cell renal cell carcinoma (ccRCC) cells. (**A**) *CA9* expression profile across all tumor samples and paired normal tissues. The transcriptome datasets were obtained from The Cancer Genome Atlas (TCGA). Tumor abbreviations are listed in Table S1. (**B**) The mean mRNA level of *CA9* in ccRCC tissues (num(T) = 523) and normal tissues (num(N) = 72) from TCGA data. (**C**) mRNA expression of *CA9* decreased in 786-O-CA9-KD cells compared with control cells, measured by quantitative real-time PCR (qPCR). *ACTB* was used as a control. (**D**) Western blotting of CA9 revealed that the expression of CA9 was reduced in 786-O-CA9-KD cells. β-actin was used as a control. (**E**) Knockdown of *CA9* in 786-O cells inhibited cell growth compared with control cells. (**F**) Knockdown of *CA9* in 769-P cells inhibited cell growth. (**G**) Overexpression of *CA9* in 786-O cells promoted cell proliferation. Cell proliferation curves measured by the Cell Counting Kit-8 (CCK-8) assays. Significance was calculated by Student’s t-test. **** *p* < 0.0001, ** *p* < 0.01, * *p* < 0.05; *n* = 3, mean ± SEM.

To confirm that CA9 is overexpressed in tumor tissues compared with para-carcinoma tissues in kidney renal clear cell carcinoma (KIRC), we analyzed the transcriptome data of paired tumor samples and normal tissues in The Cancer Genome Atlas (TCGA) (Figure 1A). We found that *CA9* was significantly higher in KIRC tissues than in normal tissues (Figure 1B). To examine the effects of CA9 on ccRCC progression, we knocked down (KD) or overexpressed (OE) *CA9* in 786-O and 769-P to establish stable cell lines. Two different short hairpin RNAs (shRNAs) against *CA9* were used to establish stable *CA9* knockdown cell lines, designated CA9 KD1 and KD2, and cells expressing nontargeting shRNA were used as negative control. Cells were transduced with the lentivirus vector encoding human *CA9* to stably overexpress CA9, and were designated 786-O-CA9-OE, while the cells transduced with the lentivirus vector encoding the empty pLVX-IRES-ZsGreen1 cassette were used as the control, designated 786-O-plvx. The expression of CA9 in 786-O and 769-P cells was confirmed by quantitative real-time PCR (qPCR) and Western blotting, revealing that CA9 expression was reduced by more than 90% and 60% in 786-O-CA9-KD and 769-P-CA9-KD cells, respectively, compared with control cells, while it was increased more than 60-fold in 786-O-CA9-OE cells (Figure 1C,D and Figure S1). Cell Counting Kit-8 (CCK-8) assays showed that *CA9* knockdown inhibited cell growth (Figure 1E,F), while *CA9* overexpression promoted cell proliferation (Figure 1G). These results demonstrate that high CA9 expression promoted ccRCC cell proliferation, while low expression inhibited cell growth.

### 2.2. CA9 Knockdown Increases Mitochondrial Biogenesis and Reverses Warburg Metabolic Phenotypes

**Figure 2:**
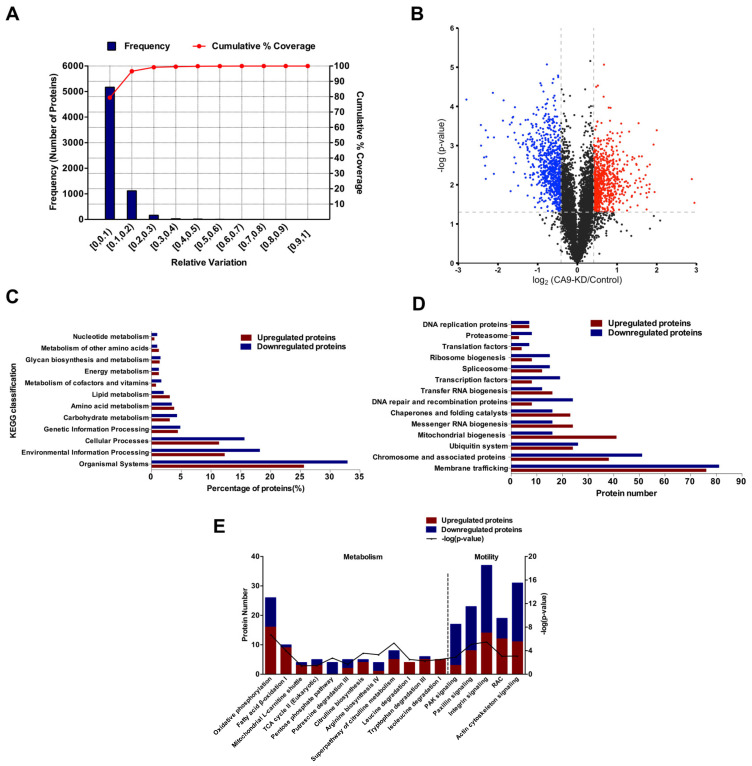
Functional classification of differentially expressed proteins (DEPs) in proteomics analysis of 786-O *CA9* knockdown cells compared with control cells. (**A**) Experimental variations of proteomics analysis between 786-O-CA9-KD cells and control cells. (**B**) A volcano plot of proteins based on *p*-values and ratios of protein expressions in 786-O-CA9-KD cells compared with control cells. Blue and red dots indicate the downregulated and upregulated proteins with significant difference (*p*-values < 0.05, ratios ≤ 0.75 or ≥1.33), respectively. (**C**) The upregulated and downregulated proteins after *CA9* knockdown in 786-O cells were classified by Kyoto Encyclopedia of Genes and Genomes (KEGG) BlastKOALA. (**D**) Numbers of upregulated and downregulated proteins participating in genetic information processing. Most mitochondrial biogenesis proteins were upregulated in *CA9* knockdown cells. (**E**) Representative canonical pathways enriched in DEPs after *CA9* knockdown in 786-O cells, analyzed by IPA (Ingenuity Pathway Analysis).

To elucidate CA9 function in the progression of ccRCC, quantitative proteomics analysis was carried out to characterize the proteome changes after *CA9* knockdown in 786-O cells. Most experiments were conducted using the CA9-KD2 cells in the present study. The percentage variations corresponding to 88% coverage were taken as the threshold cut-off [44]; thus, proteins with ratios ≥1.33 or ≤0.75 and *p*-values <0.05 were regarded as the upregulated or downregulated proteins, respectively, in our results (Figure 2A,B). A total of 6513 proteins were identified in biological triplicates including 748 downregulated proteins and 726 upregulated proteins in 786-O-CA9-KD cells compared with control cells (Tables S2 and S3). Differentially expressed proteins (DEPs) after *CA9* knockdown in 786-O cells were analyzed by Kyoto Encyclopedia of Genes and Genomes (KEGG) BlastKOALA [45]. The downregulated proteins were enriched in pathways of carbohydrate metabolism, cofactors and vitamins metabolism, and nucleotide metabolism (Figure 2C). The genetic information processing pathways enriched by the downregulated proteins were mainly focused on ribosome biogenesis, DNA repair and recombination, and chromosome and transcription factors. Interestingly, most mitochondrial proteins were upregulated in *CA9* knockdown cells (Figure 2D). Ingenuity Pathway Analysis (IPA) further showed that proteins related to oxidative phosphorylation (OXPHOS), fatty acid β-oxidation, and mitochondrial L-carnitine shuttle were upregulated, while proteins associated with the pentose phosphate pathway (PPP) were downregulated in *CA9* knockdown cells (Figure 2E). Furthermore, the arginine metabolism-related pathways and the pathways related to cell motility, including p21-activated kinase (PAK) signaling, paxillin signaling, integrin signaling, Ras-related C3 botulinum toxin substrate (RAC) and actin cytoskeleton signaling, were also enriched in DEPs (Figure 2E). These results indicate that CA9 expression regulated cell metabolism and cell motility.

**Figure 3:**
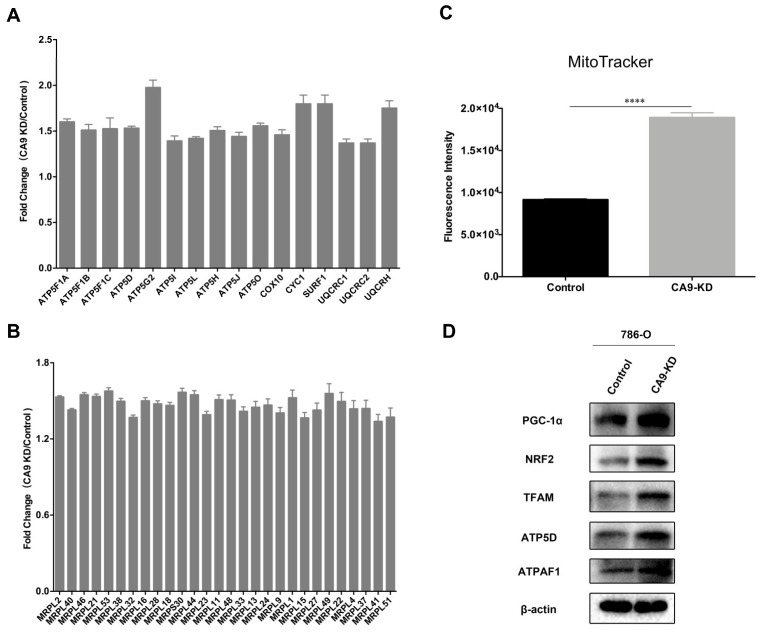
*CA9* knockdown increases mitochondrial biogenesis in 786-O cells. (**A**) Sixteen oxidative phosphorylation (OXPHOS)-related proteins and (**B**) 28 mitochondrial ribosomal proteins were upregulated in *CA9* knockdown cells (*n* = 3, mean ± SEM). (**C**) *CA9* knockdown increased MitoTracker staining intensity (**** *p* < 0.0001, *n* = 4, mean ± SEM). (**D**) Western blot images of peroxisome proliferator-activated receptor gamma coactivator 1-alpha (PGC-1α), nuclear factor erythroid 2-related factor 2 (NRF2), mitochondrial transcription factor 1 (TFAM), ATP synthase subunit delta (ATP5D) and ATP synthase mitochondrial F1 complex assembly factor 1 (ATPAF1) in control cells and CA9-KD cells. β-actin was used as a control. *CA9* knockdown enhanced protein expressions of key factors in mitochondrial biogenesis in 786-O cells.

As shown in Figure 3A,B, 16 OXPHOS-related proteins and 28 mitochondrial ribosomal proteins were upregulated in CA9-KD cells; thus, we inferred that mitochondrial biogenesis was upregulated after *CA9* knockdown. As predicted, *CA9* knockdown increased MitoTracker staining intensity and enhanced the expressions of key factors in mitochondrial biogenesis, including peroxisome proliferator-activated receptor gamma coactivator 1-alpha (PGC-1α), nuclear factor erythroid 2-related factor 2 (NRF2), and mitochondrial transcription factor 1 (TFAM) (Figure 3C,D and Figure S2A–C) in 786-O and 769-P cells [46,47]. Furthermore, mitochondrial DNA (mtDNA) content, which is proportional to the number of mitochondria [46,48], was also increased after *CA9* knockdown in 786-O and 769-P cells (Figure S2D). Reactive oxygen species (ROS) are byproducts of electron transport chain activity, and we reasoned that an increase in the mitochondrial biogenesis could enhance the cellular ROS level after *CA9* knockdown [49]. Indeed, the ROS level was higher in *CA9* knockdown cells detected by CellRox Deep Red Reagent (Figure S2E). Moreover, *CA9* knockdown resulted in downregulation of glycolytic enzymes such as hexokinase-2 (HK2), glucose-6-phosphate 1-dehydrogenase (G6PD), ATP-dependent 6-phosphofructokinase (PFKM), and phosphoglycerate mutase 1 (PGAM1). Consistently, levels of glucose-6-phosphate (G6P), lactate, Uridine-5′-diphosphate glucose (UDP-G), and 6-phosphpogluconate (6PG) were decreased after *CA9* knockdown (Figure S3). These results demonstrated that *CA9* knockdown increased mitochondrial biogenesis and reversed the Warburg metabolic phenotypes.

### 2.3. CA9 Knockdown Increases Putrescine Production to Inhibit Cell Growth

**Figure 4:**
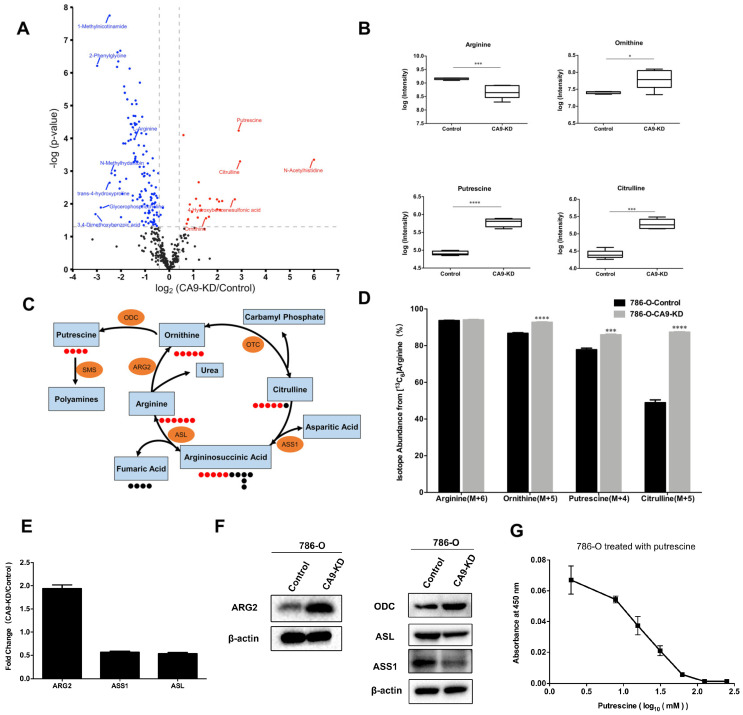
*CA9* knockdown increases putrescine production from arginine and inhibits cell growth. (**A**) A volcano plot of metabolites in 786-O-CA9-KD cells compared with control cells. The plot displays the fold changes of metabolite contents between CA9-KD cells and control cells against the *p*-values (−log10). Blue and red dots indicate the decreased and increased metabolites with significant difference (*p*-values < 0.05, fold changes ≤ 0.75 or ≥1.33), respectively. (**B**) Boxplots of metabolite intensity in putrescine synthesis pathway in CA9-KD cells and control cells (*n* = 5, mean ± SEM). (**C**) Schematic representation of putrescine biosynthesis from ^13^C_6_-arginine; red dots represent heavy carbon with ^13^C labeling while black dots represent light carbon (^12^C). The blue rectangles indicate metabolites and orange ovals indicate related enzymes. ODC, ornithine decarboxylase; ARG2, arginase 2; ASS1, argininosuccinate synthase 1; ASL, argininosuccinate lyase; SMS, spermine synthase; OTC, ornithine carbamoyltransferase. (**D**) Isotope relative abundance of M+5 ornithine, M+5 citrulline, and M+4 putrescine in 786-O-CA9-KD cells and control cells. Cells were supplemented with ^13^C_6_-arginine for 12 h prior to quantify metabolite levels via mass spectroscopy analysis (*n* = 3, mean ± SEM). (**E**) Expression ratios of enzymes involved in the putrescine synthesis between 786-O-CA9-KD cells and control cells (*n* = 3, mean ± SEM). (**F**) ARG2 and ODC were upregulated after *CA9* knockdown, while ASS1 and ASL were downregulated, confirmed by Western blotting. β-actin was used as a control. (**G**) Putrescine inhibited the growth of 786-O cells. The viability was assessed by the CCK-8 assay (*n* = 3, mean ± SEM). **** *p* < 0.0001, *** *p* < 0.001, * *p* < 0.05.

To confirm proteomics results suggesting that *CA9* silencing reprogramed cellular metabolism, we performed metabolomics analysis in five biological replicates to investigate the effects of *CA9* knockdown on cellular metabolism in 786-O cells. Metabolomics analysis showed that *CA9* knockdown increased the cellular contents of 24 metabolites while it decreased 127 metabolites (Tables S4 and S5). Interestingly, we found that putrescine was increased seven-fold in *CA9* knockdown cells compared with control cells (Figure 4A). Putrescine is generated from ornithine decarboxylation catalyzed by ornithine decarboxylase (ODC) and ornithine production is dependent on arginine metabolism [50,51]. Indeed, the metabolomics results show that *CA9* knockdown decreased the abundance of arginine, whereas it increased the contents of putrescine, ornithine, and citrulline (Figure 4B).

Then, isotope tracing with ^13^C_6_-arginine was performed to determine the effects of *CA9* knockdown on putrescine production from arginine. Five carbons in arginine are converted to ornithine, and then four carbons are incorporated into putrescine during putrescine synthesis (Figure 4C). ^13^C_6_-arginine isotope tracing revealed that M+5 ornithine, M+5 citrulline, and M+4 putrescine increased after *CA9* knockdown, suggesting putrescine synthesis was enhanced (Figure 4D). Arginase 2 (ARG2), a mitochondrial enzyme catalyzing the conversion of arginine to ornithine, was significantly upregulated after *CA9* knockdown identified by quantitative proteomics data and Western blotting. ODC catalyzing ornithine decarboxylation was also upregulated, while argininosuccinate synthase 1 (ASS1) and argininosuccinate lyase (ASL) catalyzing the conversion of citrulline to arginine were downregulated after *CA9* knockdown (Figure 4E,F and Figure S4). The expression of the enzymes involved in the metabolism of arginine in 769-P cells is consistent with that in 786-O cells (Figure S4). These results demonstrated that putrescine synthesis from arginine was increased by *CA9* knockdown in ccRCC cells. Putrescine was used to treat 786-O cells to examine its effects on cell growth. The viability of 786-O cells after putrescine treatment for 24 h was detected by CCK-8 kit, and putrescine was found to induce cell death with an IC50 (the half maximal inhibitory concentration) of 16.6 mM (Figure 4G), which is consistent with previous research [52].

### 2.4. CA9 Silencing Downregulates Amino Acid Transporters and Proteins Associated with Cell Motility in ccRCC Cells

**Figure 5:**
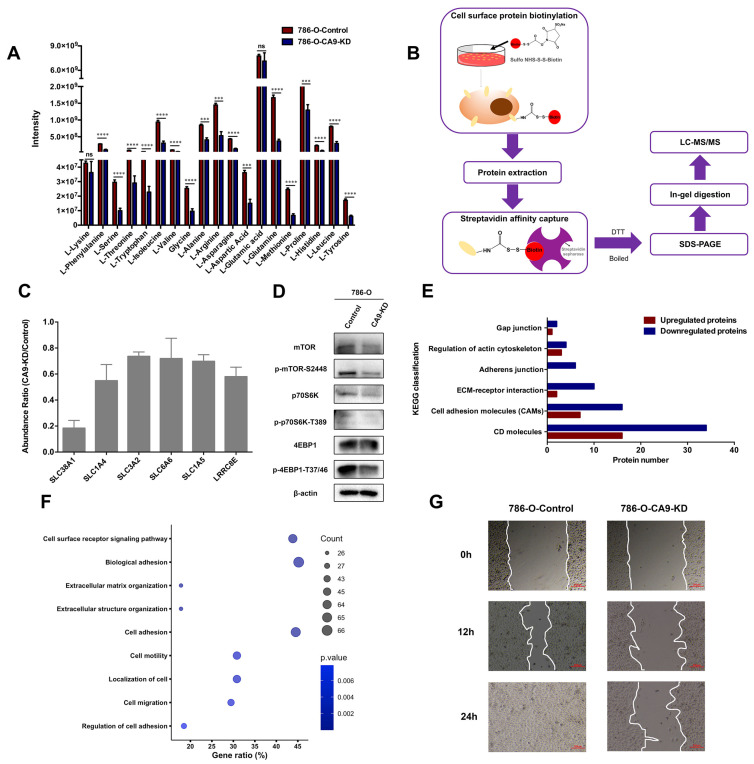
*CA9* silencing downregulated expressions of amino acid transporters and proteins associated with cell motility in ccRCC cells. (**A**) Abundance of amino acids in 786-O-CA9-KD cells and control cells. Most amino acids were reduced in *CA9* knockdown cells (**** *p* < 0.0001, *** *p* < 0.001, ns: no significance; *n* = 5, mean ± SEM). (**B**) Experimental design for the surfaceomics analysis. (**C**) Six amino acid transporters were downregulated in *CA9* knockdown cells (*n* = 3, mean ± SEM). (**D**) mammalian target of rapamycin (mTOR) pathway was inactivated in *CA9* knockdown cells, assessed by Western blotting of phosphor-mTOR (Ser2448), phospho-p70S6K (Thr389), and phospho-4EBP1 (Thr37/46). β-actin was used as a control. (**E**) Numbers of upregulated and downregulated surface proteins involved in cell motility. (**F**) The downregulated surface proteins in *CA9* knockdown cells analyzed by Gene Ontology with DAVID 6.8. (**G**) Cell migration was inhibited in 786-O-CA9-KD cells compared with that in control cells, assessed by the wound healing assay. Cells were imaged at 0, 12, and 24 h after scratching. Scale bar: 200 μm.

Metabolomics analysis also showed that most amino acids were reduced in *CA9* knockdown cells (Figure 5A). To explore whether *CA9* silencing downregulates amino acid transporters, we performed a surfaceomics analysis to investigate changes in membrane proteins between 786-O-CA9-KD cells and control cells. Briefly, surface proteins were biotinylated by sulfo-NHS-SS-Biotin and enriched by streptavidin conjugated sepharose, followed by in-gel digestion and liquid chromatography–tandem mass spectrometry (LC–MS/MS) analysis (Figure 5B). A total of 239 differentially expressed membrane proteins were identified, including 146 downregulated proteins and 93 upregulated proteins (Tables S6 and S7). We found that six amino acid transporters were downregulated in *CA9* knockdown cells, including sodium-coupled neutral amino acid transporter 1 (SLC38A1), neutral amino acid transporter A (SLC1A4), 4F2 cell-surface antigen heavy chain (SLC3A2), sodium- and chloride-dependent taurine transporter (SLC6A6), volume-regulated anion channel subunit LRRC8E (LRRC8E), and neutral amino acid transporter B(0) (SLC1A5) (Figure 5C). These results indicate that the intake of amino acids was decreased in *CA9* knockdown cells. In addition, the phosphorylation level of mammalian target of rapamycin (mTOR)-S2448 was decreased and the mTOR pathway was inactivated in 786-O-CA9-KD cells, detected by Western blotting (Figure 5D). Taking these results together, *CA9* knockdown decreased cellular amino acid levels and protein synthesis, which contributed to suppressed cell proliferation in 786-O cells.

Furthermore, KEGG BlastKOALA analysis showed that the expression of most surface proteins involved in extracellular matrix (ECM)–receptor interaction and cell adhesion was decreased in *CA9* knockdown cells, suggesting that *CA9* silencing decreased cell migration (Figure 5E). The differentially expressed membrane proteins were also analyzed by Gene Ontology with DAVID 6.8 [53]. Consistent with the KEGG analysis, the downregulated surface proteins were found to be mainly involved in cell motility, cell migration, cell adhesion, and extracellular matrix organization (Figure 5F). These results were also consistent with proteomics showing that pathways associated with cell motility, including PAK signaling, paxillin signaling, integrin signaling, RAC and actin cytoskeleton signaling, were enriched in the DEPs between 786-O-CA9-KD cells and control cells. To confirm the surfaceomics results, we measured the migration rates of *CA9* knockdown cells by a wound healing assay, and found that *CA9* silencing significantly inhibited the migration of 786-O and 769-P cells (Figure 5G and Figure S5).

## 3. Discussion

ccRCC displays the typical Warburg phenotype with enhanced glycolysis and decreased oxidative phosphorylation. Our previous studies demonstrated that proteostasis in ccRCC is highly correlated to this metabolic phenotype. For example, SIRT3 (NAD-dependent protein deacetylase sirtuin-3, mitochondrial) expression is lower in cancer tissues compared to the pericarcinous tissues, and SIRT3 overexpression enhances mitochondrial functions while it inhibits cell growth [54]. As the major chaperonin for mitochondrial homeostasis, *HSP60* (60 kDa heat shock protein, mitochondrial) knockdown switches the mitochondrial function from ATP production to biosynthesis to facilitate cell proliferation in ccRCC cells [32].

In contrast, CA9 was highly expressed in ccRCC and we proposed that *CA9* silencing enhanced oxidative phosphorylation and decreased cell growth. This was confirmed by results presented herein in which *CA9* silencing increased mitochondrial biogenesis, reversed the Warburg phenotype, and inhibited cell growth, whereas *CA9* overexpression promoted cell proliferation.

Moreover, CA9 is a carbonic anhydrase, catalyzing the reversible hydration of carbon dioxide to maintain the intracellular pH (pHi) homeostasis. We investigated the effects of *CA9* knockdown on pHi. We found that *CA9* knockdown resulted in a reduction in pHi in 786-O and 769-P cells (Figure S6), consistent with previous research [55]. It has been shown that CA9 enhances tumor invasion and migration by promoting extracellular matrix degradation through a pH-dependent mechanism [37]. In our study, we focused on the effects of CA9 on cellular metabolism in ccRCC. It has been found that an acidic pHi inhibits activities of glycolytic enzymes such as lactate dehydrogenase and phosphofructokinase 1 [56,57,58]. Although the effects of pHi on glycolytic enzymes need to be further investigated, our results suggest that metabolic changes we observed may be linked to a reduction of intracellular pH.

We carried out multi-omics analysis and revealed the global effects of *CA9* silencing on cellular processes. We found that *CA9* silencing activated oxidative phosphorylation, fatty acid oxidation, and mitochondrial biogenesis, as confirmed by fluorescent MitoTracker analysis. Consequently, ARG2 was upregulated as a mitochondrial protein, while ASS and ASL were downregulated, leading to putrescine accumulation which is toxic to ccRCC cells. This was validated by results showing that putrescine inhibited cell growth. However, the link between CA9 expression and ARG2 upregulation needs to be further explored. Putrescine, spermidine, and spermine are polyamines, which are vital to many cellular processes. ODC, catalyzing the decarboxylation of ornithine, is the first rate-limiting enzyme in polyamine biosynthesis and produces putrescine, which is a precursor of spermidine and spermine [50]. Polyamines can bind anionic complexes such as DNA, RNA, and proteins at physiological pH to modulate protein synthesis, gene expression, protection from oxidative damage, and maintenance of the structure of cellular macromolecules [59]. Polyamines play complex roles in pathological and physiological processes, such as angiogenesis and aging [59,60]. Increased polyamine can promote pancreatic ductal adenocarcinoma and colon cancer cell proliferation [61,62,63]. Conversely, excess polyamines and their by-products, such as aldehydes and ROS, are toxic to cells. Polyamines contribute to uremic toxins and liver damage [64,65]. Ochock et al., found that increased polyamines were toxic to ccRCC, and speculated that naturally occurring tumor hypoxia and constitutive HIF activity might prefer ccRCC cells with lower endogenous polyamine synthesis [52]. Thus, ccRCC cells are sensitive to putrescine accumulation. Our work revealed that *CA9* knockdown increased arginine-dependent putrescine production to inhibit cell growth. Furthermore, the polycationic nature of putrescine may be changed after *CA9* knockdown due to a reduction in pHi. However, the molecular mechanisms underlying polyamine toxicity remain elusive and need to be further explored.

Surfaceomics showed that *CA9* silencing downregulated multiple amino acid transporters, resulting in the decreased cellular amino acids levels as confirmed by metabolomics analysis. It is worth mentioning that *CA9* silencing also downregulated solute carrier family 4 member 4 (SLC4A4), a bicarbonate cotransporter, indicating that bicarbonate ions may regulate the expressions of these surface proteins.

Clear cell renal cell carcinoma is radioresistant, which is a challenge for its clinical treatment. Previous research found that inhibition of CA9 increases sensitivity of renal cell carcinoma to ionizing radiation [39]. The combined treatment of CA9 inhibitor and radiotherapy is a potential treatment option for ccRCC. Based on the results presented in this study in which *CA9* knockdown downregulated programmed cell death 1 ligand 1 (PD-L1), we proposed that CA9-inhibition may enhance immunotherapy efficacy. Furthermore, CA9 inhibition increased mitochondrial biogenesis, which may enhance the efficacy of glutaminase inhibition for ccRCC treatment [32]. CA9 is a transmembrane protein and highly expressed in solid tumors. Zhu et al., found that CA9 aptamer-functionalized targeted nanobubbles can be applied to ultrasound molecular imaging of tumor cells, and can improve the accuracy of early diagnosis [66]. Minn et al., synthesized [^64^Cu] XYIMSR-06, a dual-motif CA9 ligand, and found it can image ccRCC by positron emission tomography [67]. Therefore, molecular imaging reagents targeting CA9 can improve diagnostic accuracy.

## 4. Materials and Methods

### 4.1. Cell Culture

Human clear cell renal cell carcinoma cell lines 786-O and 769-P, and human kidney cell line 293T were purchased from the Cell Bank of the Chinese Academy of Sciences (Shanghai, China). 786-O and 769-P cells were cultured in 1640 medium (Wisent, Nanjing, China). 293T cells were cultured in Dulbecco’s Modified Eagle Medium (DMEM) medium (Wisent, Nanjing, China). The culture medium was supplemented with 1% penicillin/streptomycin (Wisent, Nanjing, China) and 10% fetal bovine serum (FBS) (Wisent, Nanjing, China). Cells were cultured in a 37 °C humidified incubator containing 5% CO_2_.

### 4.2. Establishment of Stable CA9-Knockdown and CA9-Overexpression Cell Lines

Two small hairpin RNA sequences of shRNA1 and shRNA2 were chosen for targeting *CA9*. A scrambled non-silencing shRNA without homologous sequences in the human genome was used as a negative control. The oligonucleotides were annealed and inserted into plasmid pLL3.7 to generate pLL3.7-CA9 vectors and a control vector. The vectors were transient transfected into 293T cells along with packing plasmids for lentivirus production using Lipofectamine™ 3000 (Invitrogen, Carlsbad, CA, USA) according to the manufacturer’s instructions. Lentivirus particles were harvested after 60 h and then transfected 786-O and 769-P cells with polybrene when cells reached 60% confluence. A single GFP (green fluorescent protein) -positive cell was sorted by a flow cytometer into a single well of a 96-well plate to generate the monoclonal stable cell line. The clone with intense and uniform GFP expression was selected and confirmed the efficiency of *CA9* knockdown by Western blotting. For the overexpression of *CA9*, human *CA9* cDNA was amplified from 786-O cells and cloned into the plasmid pLVX-IRES-ZsGreen1 to create the pLVX-CA9-IRES-ZsGreen1 vector. The blank plasmid pLVX-IRES-ZsGreen1 was negative control (plvx). The transfection and cell line sorting procedures were same as the construction of knockdown cell lines. Detailed primer sequences are listed in Table S8.

### 4.3. Western Blot Analysis

Cells were lysed in RIPA (radio-immunoprecipitation assay) lysis buffer (Solarbio, Beijing, China) supplemented with Protease Inhibitor Cocktail (MERCK, Darmstadt, Germany) and 1 mM PMSF (phenylmethylsulfonyl fluoride) on ice for 30 min. Then cells were sonicated for 2 min and centrifugated with a collection of the supernatant. Protein concentrations were determined using the BCA (bicinchoninic acid) protein assay kit (Solarbio, Beijing, China). Equal proteins were separated by sodium dodecyl sulfate polyacrylamide gel electrophoresis (SDS-PAGE) and transferred onto PVDF (polyvinylidene fluoride) membranes (Millipore, Billerica, MA, USA). Western blot analysis followed a standard procedure. Anti-CA9 antibody was purchased from Santa Cruz Biotechnology (Pasa Robles, CA, USA). Anti-β-actin, anti-TFAM, and anti-ODC antibodies were purchased from Abclonal (Wuhan, China). Anti-PGC-1α, anti-NRF2, anti-ASS1, and anti-ASL antibodies were purchased from Abcam (Cambridge, UK). Anti-ATP5D and anti-ATPAF1 antibodies were purchased from Proteintech (Chicago, IL, USA). Anti-ARG2, anti-mTOR, anti-phosphor-mTOR (Ser2448), anti-4EBP1, and anti-phosphor-4EBP1 (Thr37/46) antibodies were purchased from Cell Signaling Technology (Danvers, MA, USA). Anti-p70S6K and anti-phosphor-p70S6K (Thr389) antibodies were purchased from Sigma (St. Louis, MO, USA). Anti-mouse and anti-rabbit secondary antibodies were purchased from Cell Signaling Technology (Danvers, MA, USA).

### 4.4. Quantitative Real-Time PCR (qPCR)

Total RNA was isolated from cells with Trizol reagent (TIANGEN, Beijing, China). cDNA was synthesized from 2 μg total RNA using the reverse transcription kit (Promega, Madison, WI, USA) according to the manufacturer’s instructions. Total DNA was isolated from cells using DNAzol^®^ (MRC, Cincinnati, OH, USA) according to the manufacturer’s instructions. qPCR was performed with the Roche LightCycler 96 System (Roche, Basel, Switzerland) using SYBR green reaction mixture (CWBIO, Beijing, China) according to the manufacturer’s instructions. *ACTB* was used as an internal control for mRNA quantification. mtDNA content was measured by the relative abundance of human cytochrome oxidase 1 (human *mtCO1*) and *β-globin* was used as an internal control [48]. Primers were acquired from the Primer-Blast [68] and are listed in Table S8.

### 4.5. Cell Proliferation Assay with Cell Counting Kit-8 (CCK-8)

Cells were seeded in 96-well plates with 2000 cells per well. Cell proliferation rate was detected with the CCK-8 (Dojindo, Kumamoto, Japan) according to the manufacturer’s instructions. CCK-8 reagent was added into cells and cells were incubated in a cell incubator with 5% CO_2_ for 1.5 h at 37 °C. Then, absorbance at 450 nm was measured by a microplate reader (Bio-Rad, Hercules, CA, USA). For detecting survival rates of 786-O cells treated with putrescine, cells were seeded into 96-well plates with 3000 cells per well. Three wells were included in each group. After seeding for 5 h, cells were treated with putrescine (0, 1.95, 7.81, 15.63, 31.25, 62.5, 125, and 250 mM) for 24 h. Then CCK-8 reagent was added into treated cells, followed by incubation for 1.5 h, then absorbance at 450 nm was measured.

### 4.6. Quantitative Proteomics Analysis

Proteins were extracted from cells with 8 M urea in phosphate buffer saline (PBS) (Wisent, Nanjing, China), 1X protease inhibitor cocktail. Then, cells were sonicated for 2 min and centrifugated with a collection of the supernatant. Protein concentrations were measured using the BCA protein assay kit. In-solution digestion was performed. A total of 100 μg of protein extracted from each cell line was reduced with 5 mM dithiothreitol (DTT) at room temperature and alkylated with 12.5 mM iodoacetamide (IAM) in the dark at room temperature. Then, proteins were diluted to 1.5 M urea with PBS and digested with trypsin (Promega, Madison, WI, USA) overnight at 37 °C. The tryptic peptides were desalted using Sep-Pak desalting columns (Waters, Milford, MA, USA) and then desalted peptides were labeled with tandem mass tags (TMT) 6-plex reagents (Thermo Fisher Scientific, Waltham, MA, USA). The combined TMT-labeled peptides were desalted by Sep-Pak columns and separated with a UPLC 3000 system (Thermo Fisher Scientific, Waltham, MA, USA) with an XBridgeTM BEH300 C18 column (Waters, Milford, MA, USA) at a flow rate of 1 mL/min. The mobile phase A was H_2_O (pH 10) and the mobile phase B was 98% acetonitrile (pH 10). Peptides were separated with a gradient elution including an increase from 8% to 18% phase B for 30 min; 18% to 32% phase B for 22 min. Forty-eight fractions were dried by speedvac and recombined to 12 fractions. The fractions were dissolved in 20 μL of 0.1% (*v*/*v*) formic acid (FA) and analyzed by LC–MS/MS.

### 4.7. Liquid Chromatography-Tandem Mass Spectrometry (LC–MS/MS) Analysis

Labeled peptides were separated with a high performance liquid chromatography (HPLC) system (Thermo Fisher Scientific, Waltham, MA, USA), which was connected to a Orbitrap Fusion LUMOS Tribrid mass spectrometer (Thermo Fisher Scientific, Waltham, MA, USA). The mass spectrometer used the data-dependent acquisition mode with the Xcalibur 3.0 software (Thermo Fisher Scientific, Waltham, MA, USA). The parameters were as follows: a single full-scan mass spectrum was done in the Orbitrap (350–1550 *m*/*z*, 120,000 resolution) and the automatic gain control (AGC) target was 2 × 10^5^; acquisition settings for MS/MS spectra were 30,000 for resolution with the AGC target of 2 × 10^5^ and maximum injection time was 60 ms; the isolation window width was 0.7 Da; and the normalized collision energy for dissociation was 35%.

### 4.8. Peptide and Protein Identification

The UniProt human database (16 June 2017; 89,105 sequences) was used by the Sequest HT in Proteome Discoverer (PD) 2.1 software (Thermo Fisher Scientific, Waltham, MA, USA) to search the MS/MS spectra. The searching process used the following criteria: full tryptic specificity required, two missed cleavages tolerance, fixed modifications were carbamidomethylation and TMT 6-plex, variable modification was oxidation; 10 ppm tolerance for precursor ion mass for all MS acquired in the Orbitrap mass analyzer; and fragment ion mass tolerance was 20 mmu for all MS2 spectra acquired in the Orbitrap. The searched data were further processed with the percolator function in Proteome Discoverer to filter with 1% peptide false discovery rate (FDR). Relative protein quantification was carried out using PD 2.1 according to the intensities of reporter ions per peptide.

### 4.9. Detection of Mitochondria Biogenesis

Cells were stained with the fluorescent probe MitoTracker Deep Red FM (Invitrogen, Carlsbad, CA, USA) according to the manufacturer’s instructions. Briefly, cells were washed with PBS and stained in 0.1% BSA (*w*/*v*) containing 0.1 μM MitoTraker. After staining for 30 min at 37 °C, fluorescence of 10,000 cells was analyzed using a BD FACS (Fluorescence-Activated Cell Sorting System) Aria II Flow Cytometer (BD Biosciences, Franklin Lakes, NJ, USA). Relative quantification was determined by mean emission fluorescence intensity of 10,000 cells at 665 nm.

### 4.10. Detection of Cellular Reactive Oxygen Species

Cells were stained with the CellROX Deep Red Reagents (Invitrogen, Carlsbad, CA, USA) according to the manufacturer’s instructions. Briefly, cells were washed twice with PBS and stained with 5 μM CellROX Deep Red. After staining for 30 min at 37 °C, the fluorescence of 10,000 cells was analyzed using a BD Flow Cytometer (BD Biosciences, Franklin Lakes, NJ, USA). Relative quantification was determined by the mean emission fluorescence intensity of 10,000 cells at 665 nm.

### 4.11. Detection of Intracellular pH

The pHi was measured using the Intracellular pH Detection Kit (BestBio, Shanghai, China) according to manufacturer’s instructions. Briefly, cells were washed once with Hank’s Balanced Salt Solution (HBSS) (BestBio, Shanghai, China) containing 20 mM HEPES (Solarbio, Beijing, China) and stained with 5 uM BBcellProbe in HBSS containing 20 mM HEPES for 45 min at 37 °C. After washing with HBSS containing 20 mM HEPES twice, fluorescence intensity of cells at 640 nm was analyzed by a BD FACS Aria II Flow Cytometer (BD Biosciences, Franklin Lakes, NJ, USA). A pH calibration standard curve was obtained using the nigericin as described in a previous study [69]. The pHi values were obtained based on the standard curve.

### 4.12. Metabolomics Analysis

Metabolomics analysis was performed as previously described [70]. Briefly, the cells were washed twice with cold PBS and incubated with pre-chilled 80% methanol (−80 °C) for 1 h at −80 °C. Then, the cells were scraped in 80% methanol on dry ice and centrifuged for 5 min. The extracted metabolites in the supernatant were concentrated completely to dryness with a lyophilizer and the protein concentrations of centrifuged pellets were used for normalization. The dried metabolites were dissolved in 80% methanol and analyzed by LC–MS/MS. We used the TSQ Quantiva™ Triple Quadrupole Mass Spectrometer (Thermo Fisher Scientific, Waltham, MA, USA) with positive/negative ion switching for targeted metabolites quantitative analysis. The Q-Exactive Mass Spectrometer (Thermo Fisher Scientific, Waltham, MA, USA) was chosen for untargeted metabolites profiling. Metabolites were identified based on the retention time and the accurate mass measured with <5 ppm mass accuracy. Quantitative information of metabolites was analyzed by TraceFinder.

### 4.13. Isotope Tracing Metabolomics

We used arginine- and lysine-free RPMI1640 medium (Gibco, Thermo Fisher Scientific, Waltham, MA, USA) for metabolic labeling assay. The medium was supplemented with 10% dialyzed FBS (Biological Industries, Beit Haemek, Israel) and 1% penicillin/streptomycin. This medium also lacked lysine, which was added back to standard 1640 concentration (0.2 mM). For cell labeling, cells were cultured in 10 cm dishes with that medium supplemented with 1.1 mM ^13^C_6_-arginine (Cambridge Isotope Laboratories, Tewksbury, MA, USA) for 12 h. In parallel with this, an unlabeled culture that was supplemented with equal concentration of unlabeled arginine was used to identify unlabeled metabolites. Then, the metabolites were extracted for mass spectroscopy analysis as described in *Metabolomics analysis.*

### 4.14. Wound Healing Assay

Cells were cultured in 6-well plates until 95% confluence. A sterile 200 μL pipette tip was used to scratch the cells to generate a uniform wound in the well. The well was replenished with 1640 medium without FBS [71]. Closures of these wounds were imaged at 0, 12, and 24 h with a Nikon microscope (Olympus Corporation, Tokyo, Japan). The wound area was quantitatively evaluated with ImageJ 1.52k software (National Institutes of Health, Bethesda, MD, USA).

### 4.15. Surfaceomics Analysis

To isolate cell surface proteins, ~1 × 10^7^ cells were washed three times with PBS and biotinylated in the culture plate with 0.25 mg/mL of sulfo-NHS-SS-Biotin (APExBIO, Houston, TX, USA) in PBS for 10 min at room temperature. The residual sulfo-NHS-SS-Biotin reagent was quenched with 10 mM lysine. Cells were lysed in RIPA lysis buffer supplemented with Protease Inhibitor Cocktail and 1% SDS (*v*/*v*). Then, cells were sonicated for 2 min and centrifugated with collection of the supernatant. Protein concentrations were measured using the BCA protein assay kit. Samples were diluted with PBS to 0.1% SDS followed by the isolation of biotinylated proteins with streptavidin conjugated sepharose (GE Healthcare, Chicago, IL, USA) for 4 h at 4 °C according to the manufacturer’s instructions. The sepharose was washed five times and boiled with loading buffer (with DTT) (Solarbio, Beijing, China). The supernatant was separated on the SDS-PAGE followed by in-gel digestion and label-free analysis. Briefly, the gel was excised, reduced with 25 mM DTT at 55 °C and alkylated with 55 mM IAM in the dark at room temperature. In-gel digestion was carried out with trypsin overnight at 37 °C. The tryptic peptides were extracted twice using 50% acetonitrile aqueous solution with 0.1% FA. The extracts were dried by speedvac and dissolved in 20 μL of 0.1% FA for LC-MS/MS analysis. The MS/MS spectra were searched against the UniProt human database processed by the Sequest HT search engine in PD 2.2. The searching process used the following criteria: full tryptic specificity required, two missed cleavages tolerance, fixed modifications were carbamidomethylation and TMT 6-plex, variable modification was oxidation; 10 ppm tolerance for precursor ion mass for all MS acquired in the Orbitrap mass analyzer; and 20 mmu tolerance for fragment ion mass for all MS2 spectra acquired in the Orbitrap. The searched data were further filtered with the percolator function in the Proteome Discoverer software based on 1% FDR.

### 4.16. Statistical Methods

Data statistical analysis was carried out with GraphPad Prism 6.0 software (GraphPad, La Jolla, CA, USA). Significance was determined by the two-tailed Student’s *t*-test. *p*-values below 0.05 were considered significant.

## 5. Conclusions

In summary, the present study demonstrated that *CA9* knockdown increased mitochondrial biogenesis and putrescine production, and decreased the expression of surface proteins associated with amino acid transport and cell motility, leading to the reduced cell proliferation and migration in ccRCC. Our results shed light on CA9 functions in ccRCC progression, and reaffirm that CA9 is a potential therapeutic target for ccRCC.

## Supplementary Materials

The following are available online at https://www.mdpi.com/1422-0067/21/16/5939/s1

## Abbreviations

AE	anion exchanger
AGC	automatic gain control
ARG2	arginase 2
ASL	argininosuccinate lyase
ASS1	argininosuccinate synthase 1
ATP5D	ATP synthase subunit delta
ATPAF1	ATP synthase mitochondrial F1 complex assembly factor 1
BCA	bicinchoninic acid
CA9	carbonic anhydrase IX
CCK8	Cell Counting Kit-8
ccRCC	clear cell renal cell carcinoma
DEP	differentially expressed protein
DMEM	Dulbecco’s Modified Eagle Medium
DTT	dithiothreitol
ECM	extracellular matrix
FA	formic acid
FACS	Fluorescence-Activated Cell Sorting System
FBS	fetal bovine serum
FDR	false discovery rate
GFP	green fluorescent protein
G6P	glucose-6-phosphate
G6PD	glucose-6-phosphate 1-dehydrogenase
HBSS	Hank’s Balanced Salt Solution
HIF	hypoxia-inducible factor
HK2	hexokinase-2
HPLC	high performance liquid chromatography
HEPES	2-[4-(2-hydroxyethyl) piperazin-1-yl] ethanesulfonic acid
HSP60	60kDa heat shock protein, mitochondrial
IAM	iodoacetamide
IC50	the half maximal inhibitory concentration
IPA	Ingenuity Pathway Analysis
KD	knock down
KEGG	Kyoto Encyclopedia of Genes and Genomes
KIRC	kidney renal clear cell carcinoma
LC-MS/MS	liquid chromatography-tandem mass spectrometry
LRRC8E	volume-regulated anion channel subunit LRRC8E
MCT	monocarboxylate transporter
mtCO1	cytochrome c oxidase subunit 1
mtDNA	mitochondrial DNA
mTOR	mammalian target of rapamycin
NBC	Na+/bicarbonate cotransporter
NRF2	nuclear factor erythroid 2-related factor 2
ODC	ornithine decarboxylase
OE	overexpressed
OXPHOS	oxidative phosphorylation
PAK	p21-activated kinase
PBS	phosphate buffer saline
PD	Proteome Discoverer
PD-L1	programmed cell death 1 ligand 1
PFKM	ATP-dependent 6-phosphofructokinase
PGAM1	phosphoglycerate mutase 1
PGC1-α	peroxisome proliferator-activated receptor gamma coactivator 1-alpha
pHi	intracellular pH
PPP	pentose phosphate pathway
PMSF	phenylmethylsulfonyl fluoride
PVDF	polyvinylidene fluoride
qPCR	quantitative real-time PCR
RAC	Ras-related C3 botulinum toxin substrate
RIPA	radio-immunoprecipitation assay
ROS	reactive oxygen species
SDS-PAGE	sodium dodecyl sulfate polyacrylamide gel electrophoresis
shRNA	short hairpin RNA
SIRT3	NAD-dependent protein deacetylase sirtuin-3, mitochondrial
SLC1A4	neutral amino acid transporter A
SLC1A5	neutral amino acid transporter B(0)
SLC3A2	4F2 cell-surface antigen heavy chain
SLC4A4	solute carrier family 4 member 4
SLC6A6	sodium- and chloride-dependent taurine transporter
SLC38A1	sodium-coupled neutral amino acid transporter 1
TCA	tricarboxylic acid cycle
TCGA	The Cancer Genome Atlas
TFAM	mitochondrial transcription factor 1
TMT	tandem mass tags
UDPG	Uridine-5’-diphosphate glucose
6PG	6-phosphpogluconate
