# Supplementary material for: CA9 Silencing Promotes Mitochondrial Biogenesis, Increases Putrescine Toxicity and Decreases Cell Motility to Suppress ccRCC Progression
Source: Int J Mol Sci. 2020 Aug 18;21(16):5939. doi: 10.3390/ijms21165939 (PMC7460829; doi:10.3390/ijms21165939)

1. Original images of western blots of CA9 and β-actin in Figure 1D.

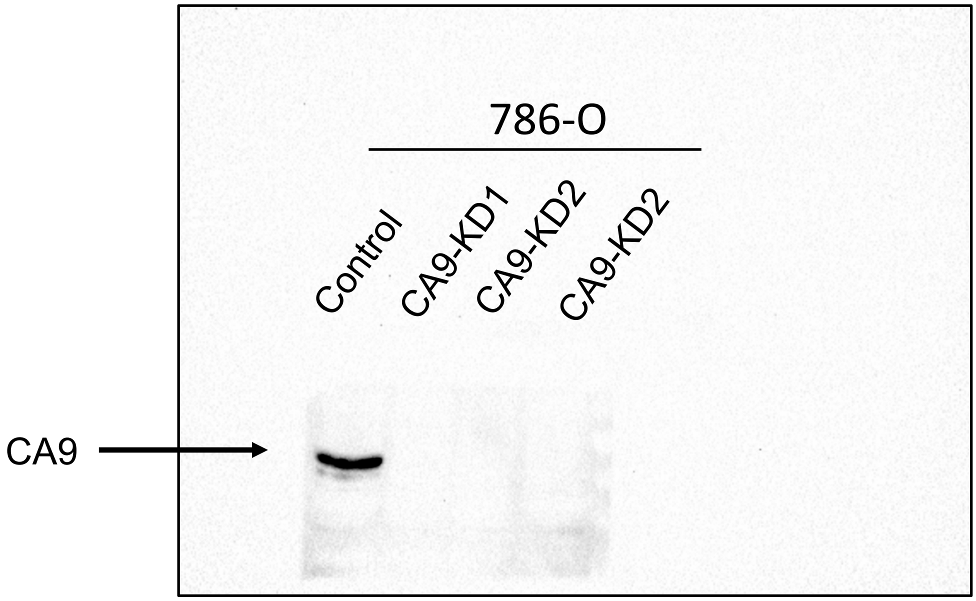

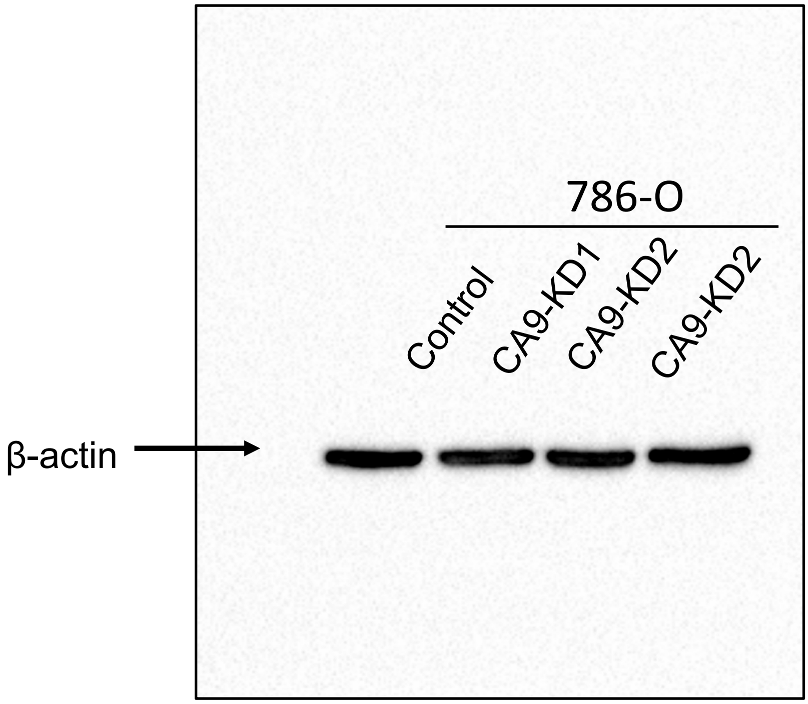

2. Original images of western blots of PGC-1α and ATP5D in Figure 3D.

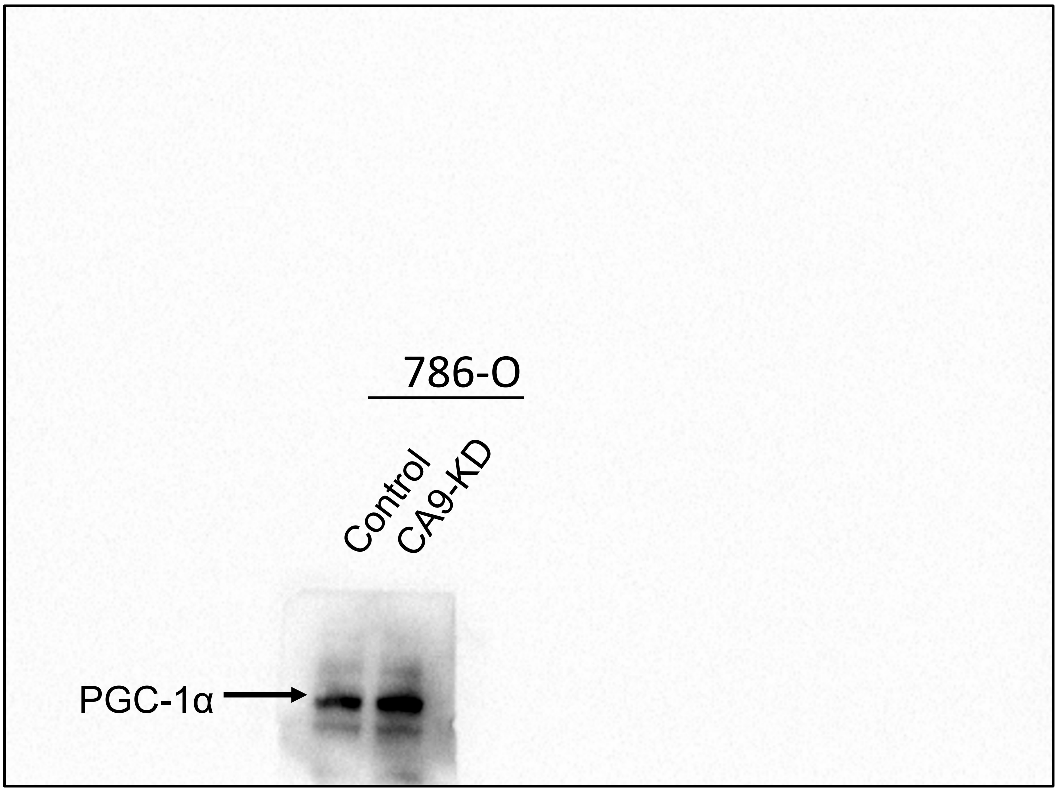

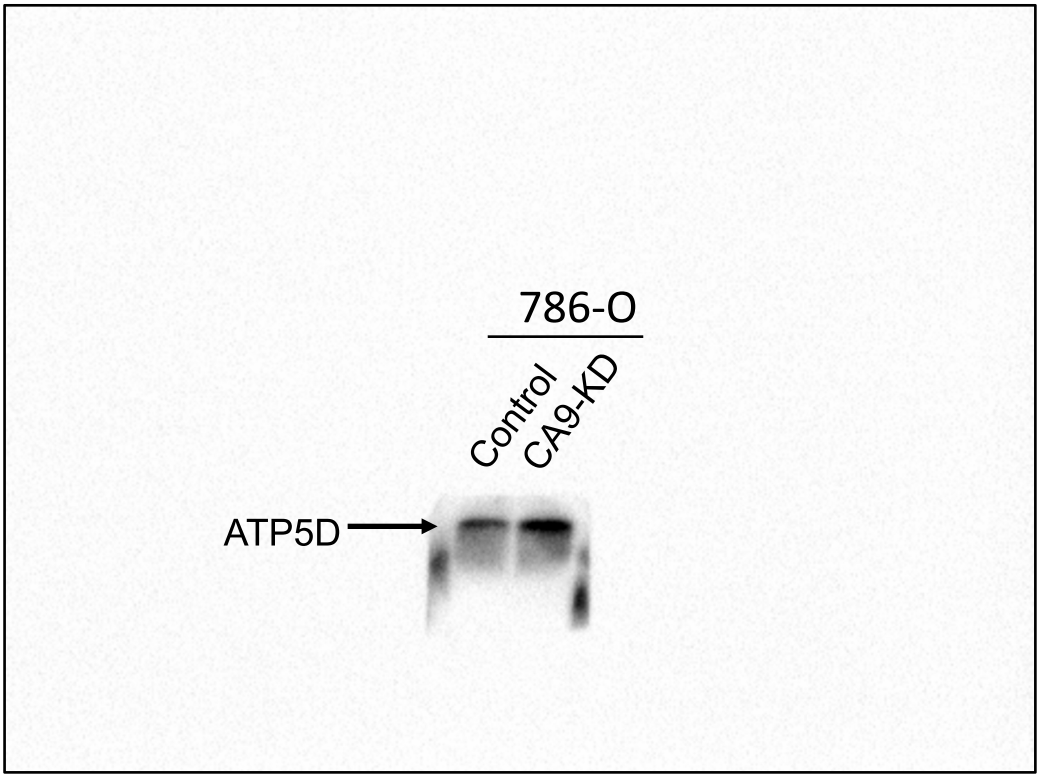

3. Original images of western blots of TFAM and ATPAF1 in Figure 3D.

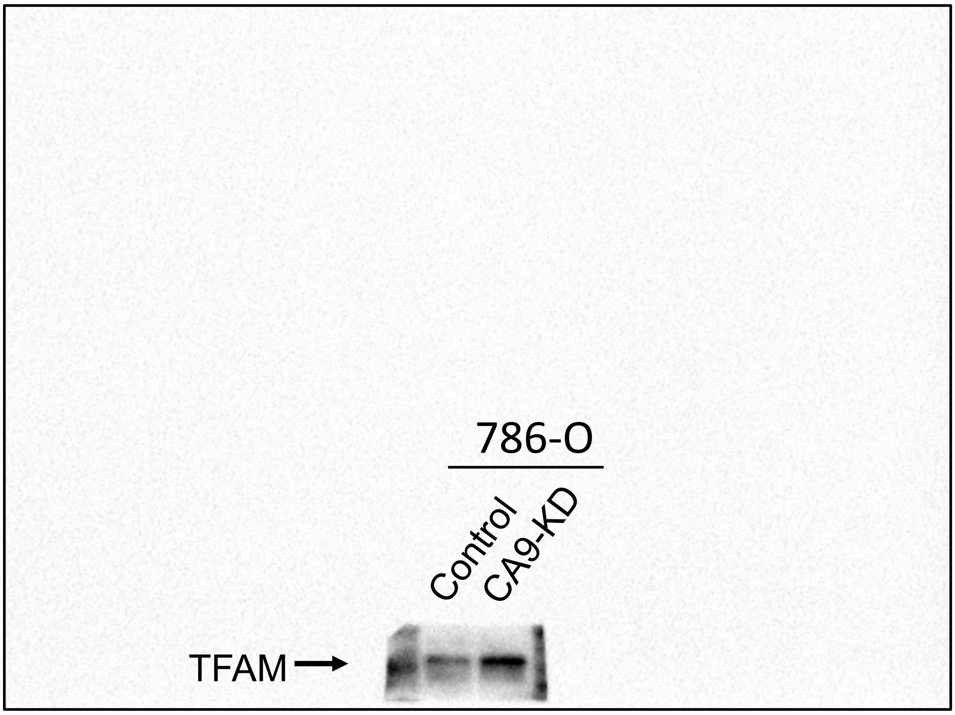

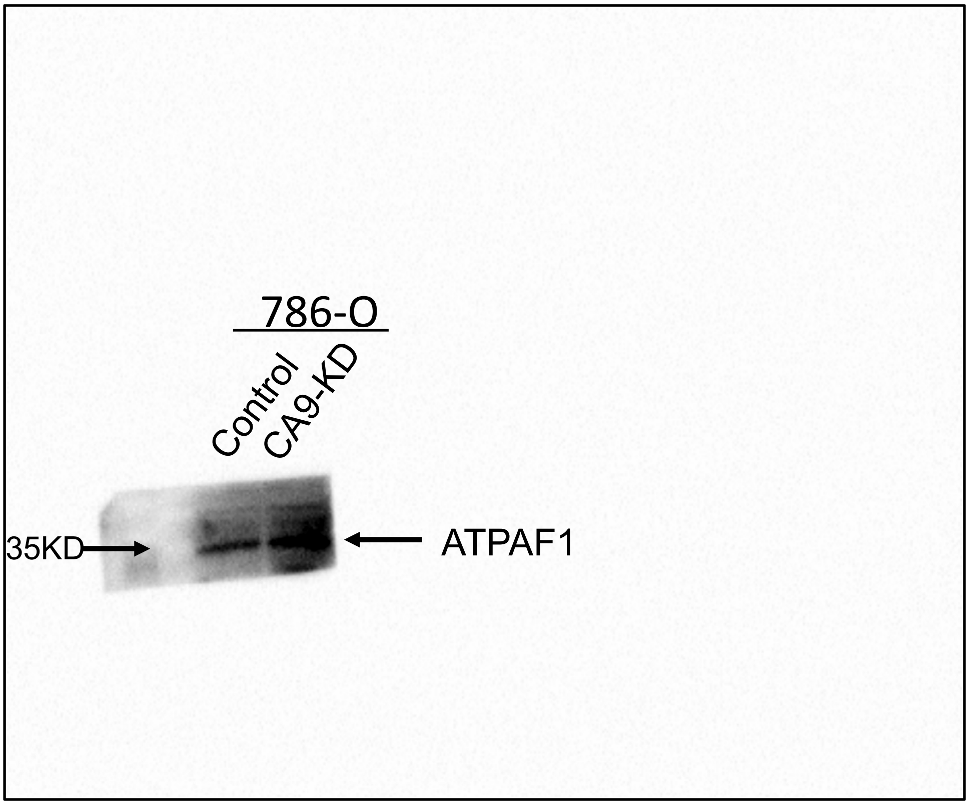

4. Original images of western blots of NRF2 and β-actin in Figure 3D.

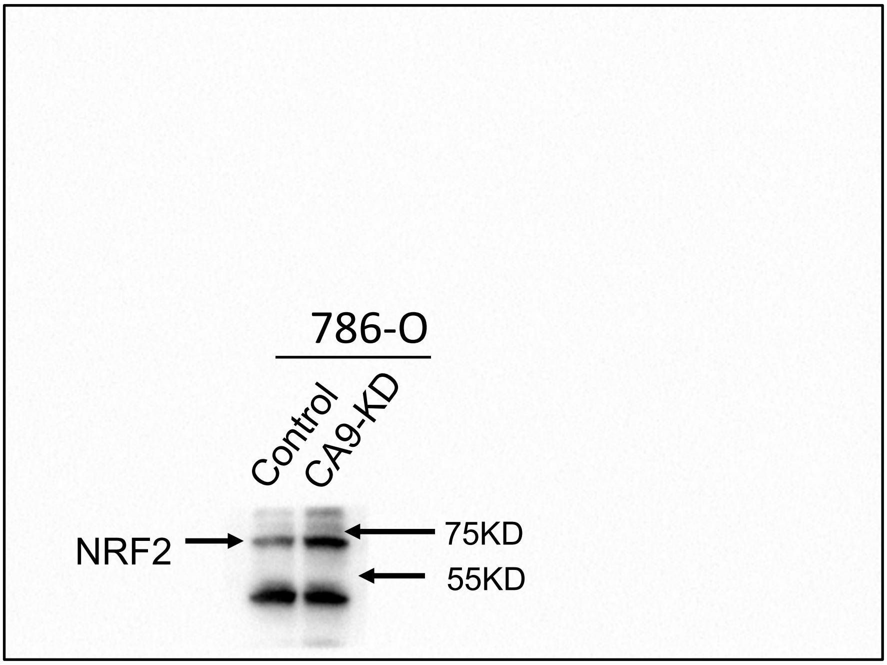

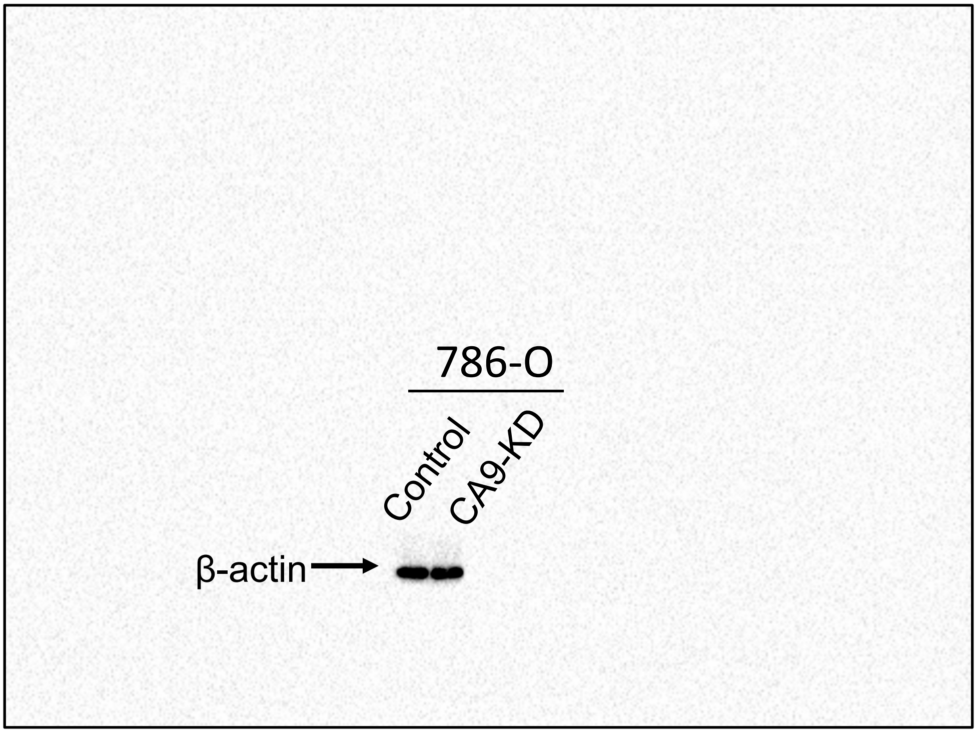

5. Original images of western blots of ARG2 and β-actin in Figure 4F.

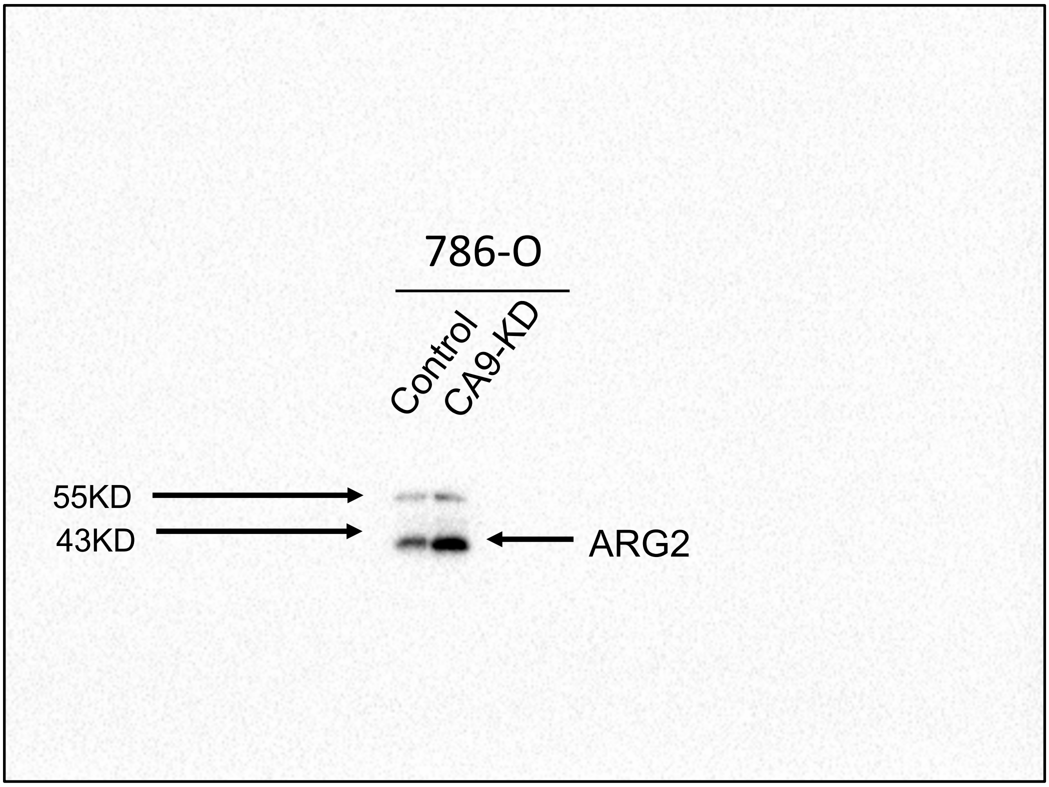

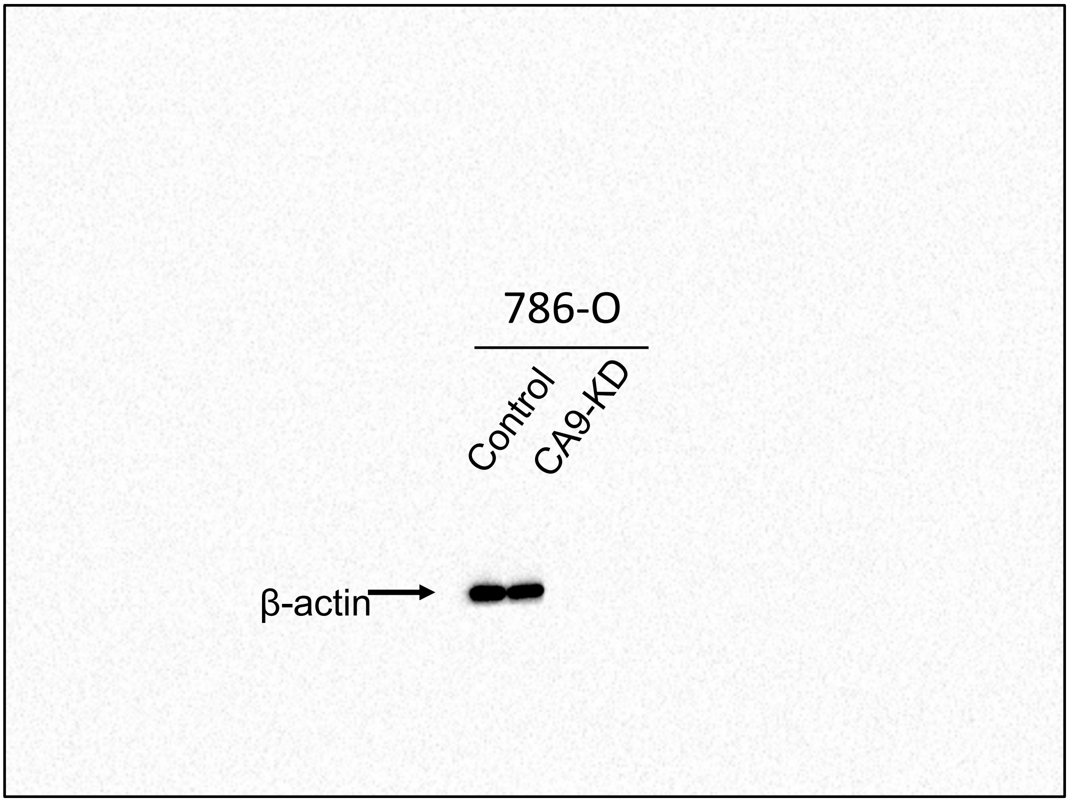

6. Original images of western blots of ASL, ASS1, ODC and β-actin in Figure 4F.

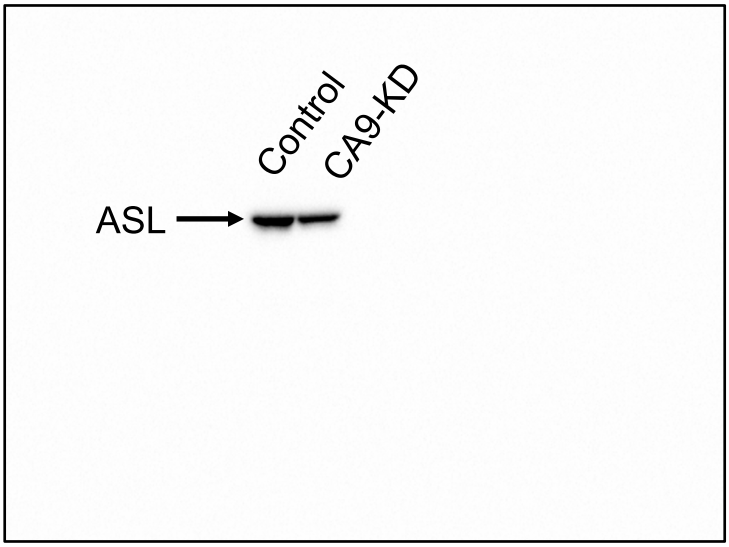

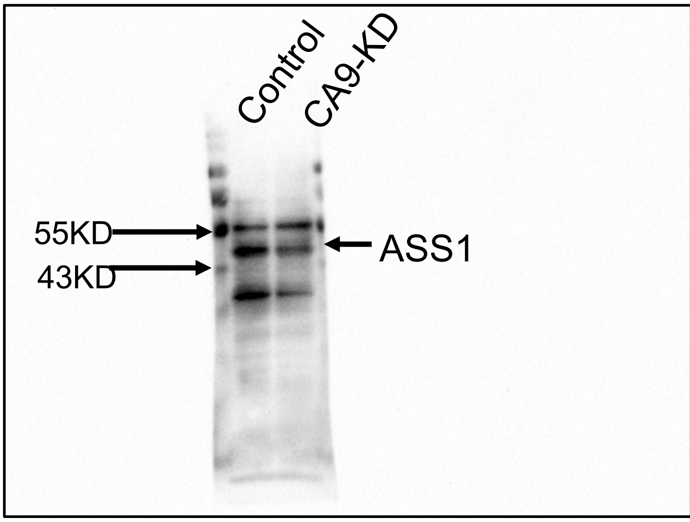

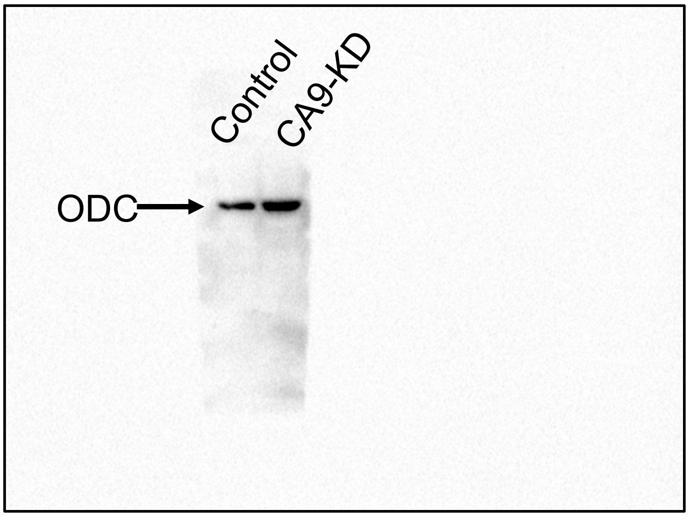

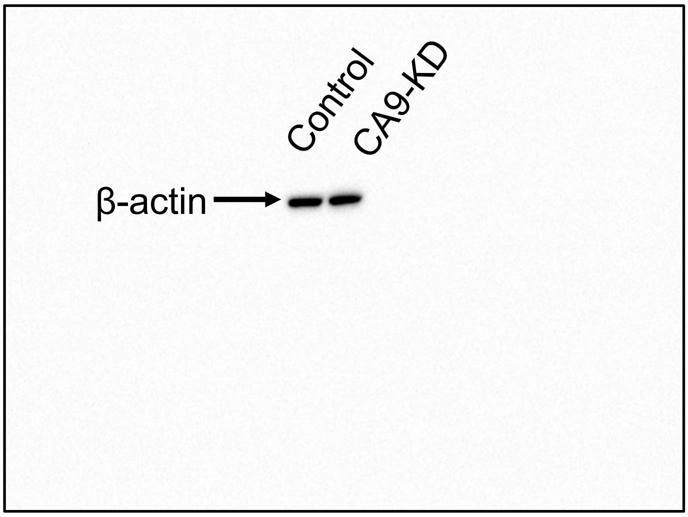

7. Original images of western blots of 4EBP1 and p-4EBP1-T37/46 in Figure 5D.

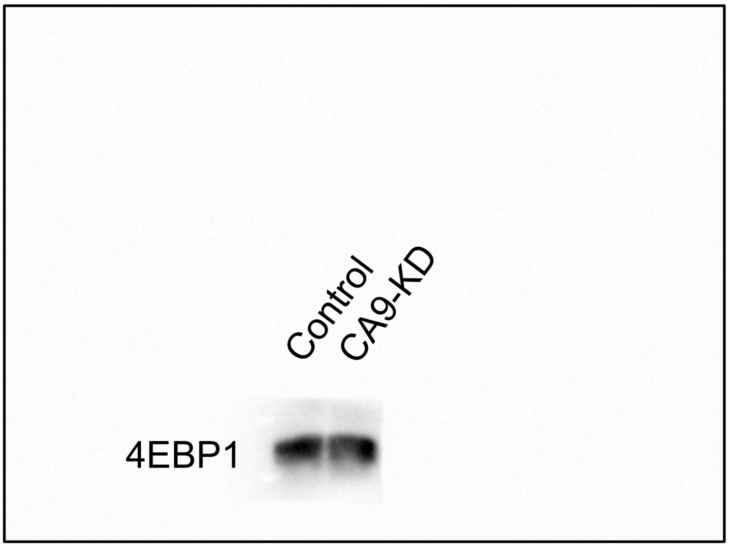

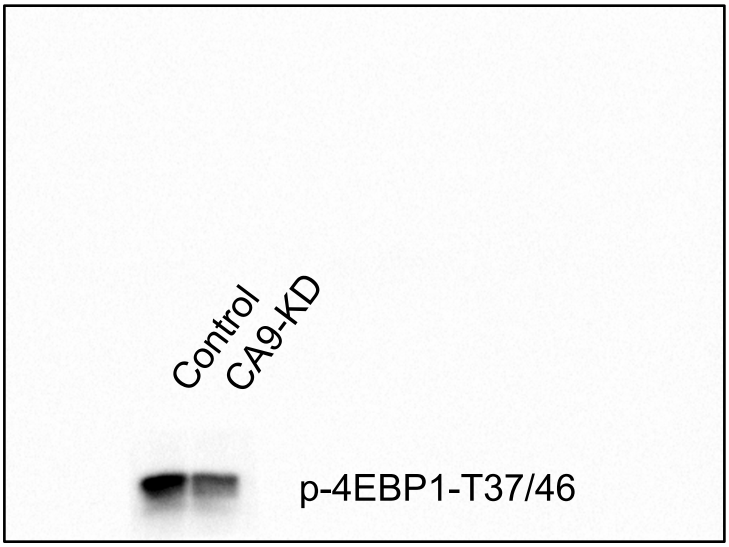

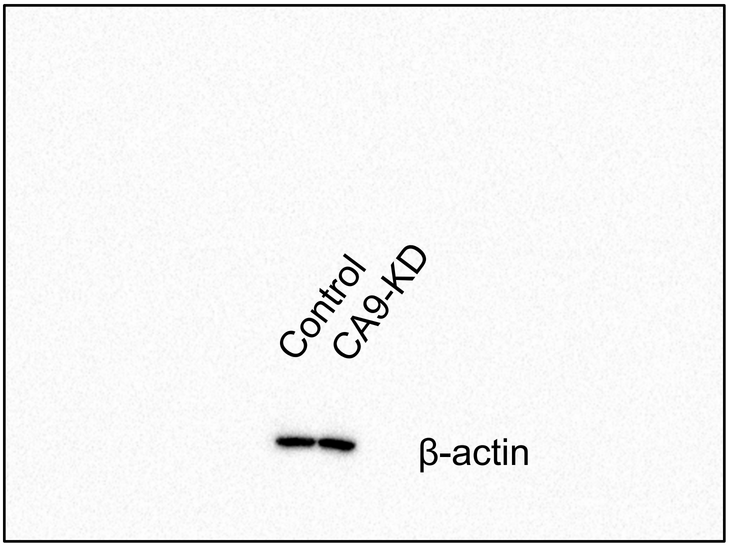

8. Original images of western blots of p70S6K and p-p70S6K-T389 in Figure 5D.

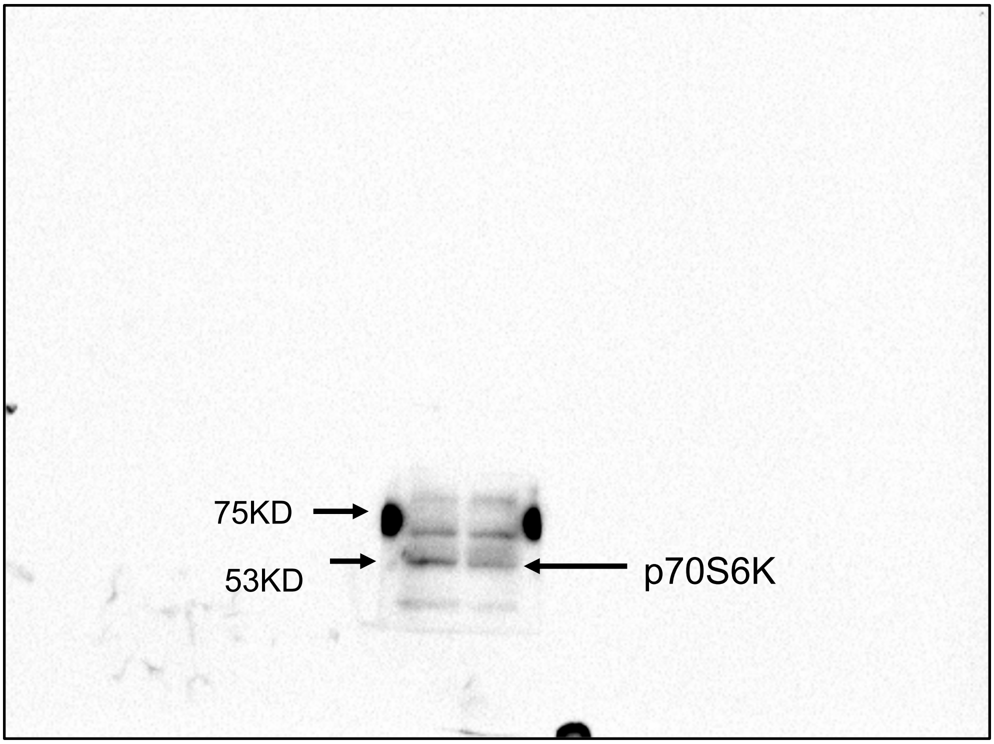

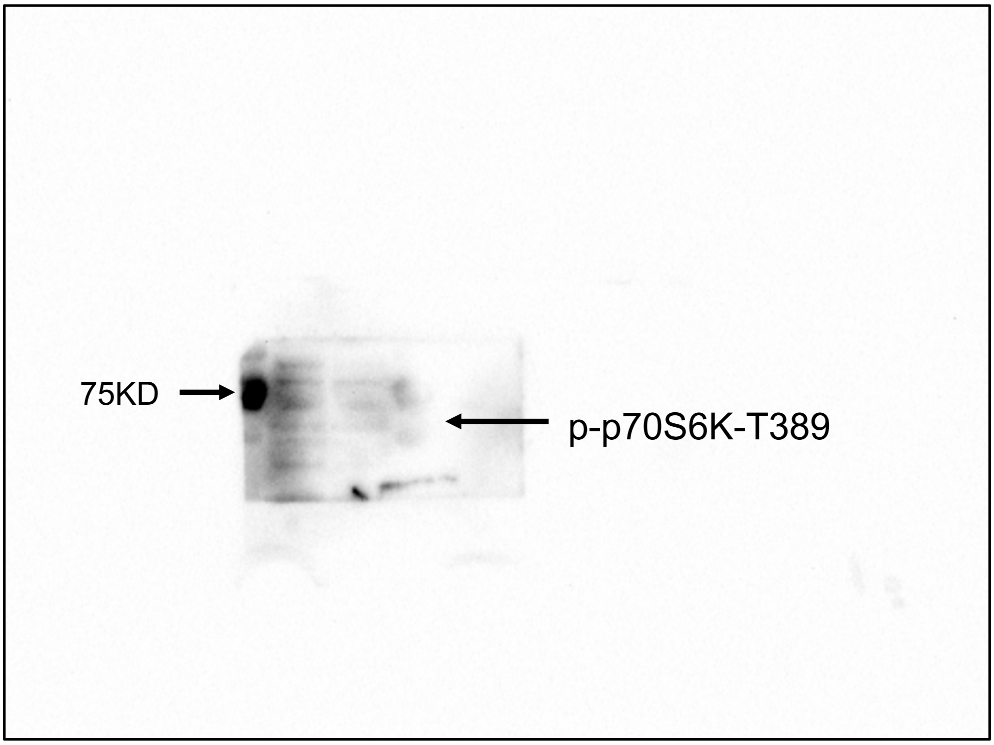

9. Original images of western blots of mTOR and p-mTOR-S2448 in Figure 5D.

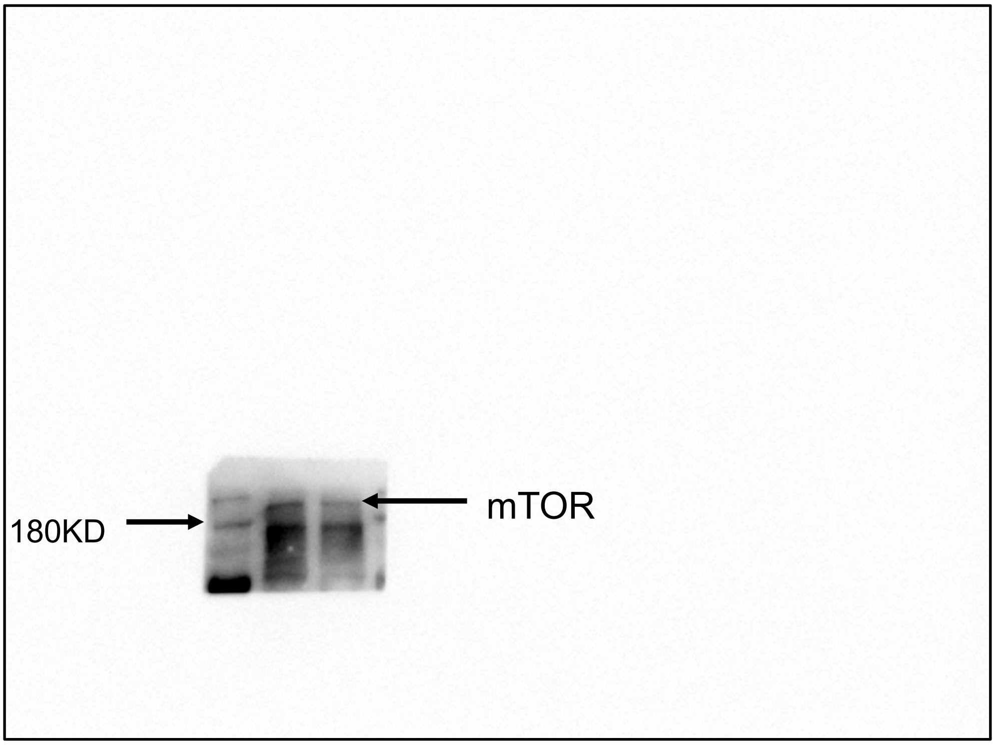

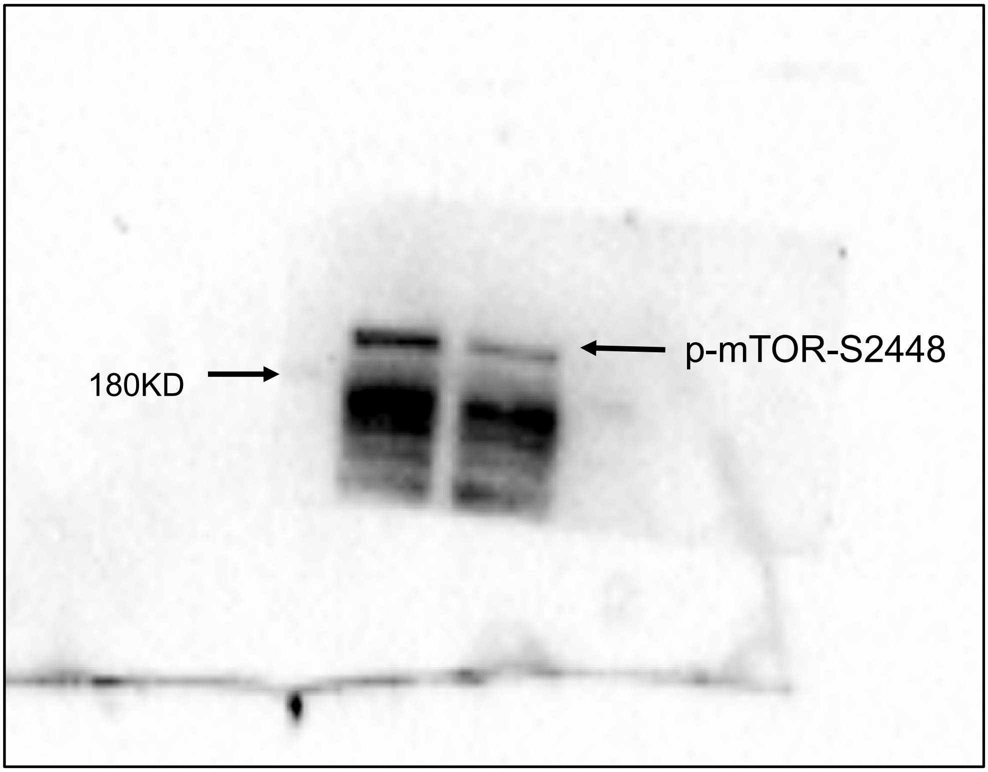

10. Original images of western blots of CA9 and β-actin in Supplementary Figure S1A.

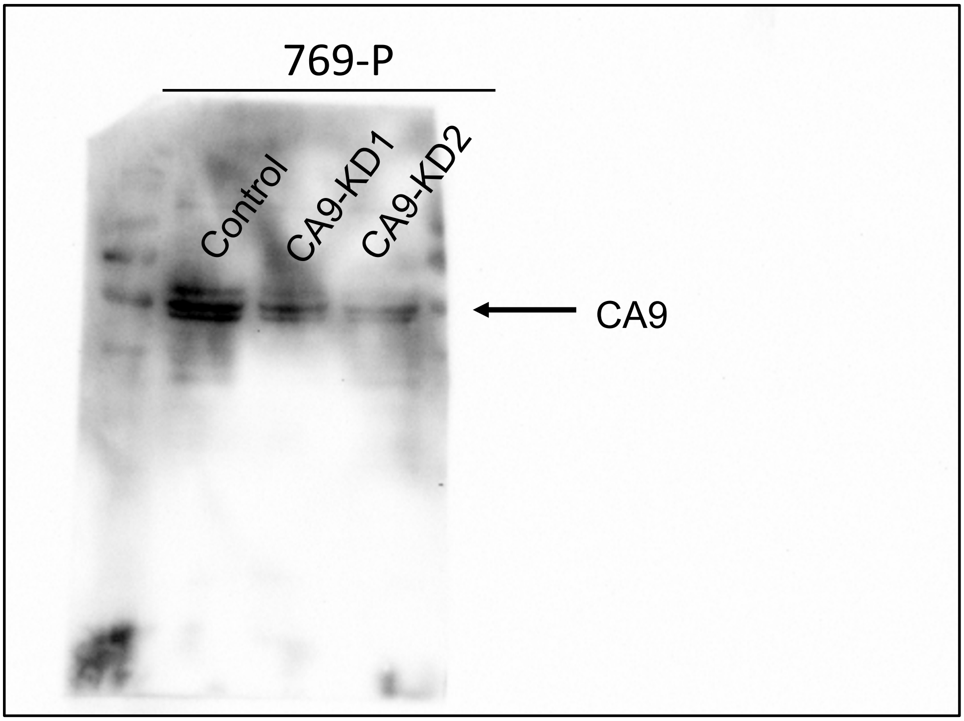

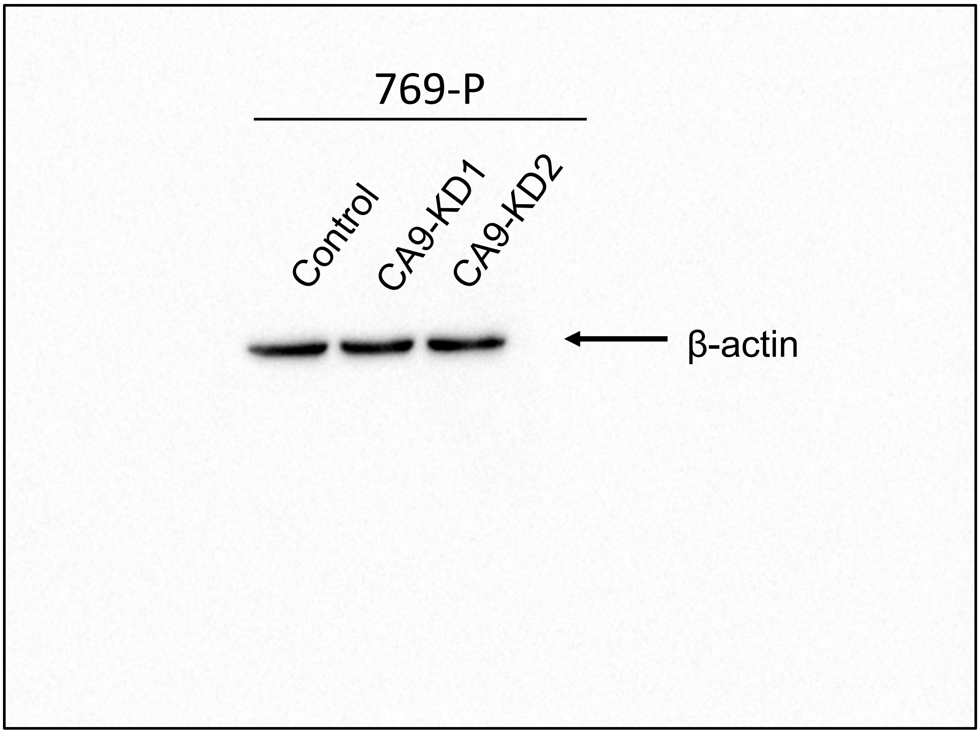

11. Original images of western blots of CA9 and β-actin in Supplementary Figure S1C.

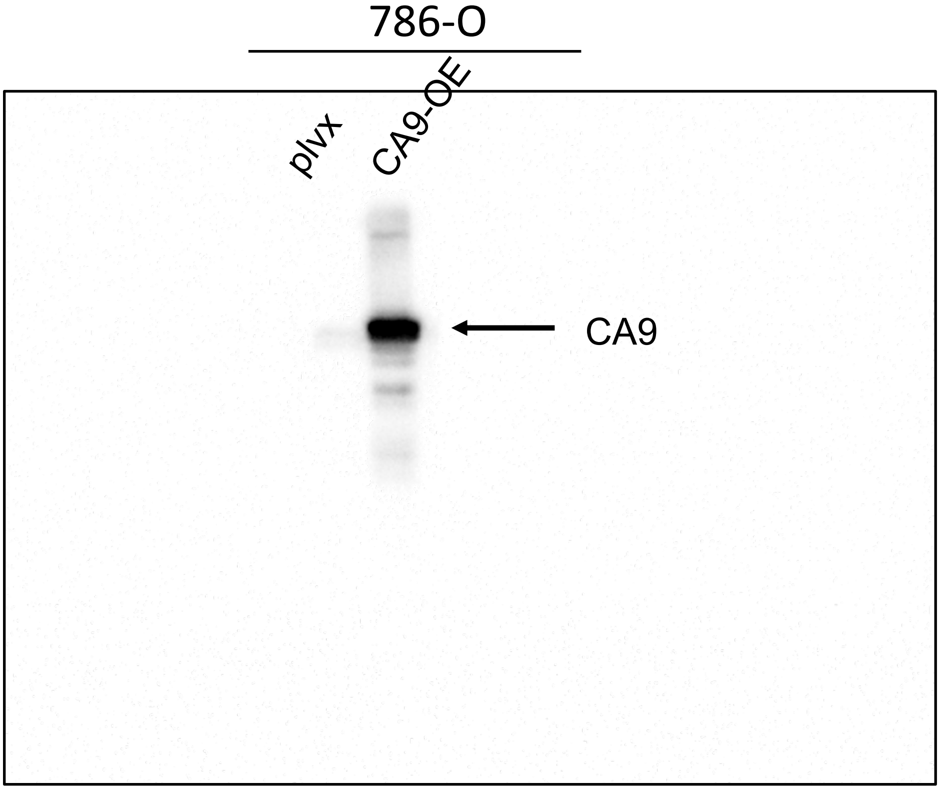

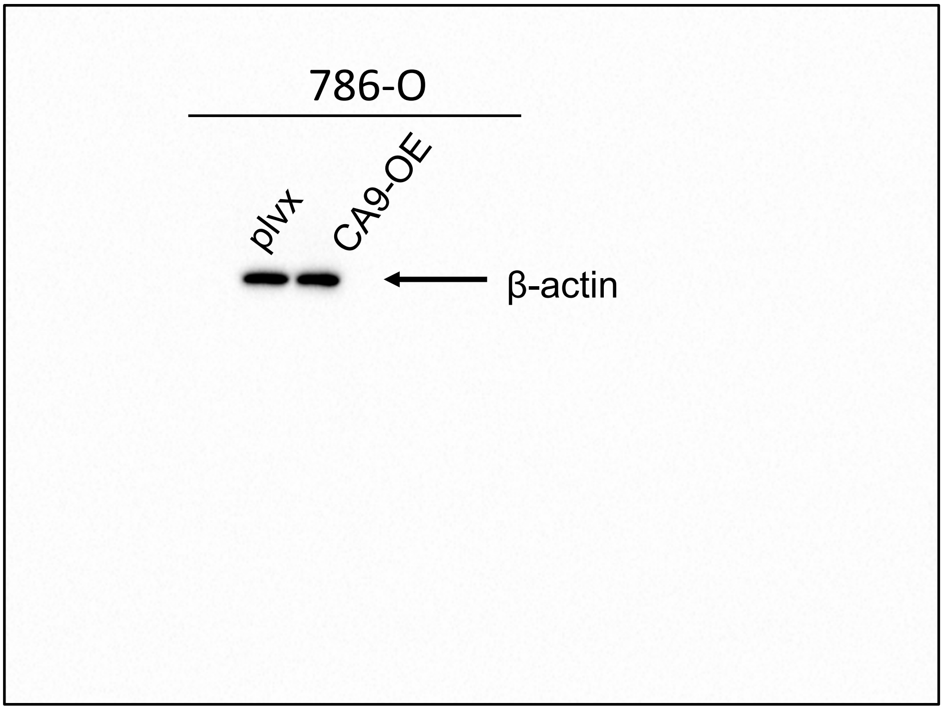

12. Original images of western blots of CA9 and β-actin in Supplementary Figure S1E.

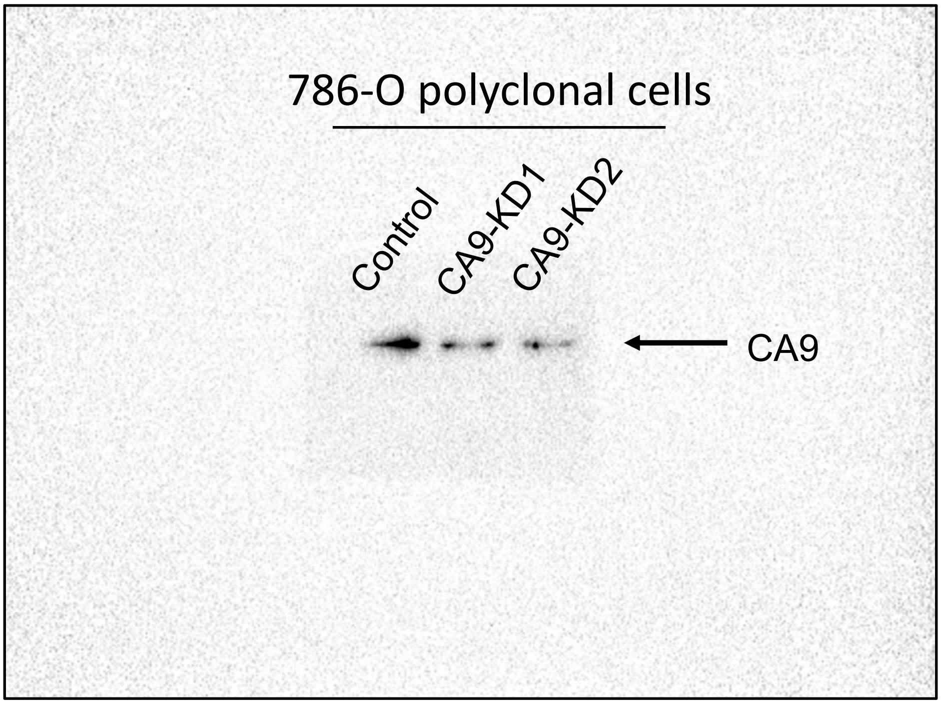

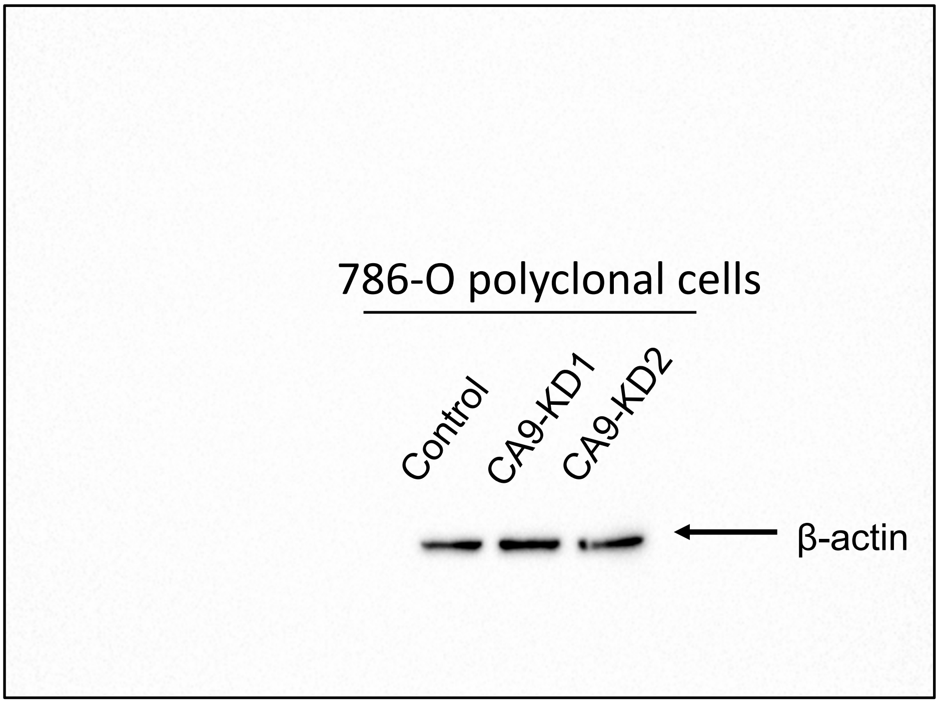

13. Original images of western blots of PGC-1α , ATP5D, NRF2 and ATPAF1 in Supplementary Figure S2B.

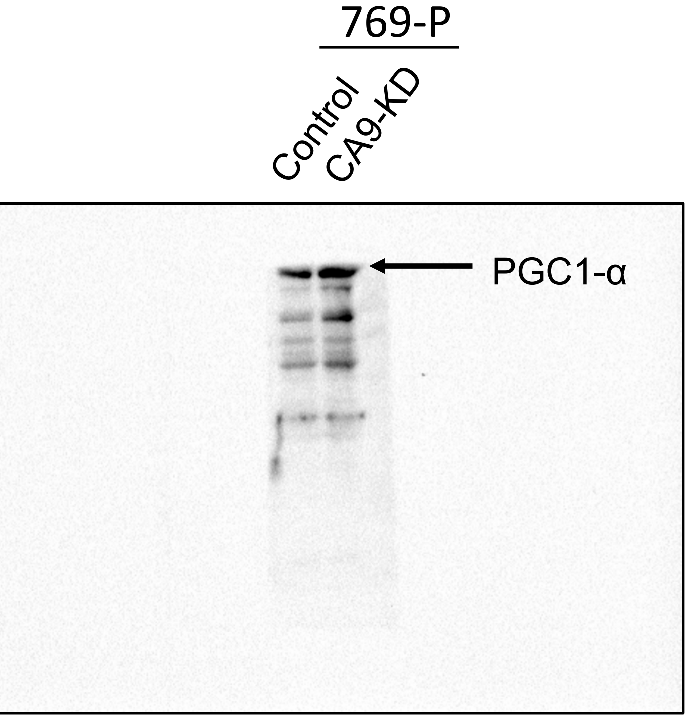

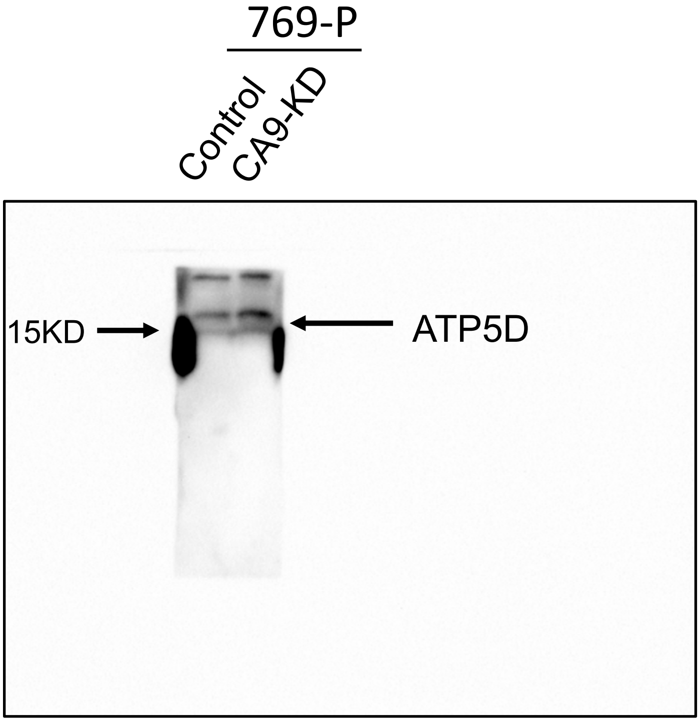

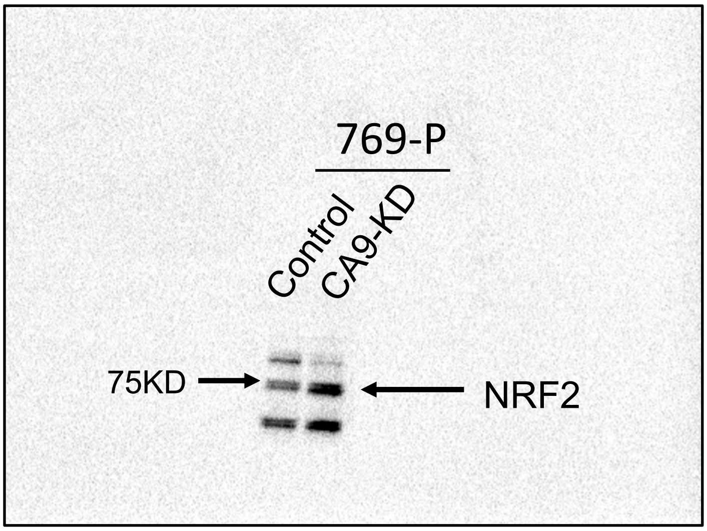

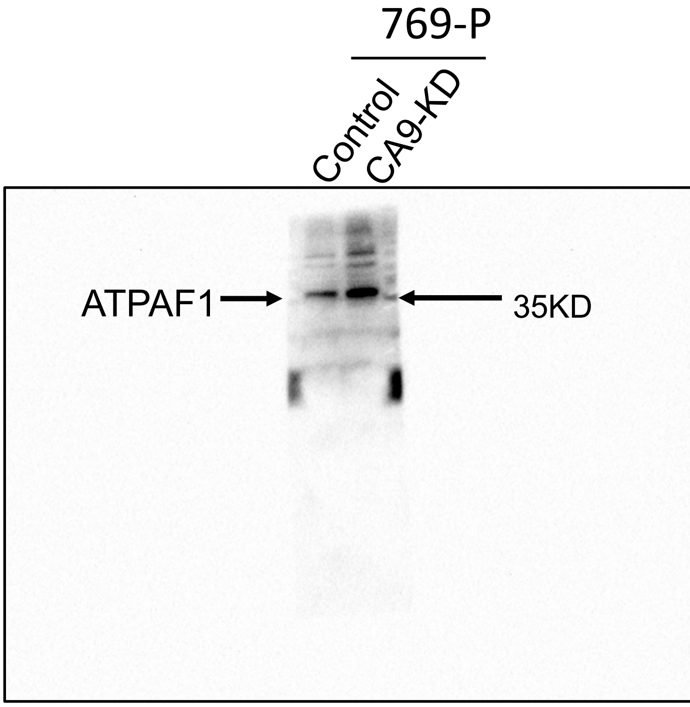

14. Original images of western blots of TFAM and β-actin in Supplementary Figure S2B.

769-P

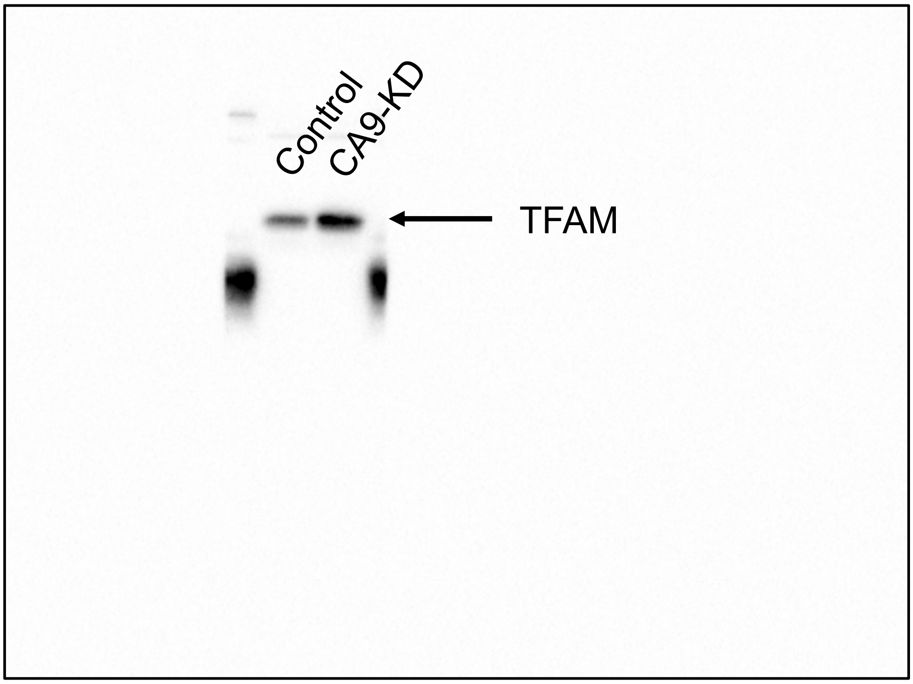

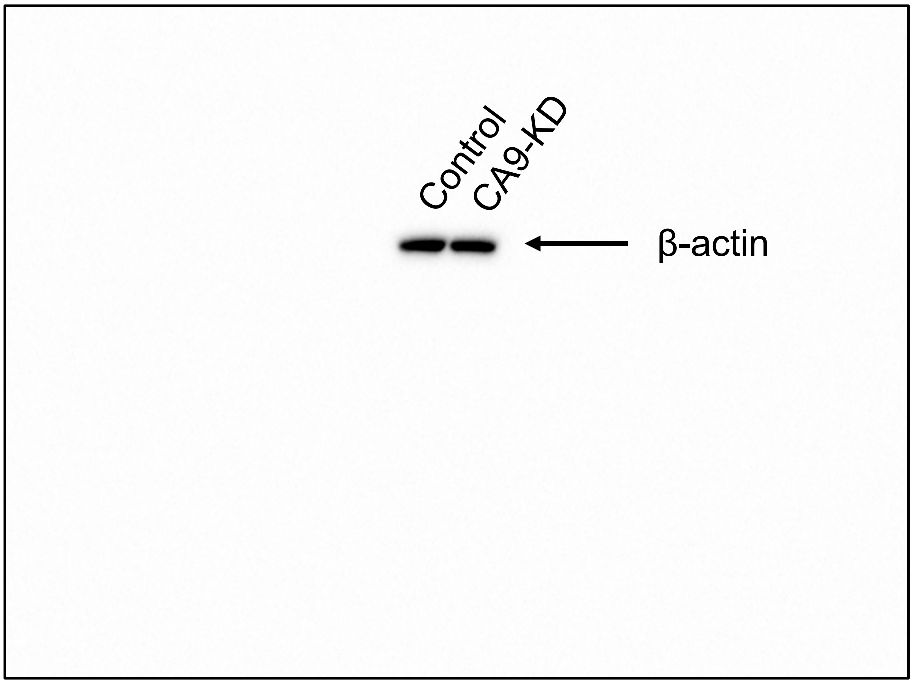

15. Original images of western blots of ARG2, ODC, ASL and β-actin in Supplementary Figure S4A.

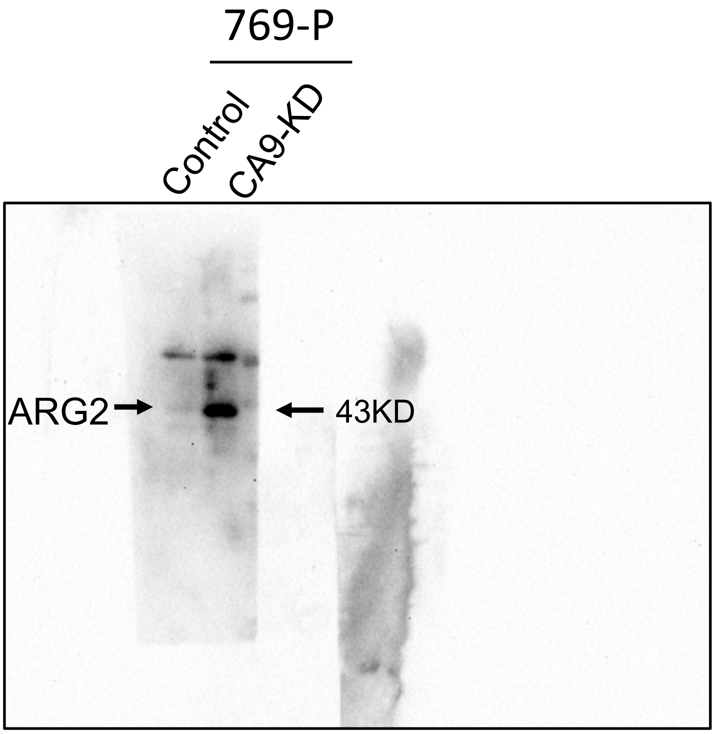

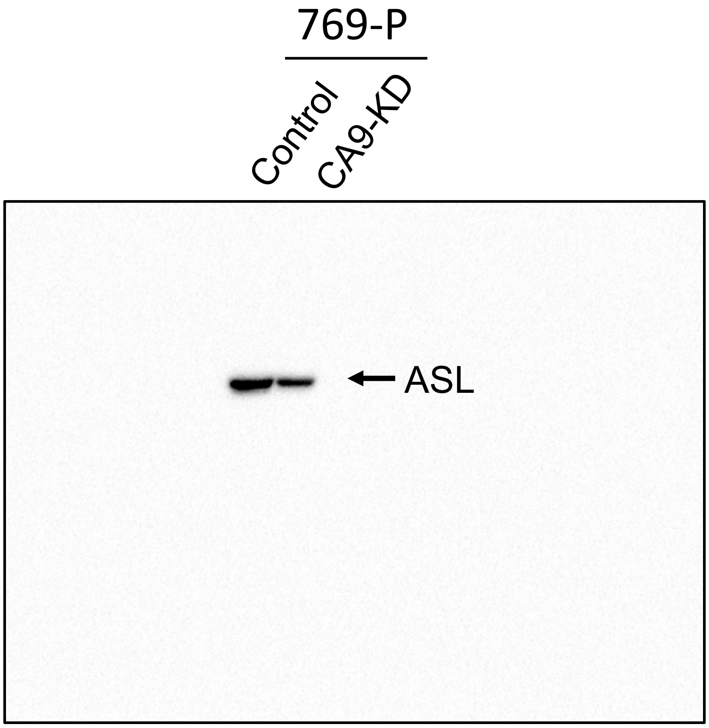

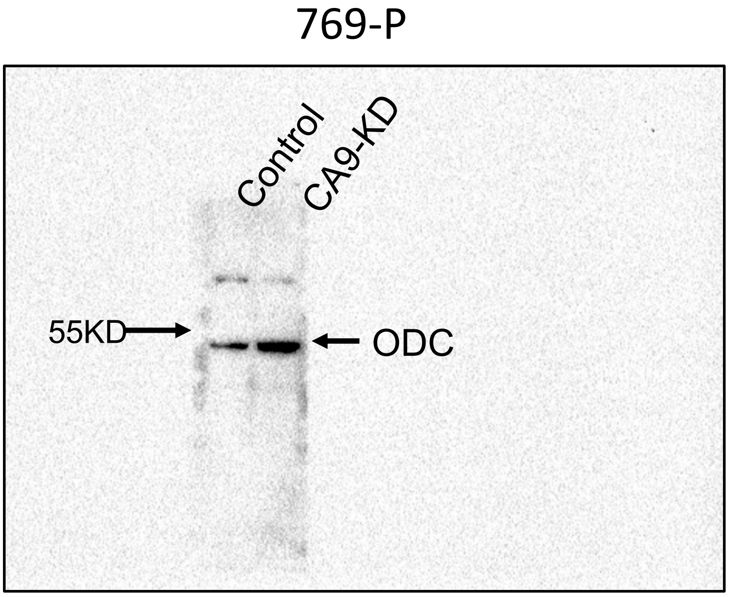

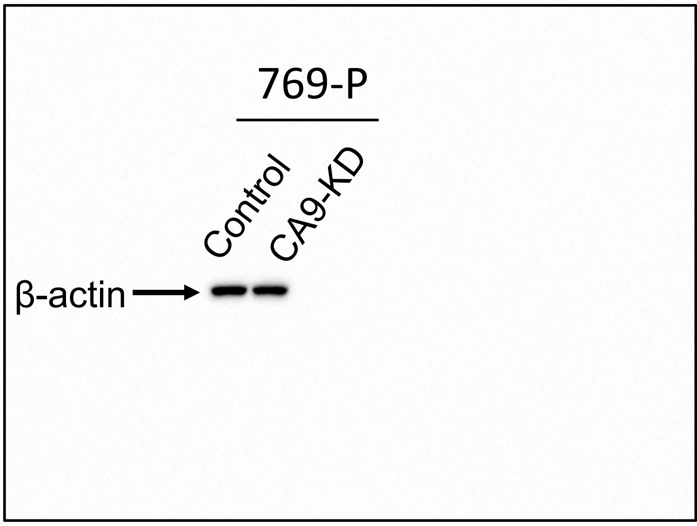

16. Original images of the wound healing assay in Figure 5G.

A. Cells were imaged at 0 h after scratching (n=3).

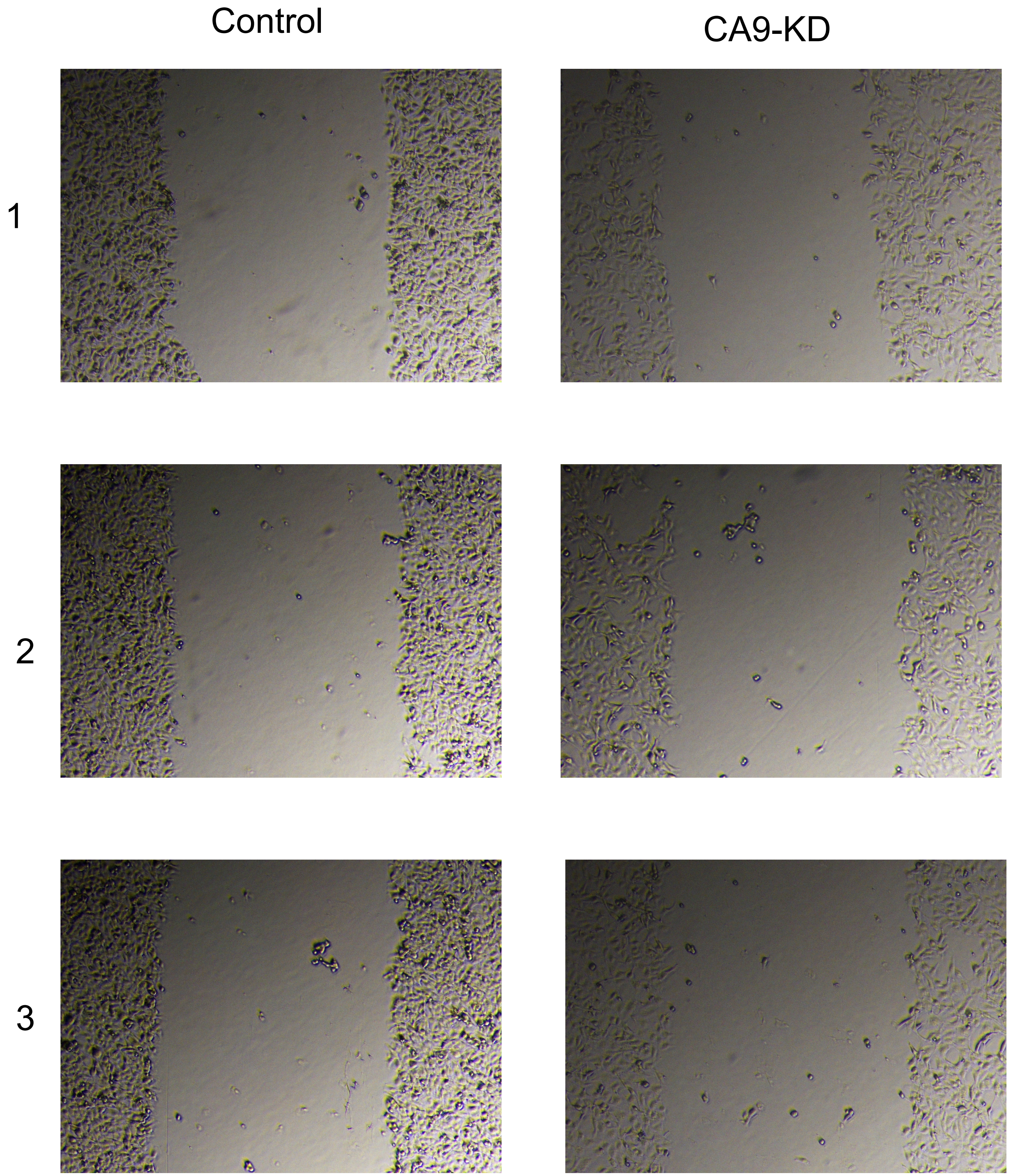

17. Original images of the wound healing assay in Figure 5G.

B. Cells were imaged at 12 h after scratching (n=3).

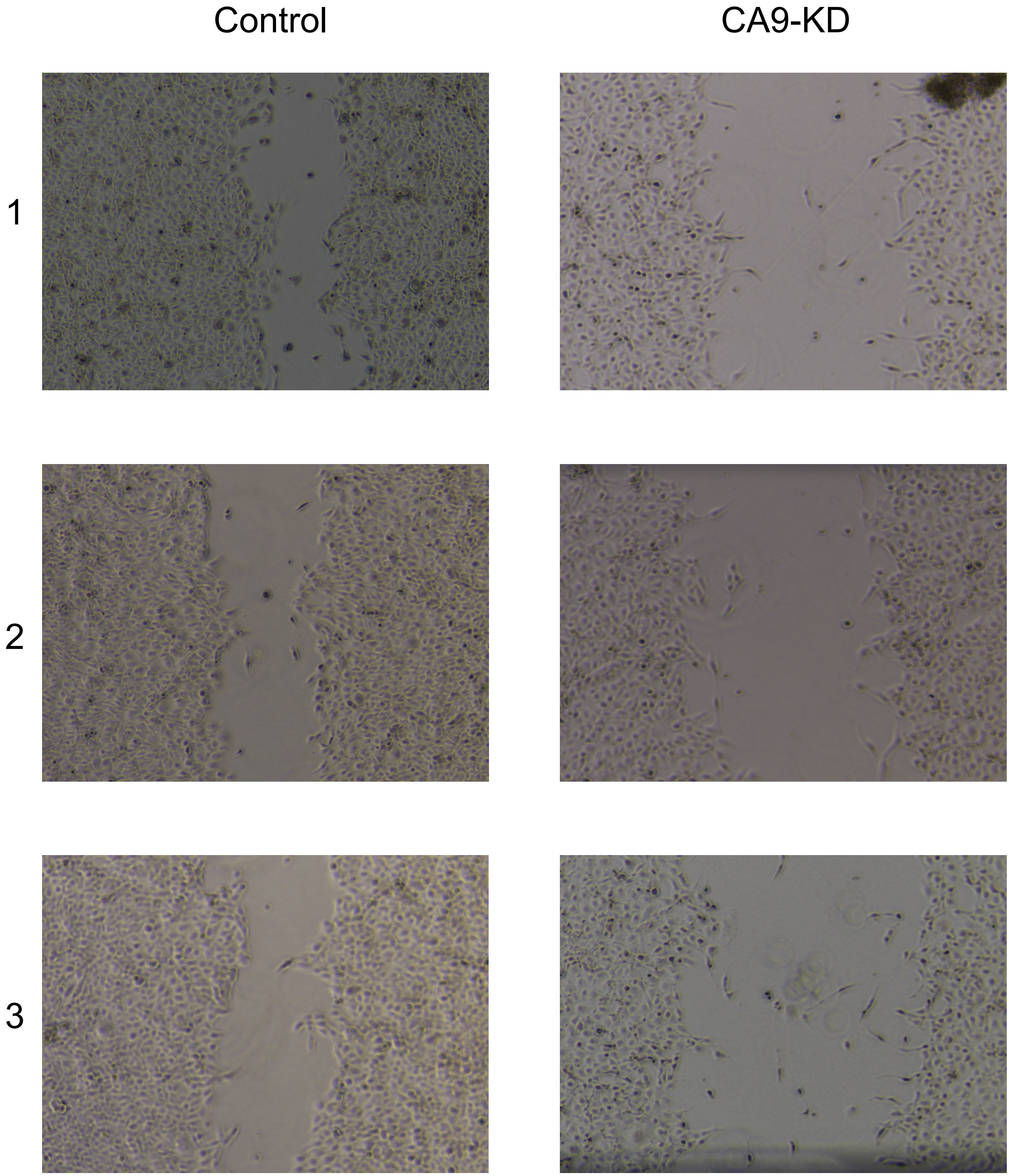

18. Original images of the wound healing assay in Figure 5G.

C. Cells were imaged at 24 h after scratching (n=3).

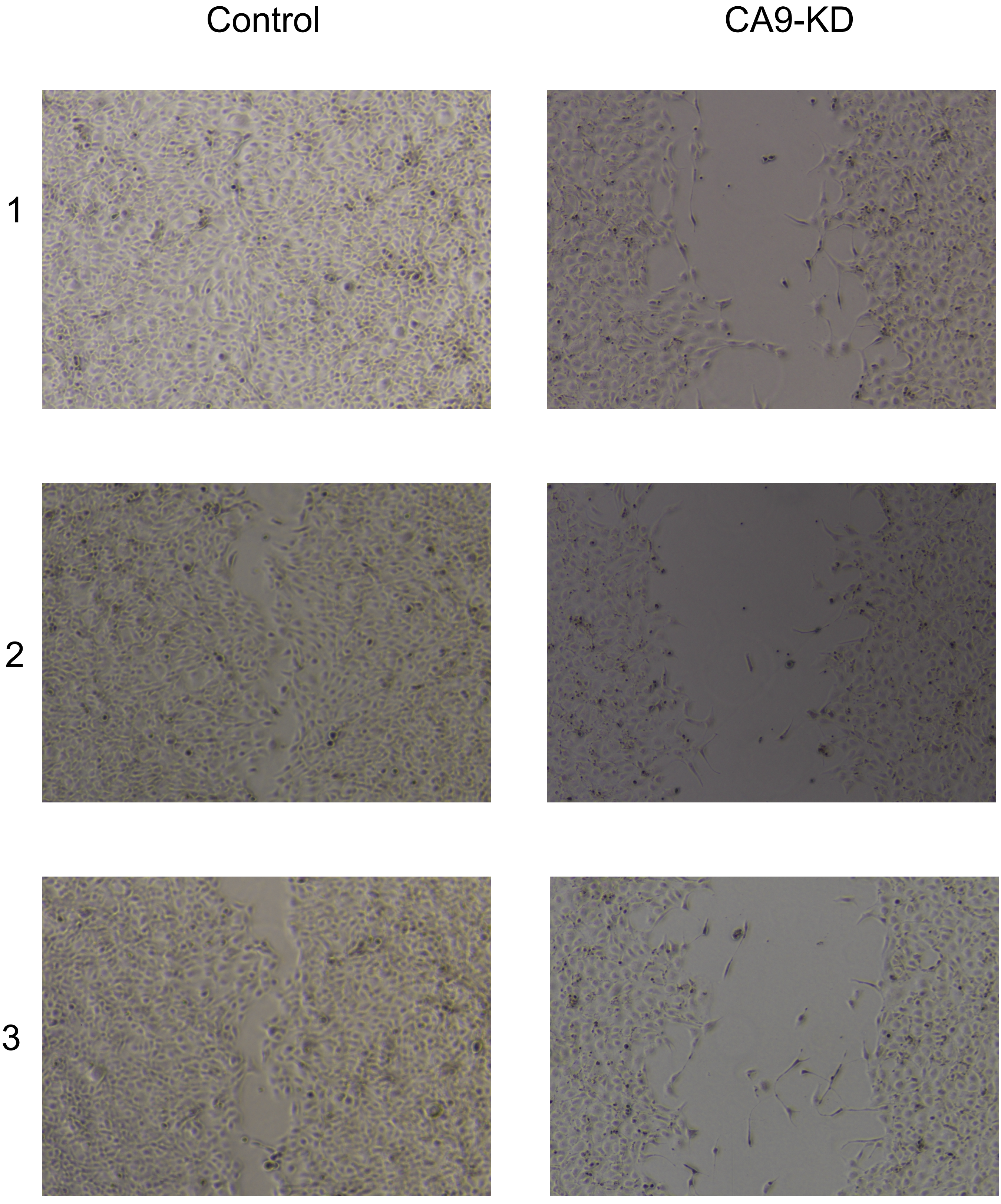

Supplement: Supplementary file 1 [file ijms-21-05939-s001.zip › Supplementary Files/Original images.pdf]
