# Supplementary material for: CA9 Silencing Promotes Mitochondrial Biogenesis, Increases Putrescine Toxicity and Decreases Cell Motility to Suppress ccRCC Progression
Source: Int J Mol Sci. 2020 Aug 18;21(16):5939. doi: 10.3390/ijms21165939 (PMC7460829; doi:10.3390/ijms21165939)
Supplement: Supplementary file 1 [file ijms-21-05939-s001.zip › Supplementary Files/Supplementary Figure new.docx]

*CA9* Silencing Promotes Mitochondrial Biogenesis, Increases Putrescine Toxicity and Decreases Cell Motility to Suppress ccRCC Progression

Jiatong Xu ^1^, Songbiao Zhu ^1^, Lina Xu ^1^, Xiaohui Liu ^1^, Wenxi Ding ^1^, Qingtao Wang ^2^, Yuling Chen ^1,^* and Haiteng Deng ^1,^*

^1^ MOE Key Laboratory of Bioinformatics, Center for Synthetic and Systematic Biology, School of Life Sciences, Tsinghua University, Beijing, 100084, China; [xjt15@mails.tsinghua.edu.cn](mailto:xjt15@mails.tsinghua.edu.cn) (J.X.); [songbiao_zhu@163.com](mailto:songbiao_zhu@163.com) (S.Z.); [xulina@mail.tsinghua.edu.cn](mailto:xulina@mail.tsinghua.edu.cn) (L.X.); [xiaohuiliu@biomed.tsinghua.edu.cn](mailto:xiaohuiliu@biomed.tsinghua.edu.cn) (X.L.); [dwx18@mails.tsinghua.edu.cn](mailto:dwx18@mails.tsinghua.edu.cn) (W.D.); [chenyuling2016@mail.tsinghua.edu.cn](mailto:chenyuling2016@mail.tsinghua.edu.cn) (Y.C.); [dht@mail.tsinghua.edu.cn](mailto:dht@mail.tsinghua.edu.cn) (H.D.)

2 Beijing Chaoyang Hospital Affiliated to Capital Medical University, Beijing, 100043, China; [wqt36@163.com](mailto:wqt36@163.com) (Q.W.)

* Correspondence: [chenyuling2016@mail.tsinghua.edu.cn](mailto:chenyuling2016@mail.tsinghua.edu.cn) (Y.C.); [dht@mail.tsinghua.edu.cn](mailto:dht@mail.tsinghua.edu.cn) (H.D.); Tel.: +8610-62797838 (Y.C.); +8610-62790498 (H.D.)


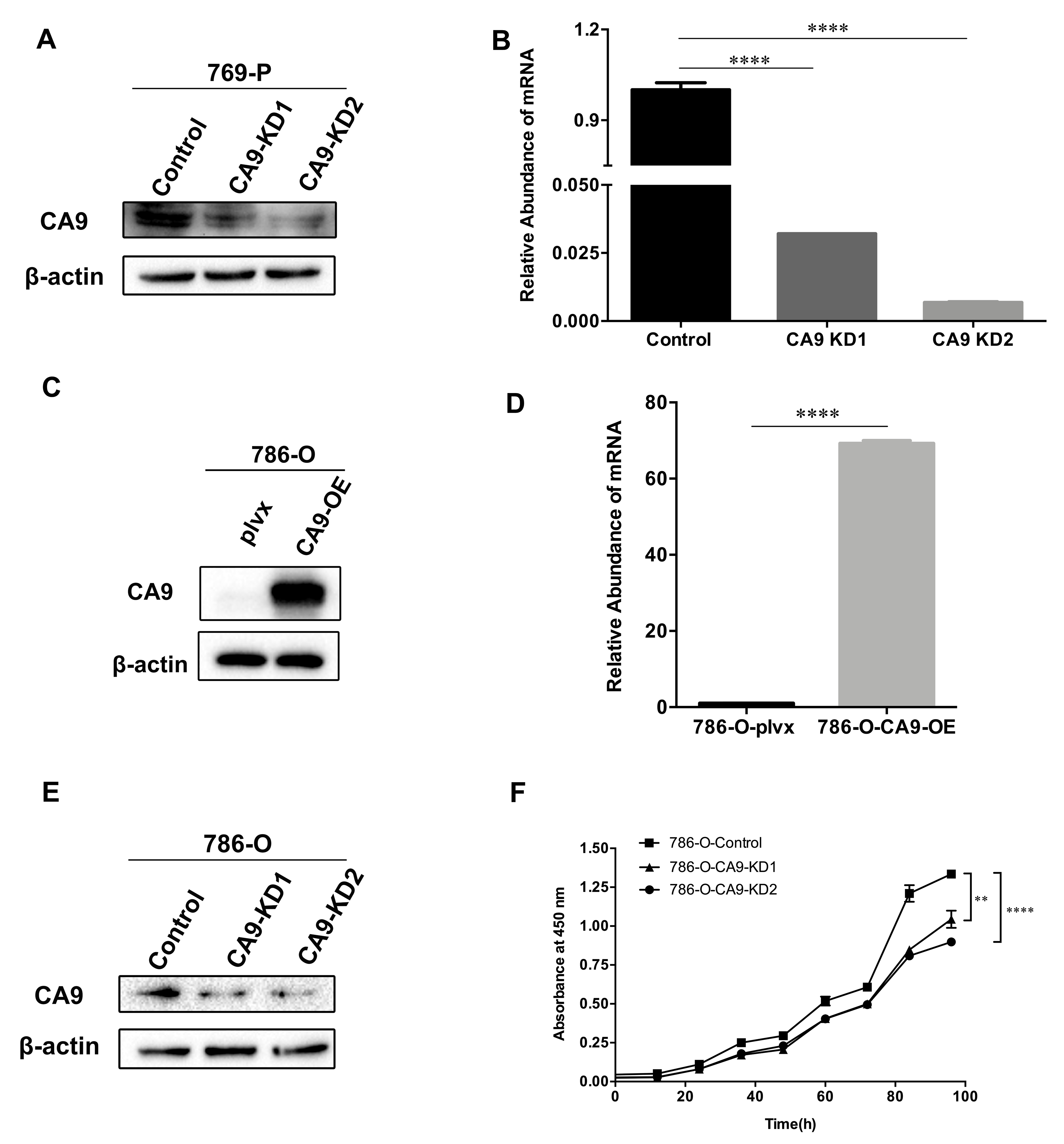


**Figure S1.** **The confirmation of *CA9* knockdown and overexpression in 786-O and 769-P cells.** (A) Western blotting of CA9 revealed that the expression of CA9 was reduced in 769-P *CA9* knockdown (769-P-CA9-KD) cells. β-actin was used as control. (B) mRNA expression of *CA9* decreased in 769-P-CA9-KD cells compared with control cells, measured by qPCR (n = 3, mean ± SEM). *ACTB* was used as control. (C) Western blotting of CA9 revealed that CA9 was overexpressed in 786-O *CA9* overexpressed (786-O-CA9-OE) cells. β-actin was used as control. (D) mRNA expression of *CA9* increased in 786-O-CA9-OE cells compared with control cells (786-plvx), measured by quantitative real-time PCR (qPCR). *ACTB* was used as control (n = 3, mean ± SEM). (E) Western blotting of CA9 revealed that CA9 was reduced in 786-O-CA9-KD polyclonal cells. β-actin was used as control. (D) Knockdown of *CA9* in 786-O polyclonal cells inhibited cell growth compared with control cells. Cell proliferation curves measured by the Cell Counting Kit-8 (CCK-8) assays (n = 4, mean ± SEM). Significance was calculated by the Student’s t-test. *****p* < 0.0001, ***p* < 0.01.

**
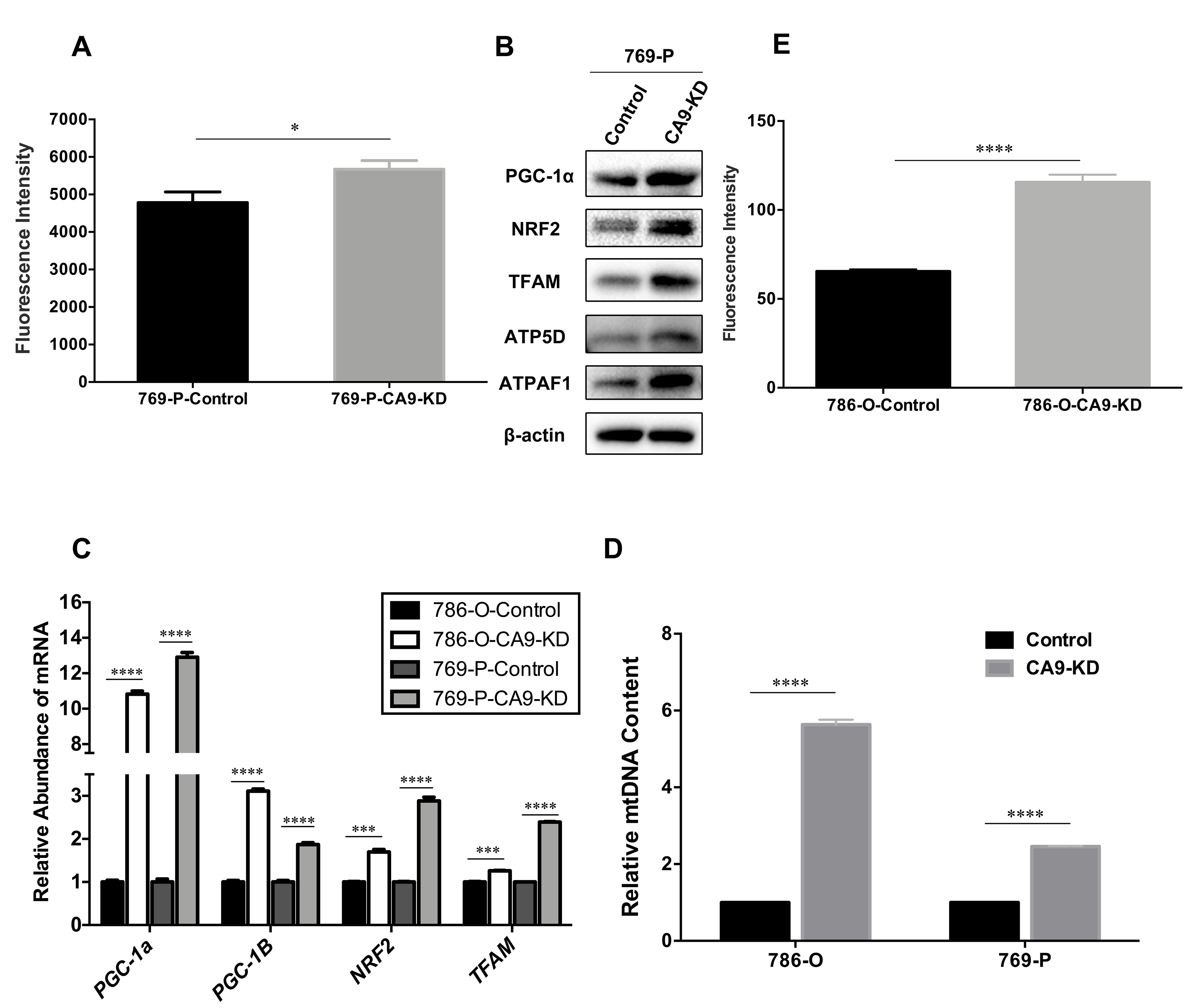
**

**Figure S2. *CA9* knockdown increases mitochondrial biogenesis in 786-O and 769 cells.** (A) *CA9* knockdown increased MitoTracker staining intensity in 769-P cells. (B) Western blotting and (C) qPCR of peroxisome proliferator-activated receptor gamma coactivator 1-alpha (PGC-1α), nuclear factor erythroid 2-related factor 2 (NRF2) and mitochondrial transcription factor 1 (TFAM) showed that *CA9* knockdown enhanced the expressions of key factors in mitochondrial biogenesis. (D) mitochondrial DNA (mtDNA) measurements normalized to nuclear DNA in control and CA9-KD 786-O and 769-P cells. (E) *CA9* silencing enhances the cellular ROS level. Significance was calculated by the Student’s t-test. *****p* < 0.0001, ****p* < 0.001, **p* < 0.05; n = 3, mean ± SEM.


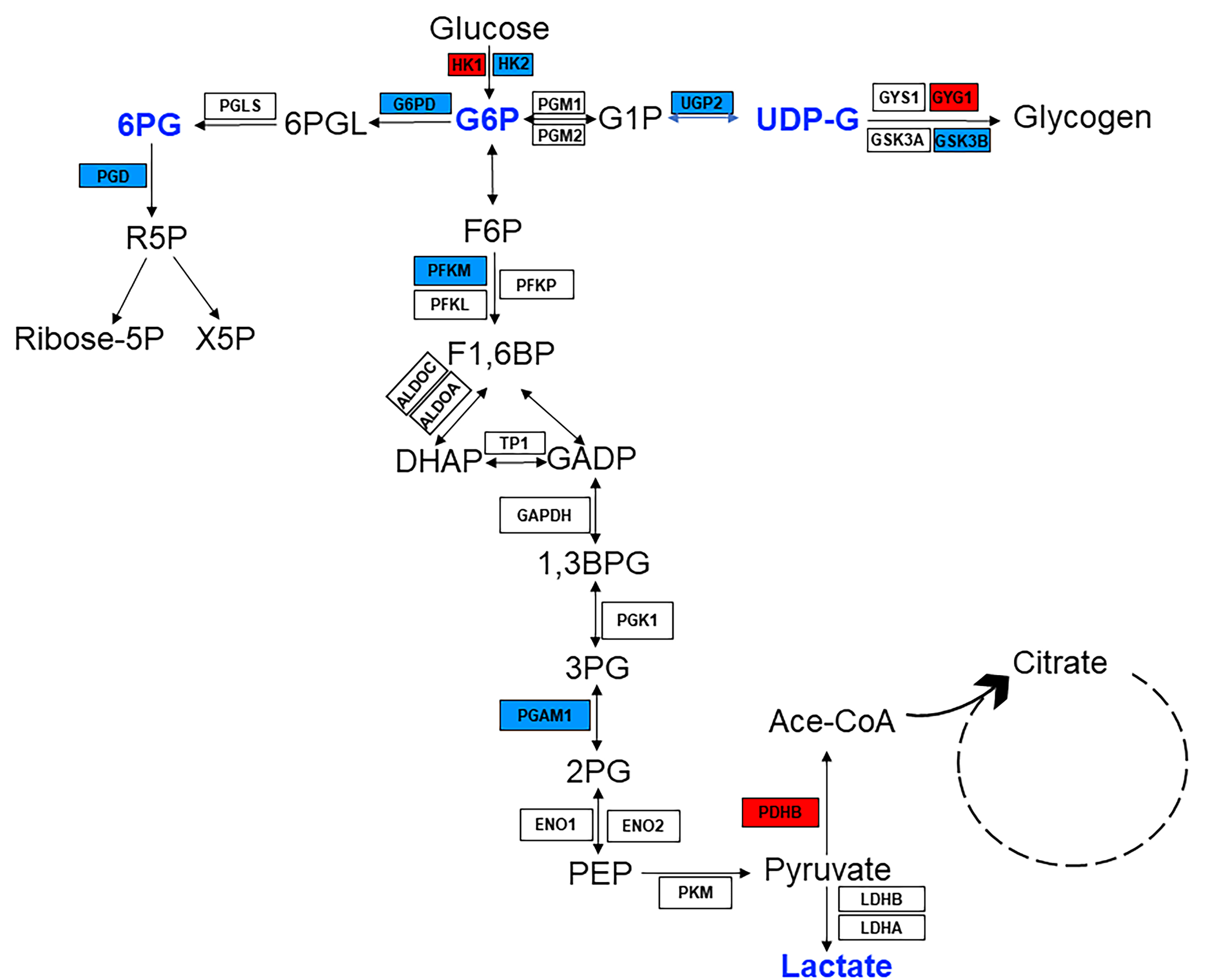


**Figure S3. *CA9* knockdown decreased enzymes and metabolites of glycolysis.** Blue- and red- color-coded rectangles represent for downregulated and upregulated proteins. Blue fonts represent for downregulated metabolites with significant difference.


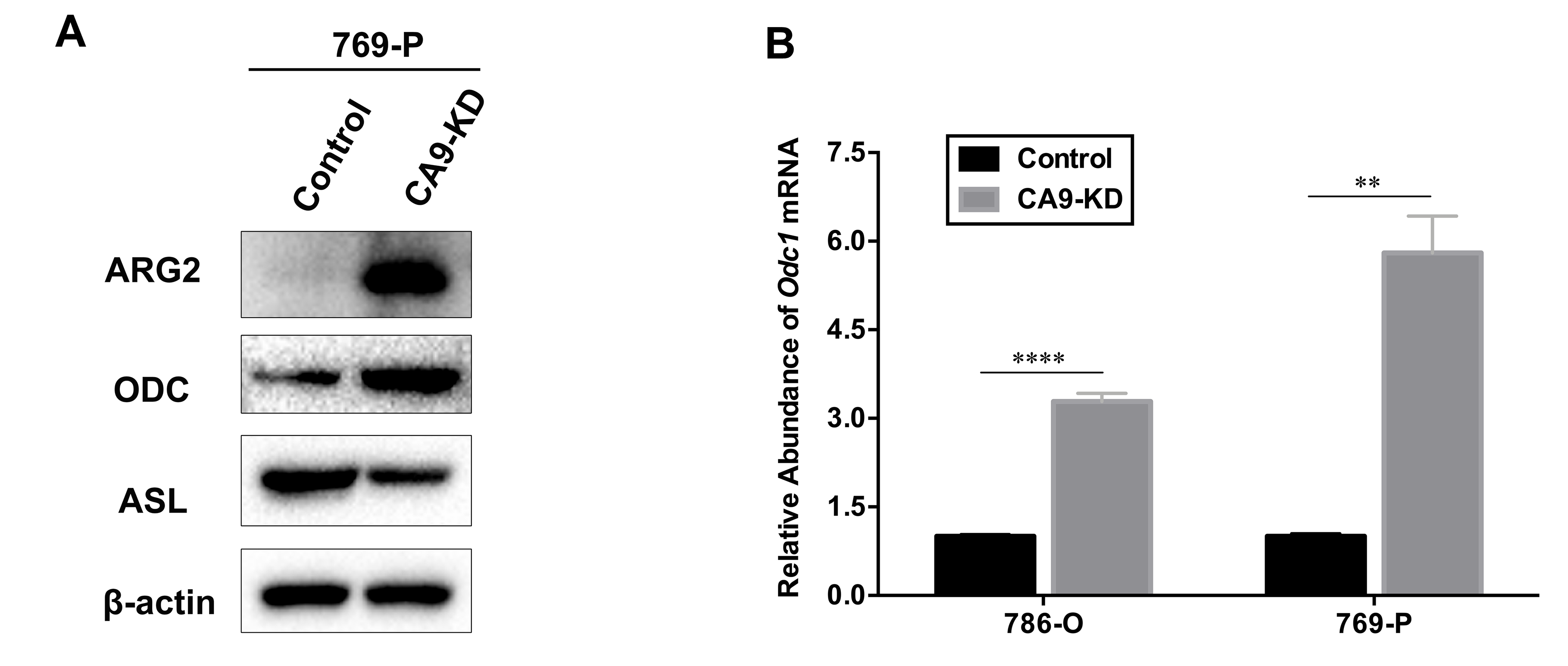


**Figure S4. The expression of the enzymes involved in the metabolism of arginine in control and CA9-KD 786-O and 769-P cells.** (A) Arginase 2 (ARG2) and ornithine decarboxylase (ODC) were upregulated, while argininosuccinate lyase (ASL) was downregulated after *CA9* knockdown in 769-P cells, confirmed by Western blotting. β-actin was used as control. (B) mRNA expression of *Odc1* increased in CA9-KD cells compared with control cells, measured by qPCR. *ACTB* was used as control. Significance was calculated by the Student’s t-test. *****p* < 0.0001, ***p* < 0.01; n = 3, mean ± SEM.


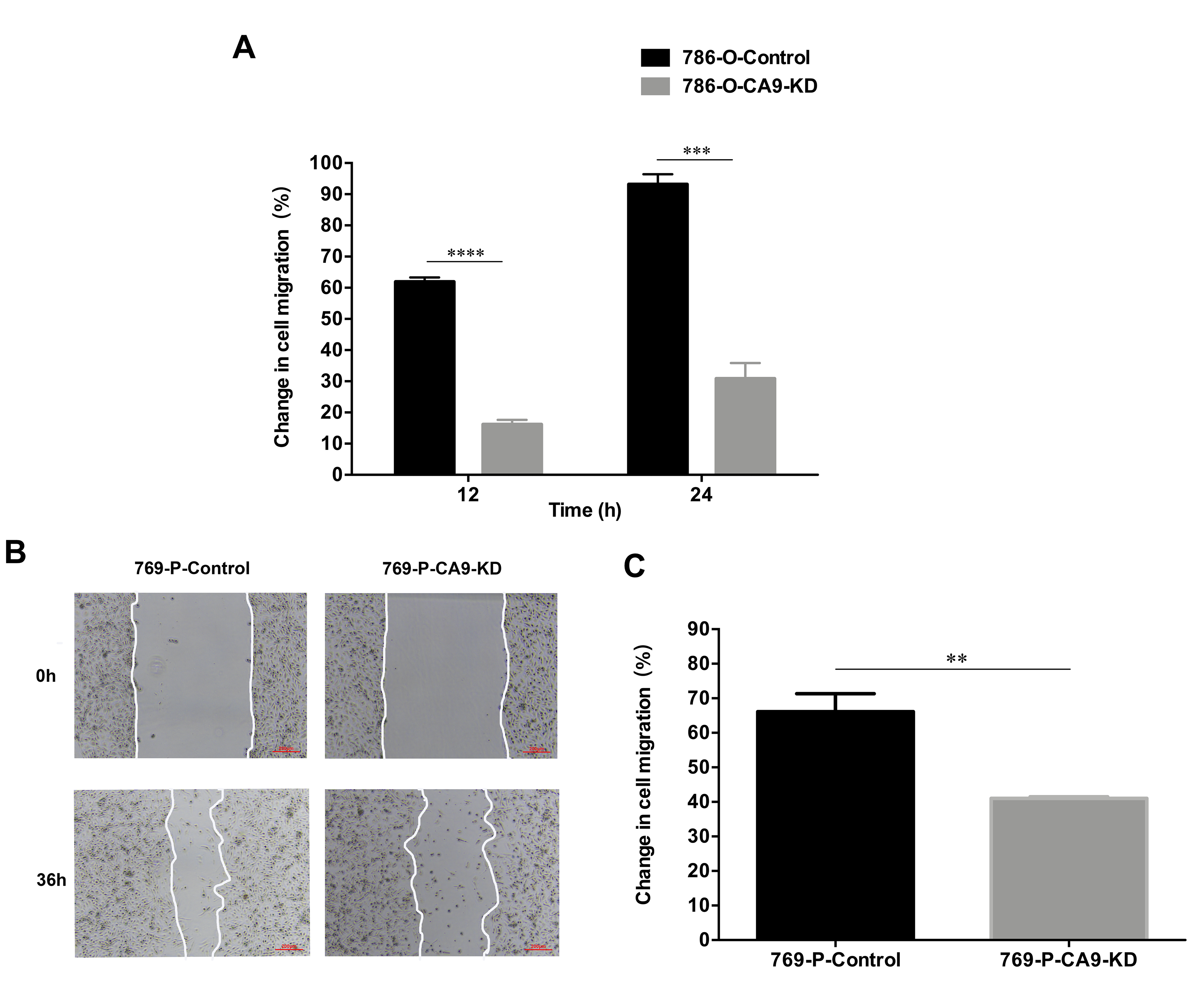


**Figure S5. *CA9* silencing inhibited cell migration.** (A) Quantification of migration by the 786-O-Control and 786-O-CA9-KD cells in the wound healing assay. (B) Cell migration was inhibited in 769-P-CA9-KD cells compared with that in control cells, assessed by the wound healing assay. Cells were imaged at 0 and 36 h after scratching. Scale bar: 200 μm. (C) Quantification of migration by the 769-P -Control and 769-P-CA9-KD cells in the wound healing assay. The wound area was quantitatively evaluated with ImageJ software (National Institutes of Health, Bethesda, MD, USA). Significance was calculated by the Student’s t-test. *****p* < 0.0001, ****p* < 0.001, ***p* < 0.01; n = 3, mean ± SEM.


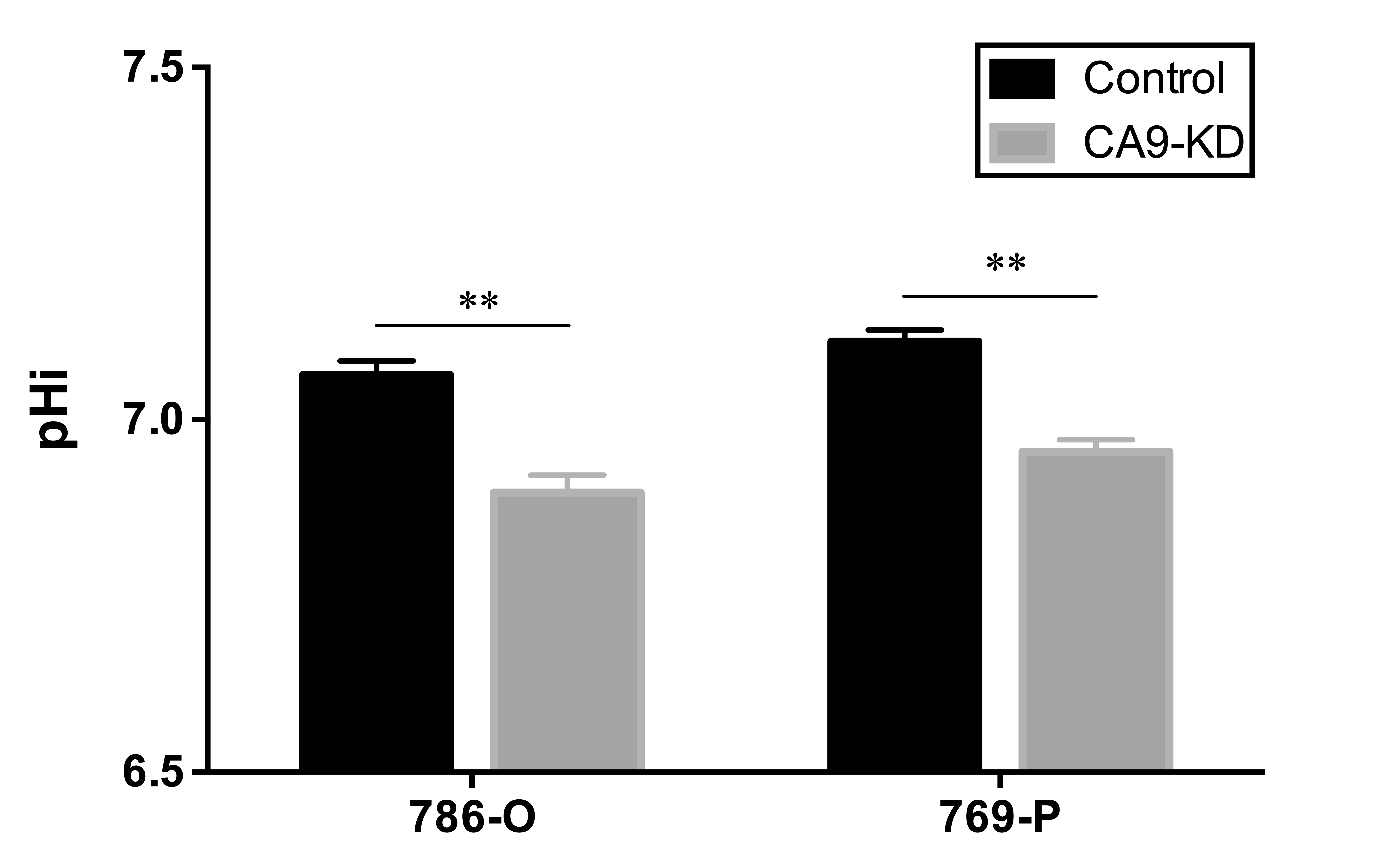


**Figure S6. *CA9* knockdown reduced the intracellular pH (pHi) in 786-O and 769-P cells.** Significance was calculated by the Student’s t-test. **p < 0.01; n = 3, mean ± SEM.
