# Supplementary figures and images for: CA9 Silencing Promotes Mitochondrial Biogenesis, Increases Putrescine Toxicity and Decreases Cell Motility to Suppress ccRCC Progression
Source: Int J Mol Sci. 2020 Aug 18;21(16):5939. doi: 10.3390/ijms21165939 (PMC7460829; doi:10.3390/ijms21165939)

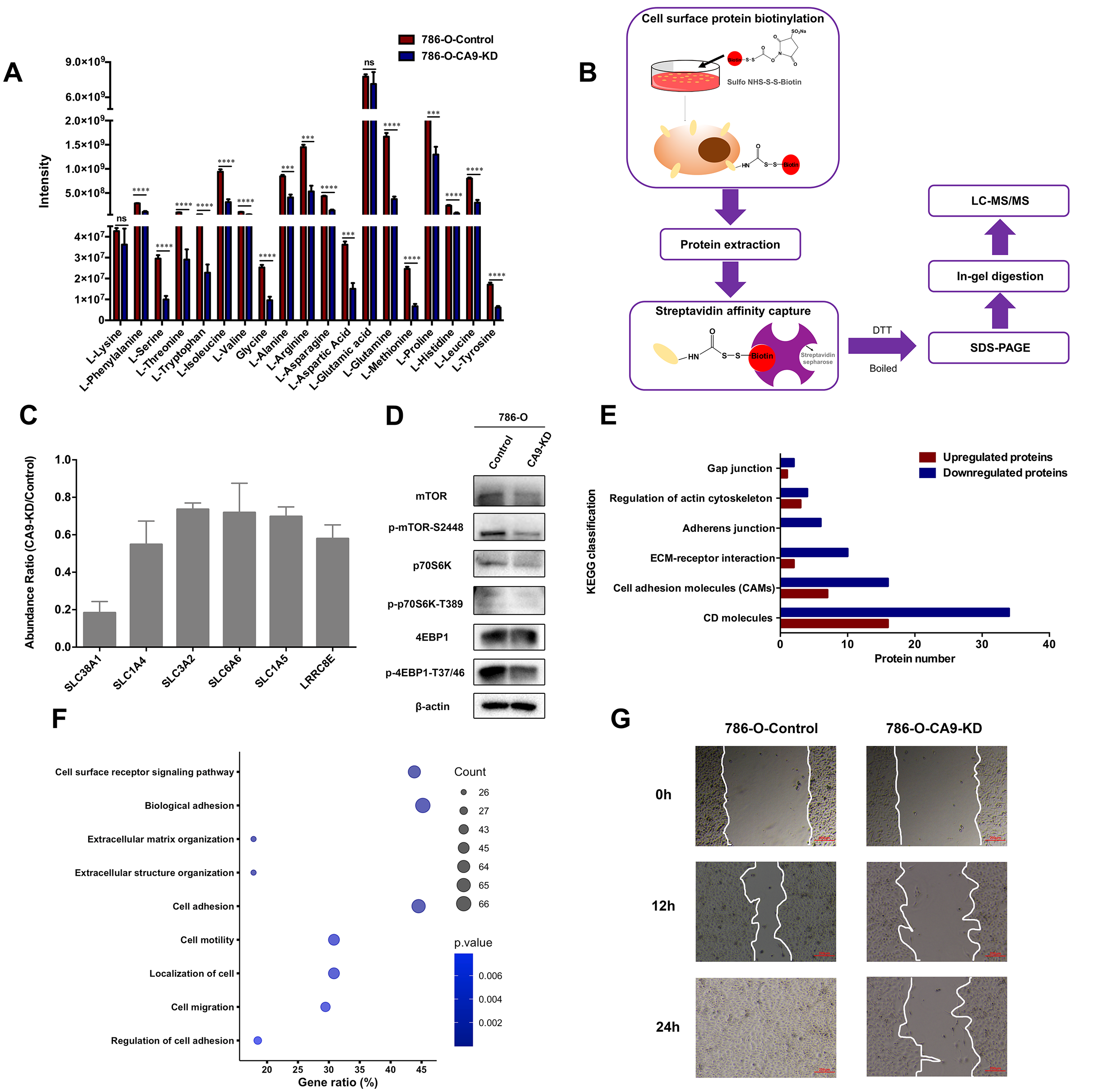

Supplement: Supplementary file 1 [file ijms-21-05939-s001.zip › Supplementary Files/Figures/Figure 5.tif]

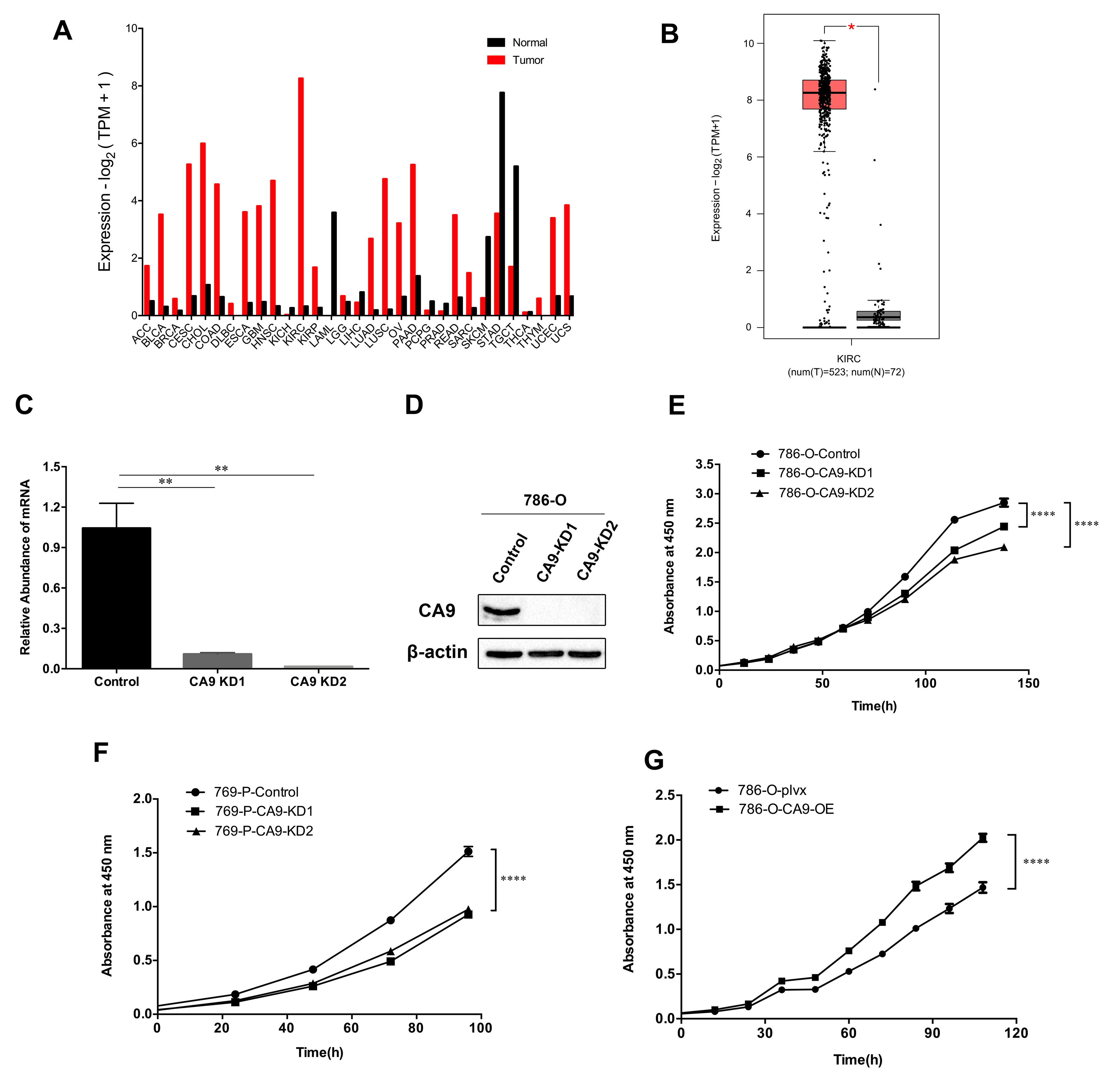

Supplement: Supplementary file 1 [file ijms-21-05939-s001.zip › Supplementary Files/Figures/Figure 1.tif]

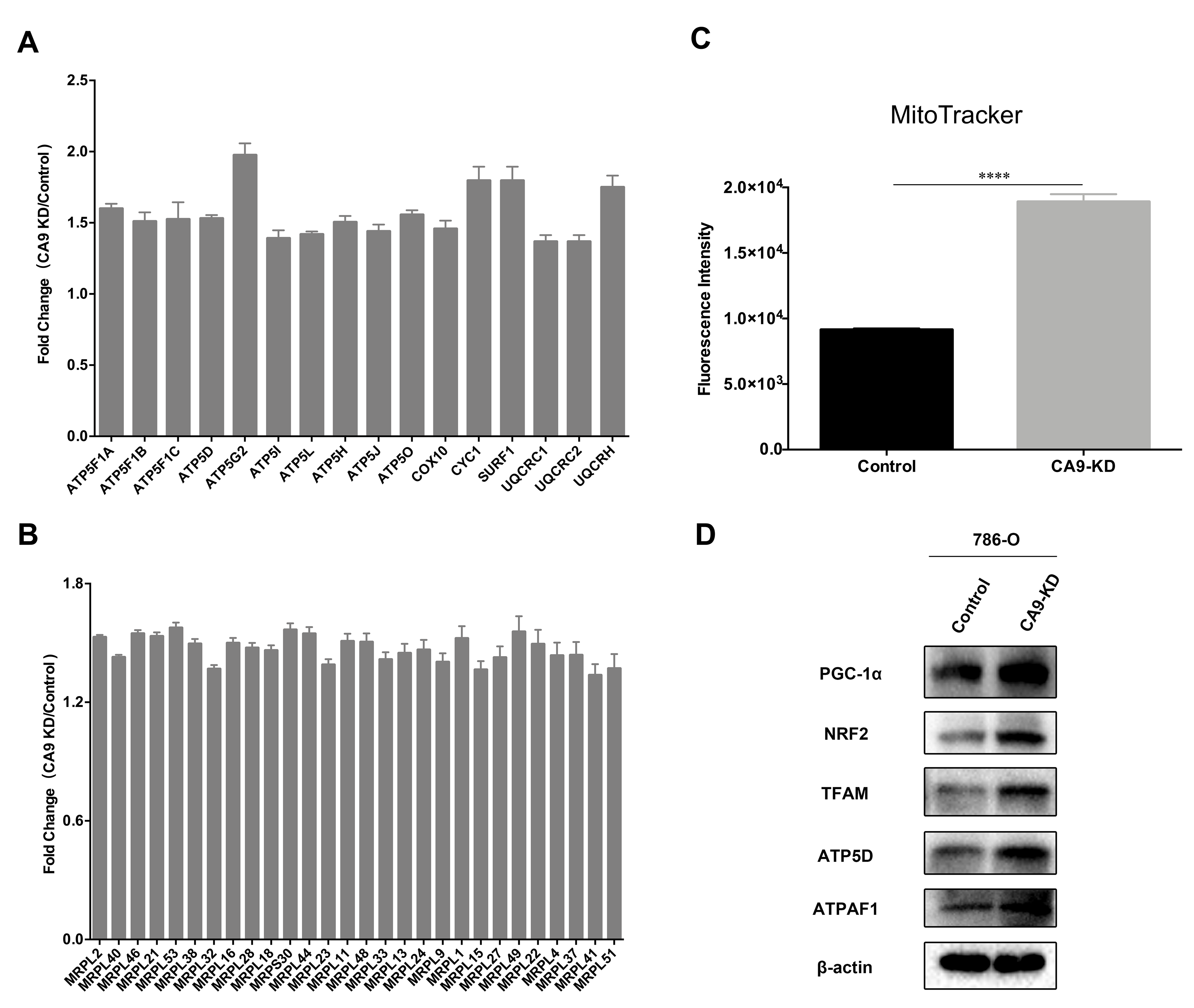

Supplement: Supplementary file 1 [file ijms-21-05939-s001.zip › Supplementary Files/Figures/Figure 3.tif]

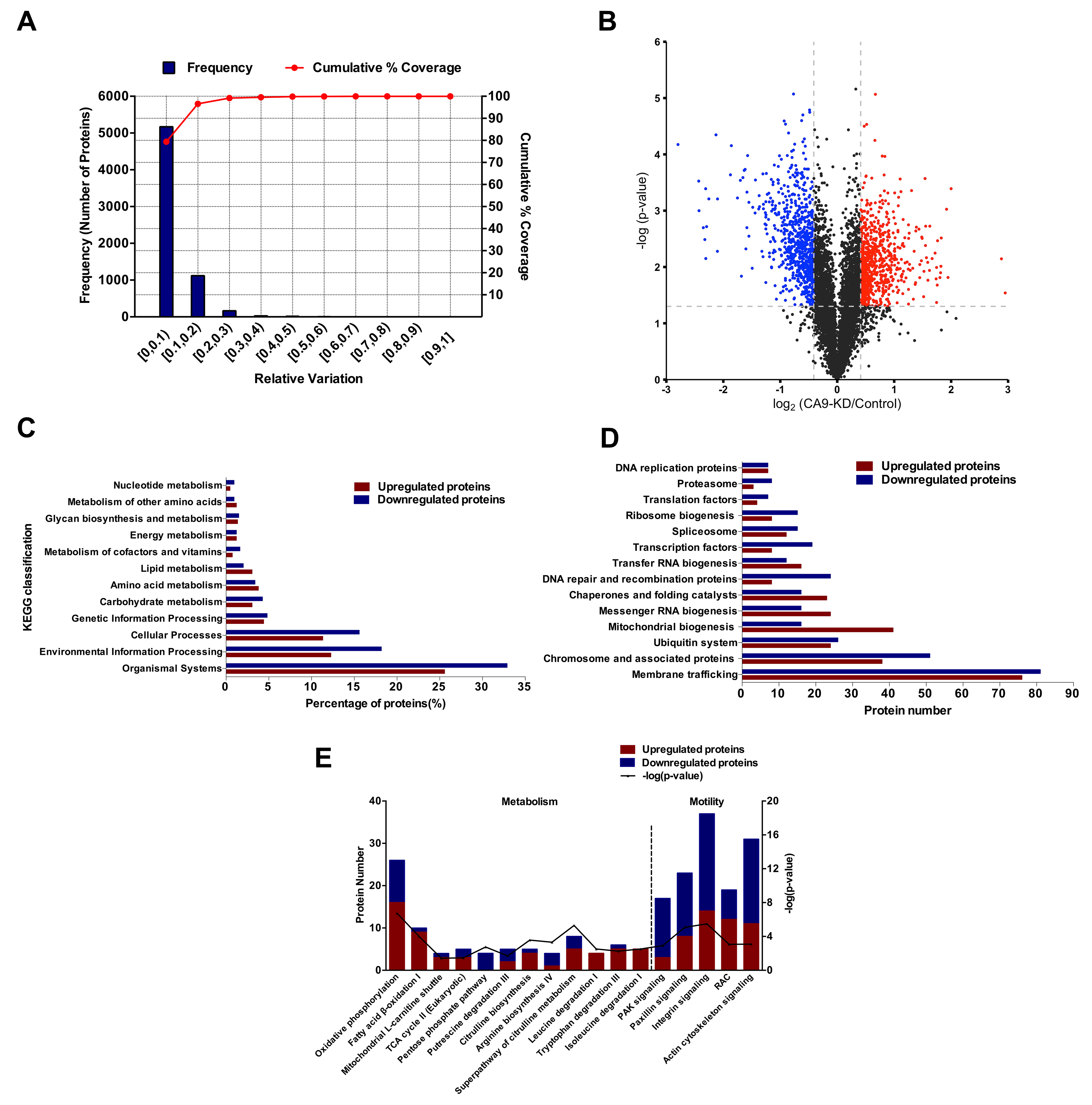

Supplement: Supplementary file 1 [file ijms-21-05939-s001.zip › Supplementary Files/Figures/Figure 2.tif]

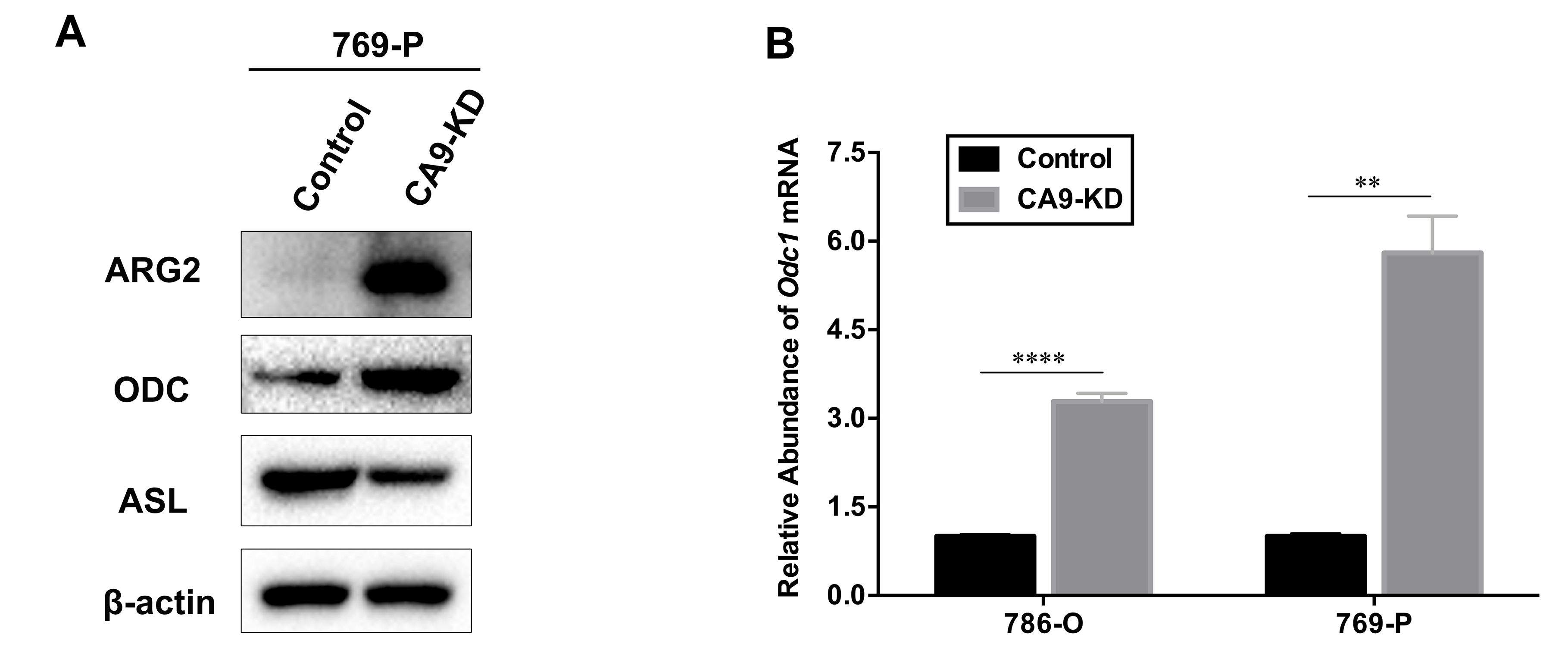

Supplement: Supplementary file 1 [file ijms-21-05939-s001.zip › Supplementary Files/Figures/Figure S4.tif]

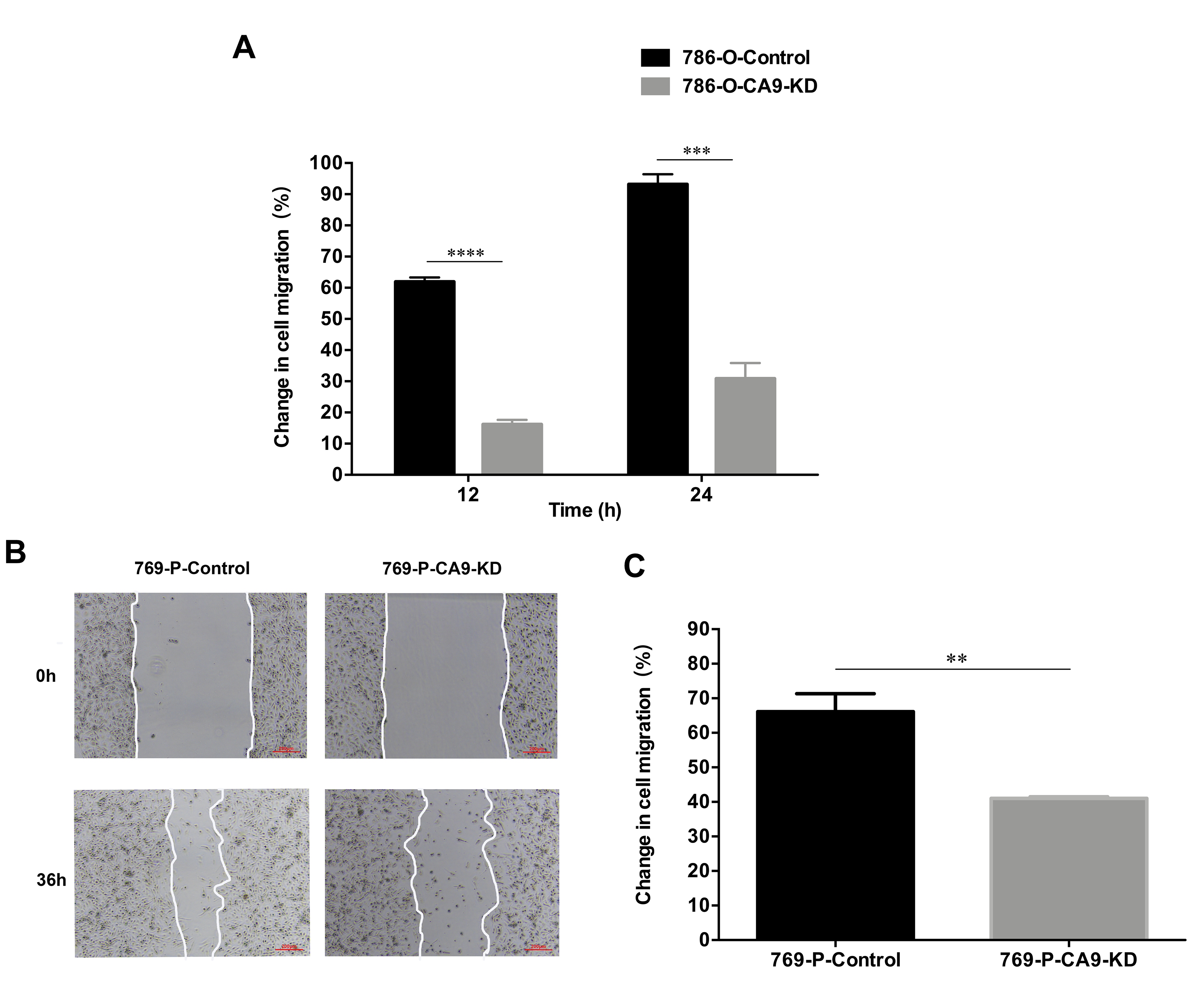

Supplement: Supplementary file 1 [file ijms-21-05939-s001.zip › Supplementary Files/Figures/Figure S5.tif]

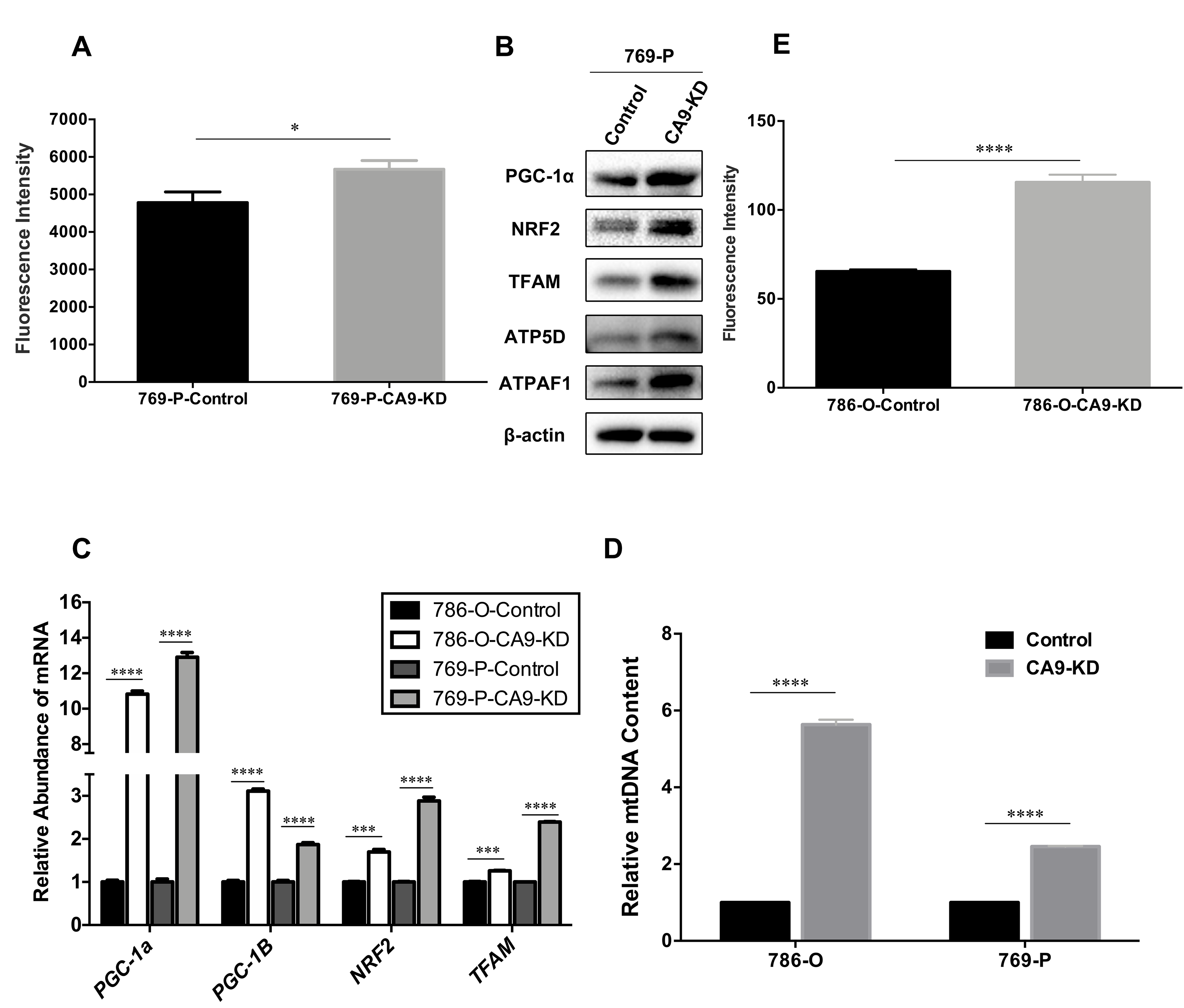

Supplement: Supplementary file 1 [file ijms-21-05939-s001.zip › Supplementary Files/Figures/Figure S2.tif]

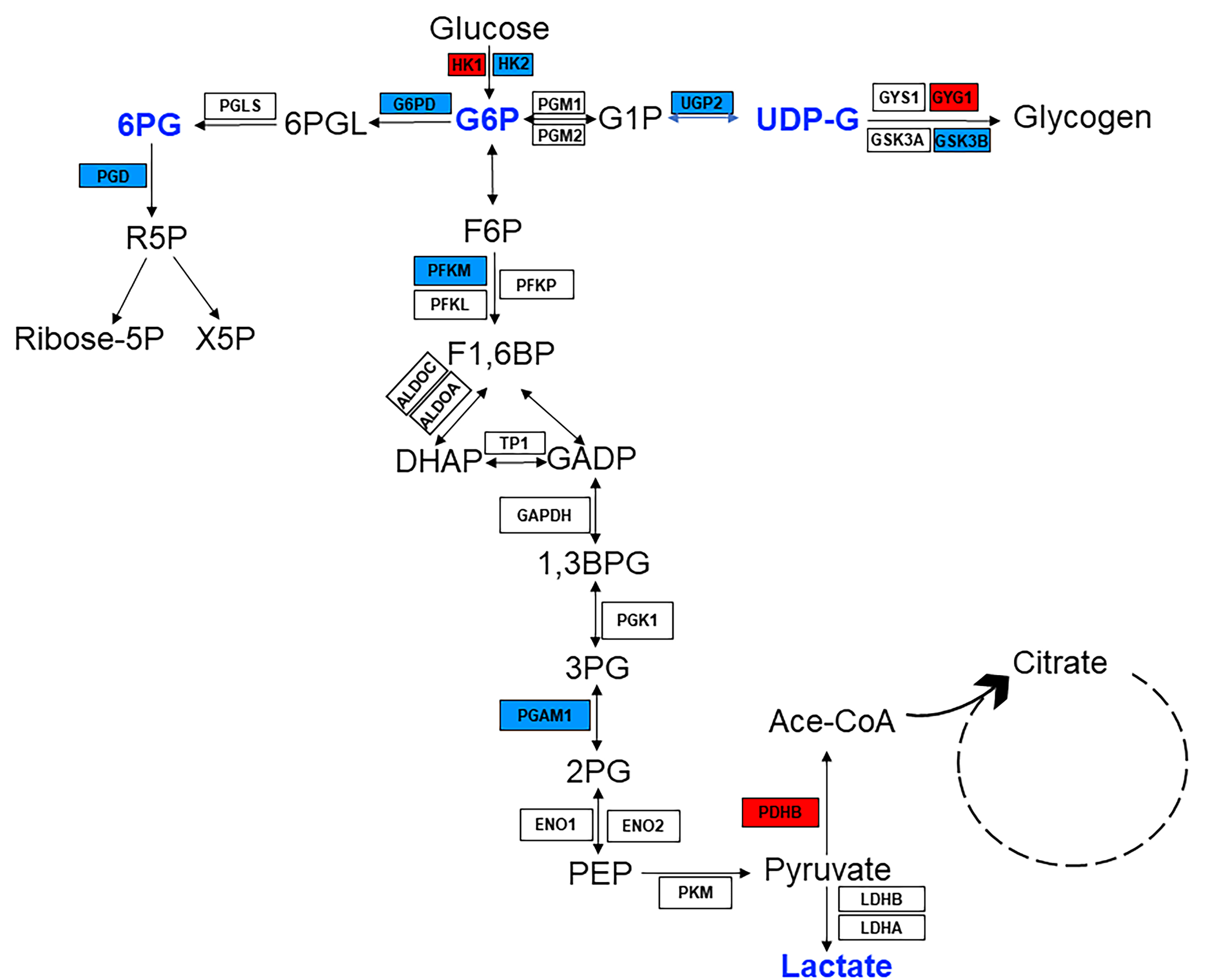

Supplement: Supplementary file 1 [file ijms-21-05939-s001.zip › Supplementary Files/Figures/Figure S3.tif]

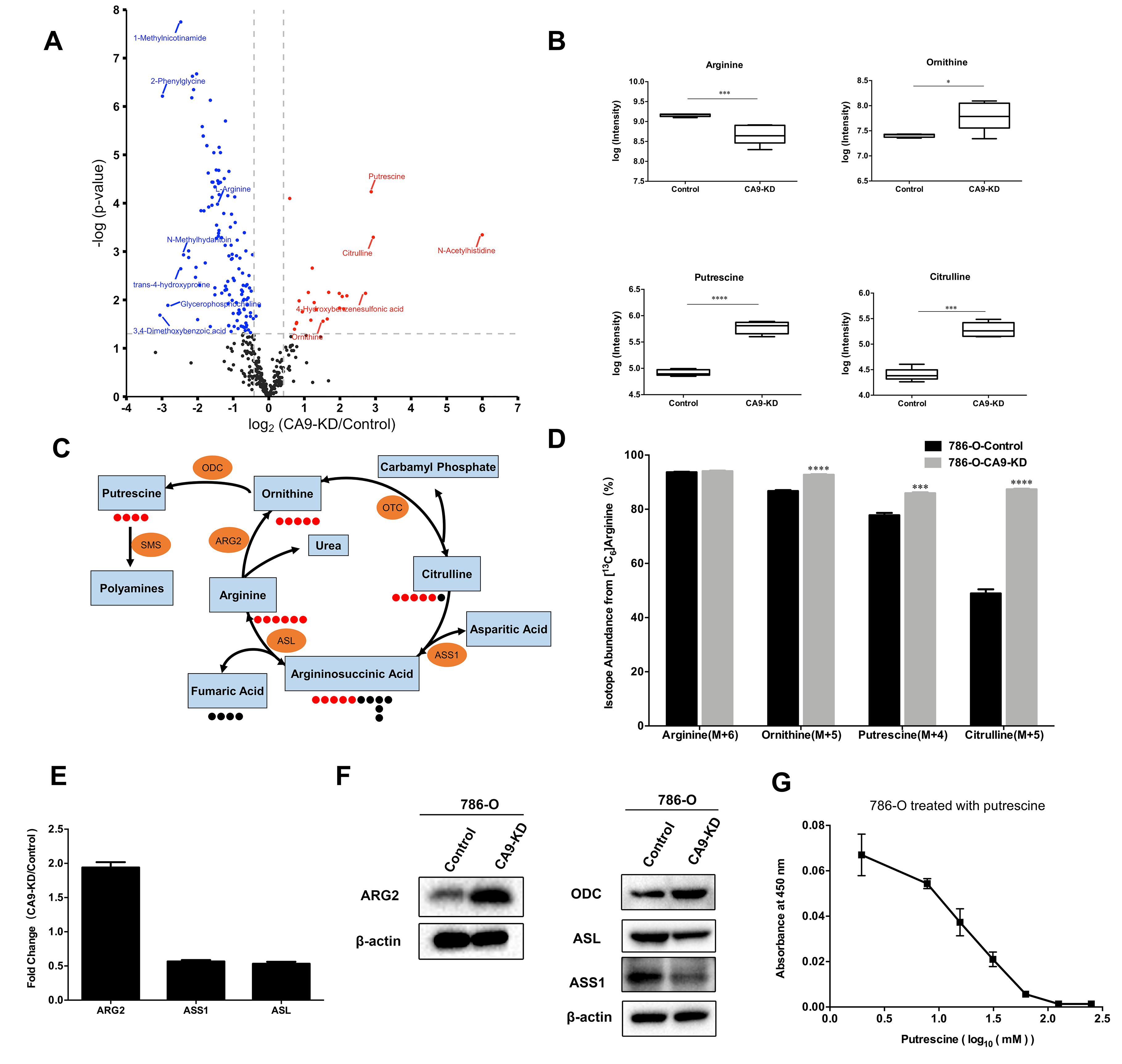

Supplement: Supplementary file 1 [file ijms-21-05939-s001.zip › Supplementary Files/Figures/Figure 4 new.tif]

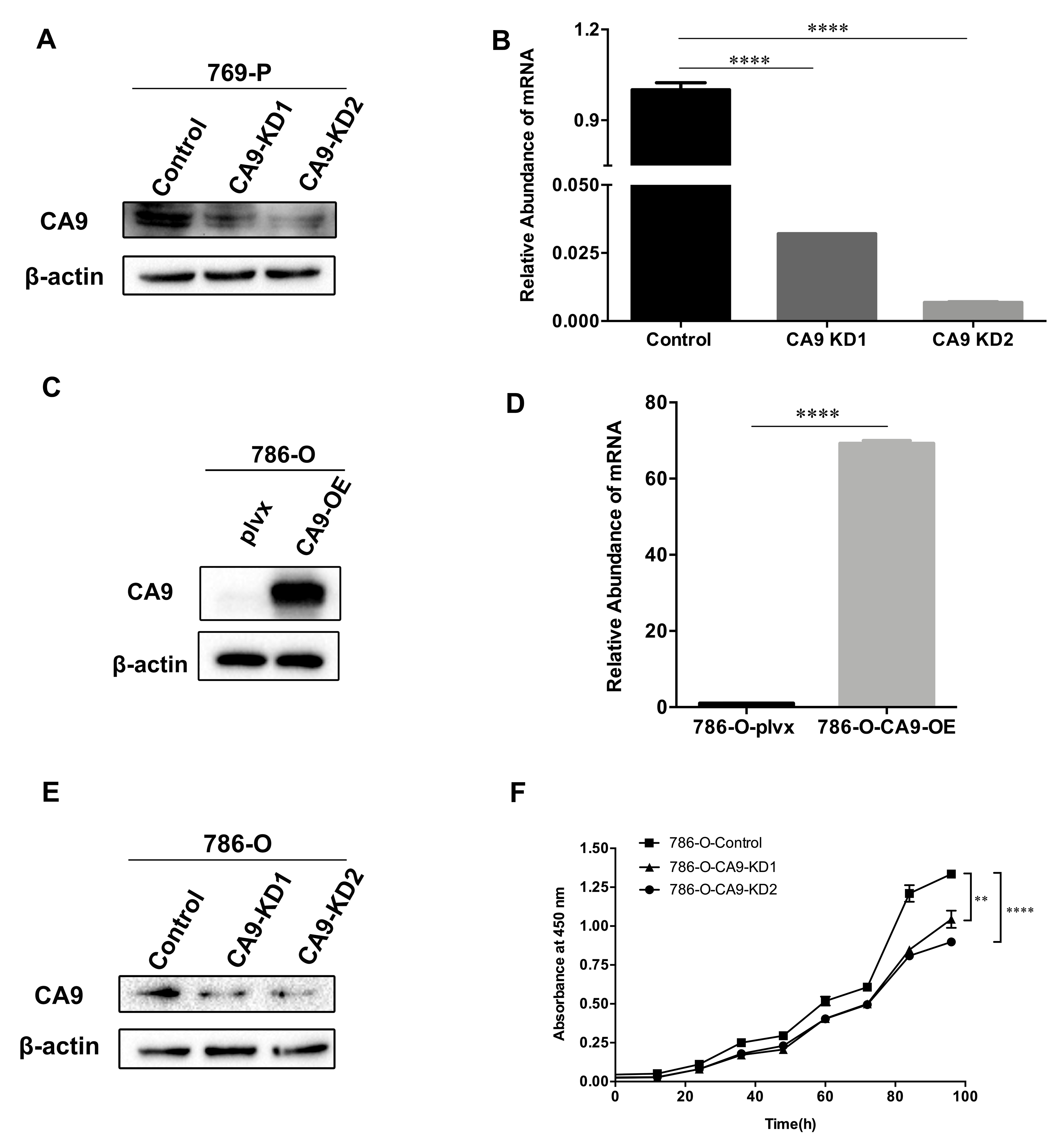

Supplement: Supplementary file 1 [file ijms-21-05939-s001.zip › Supplementary Files/Figures/Figure S1.tif]

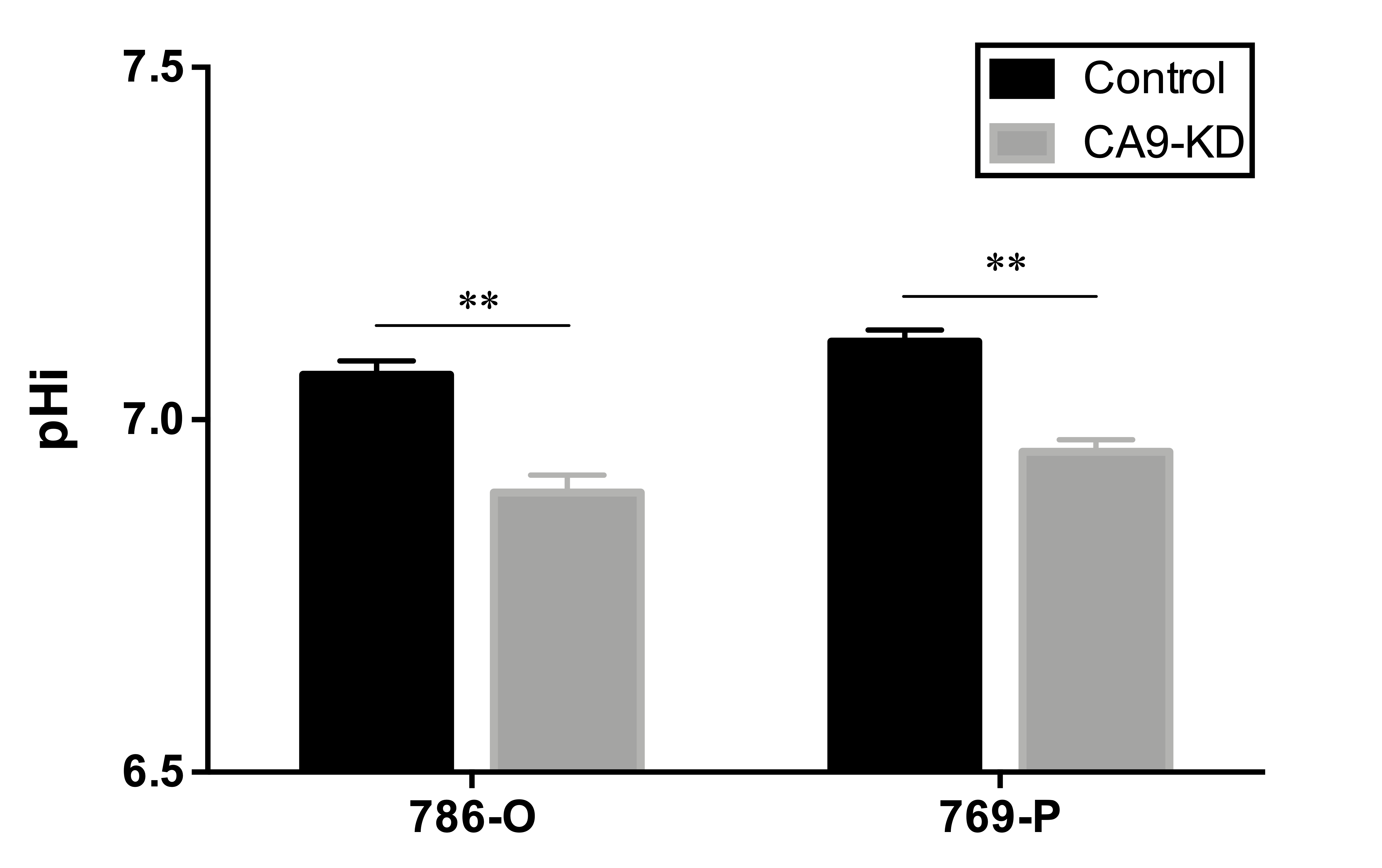

Supplement: Supplementary file 1 [file ijms-21-05939-s001.zip › Supplementary Files/Figures/Figure S6.tiff]

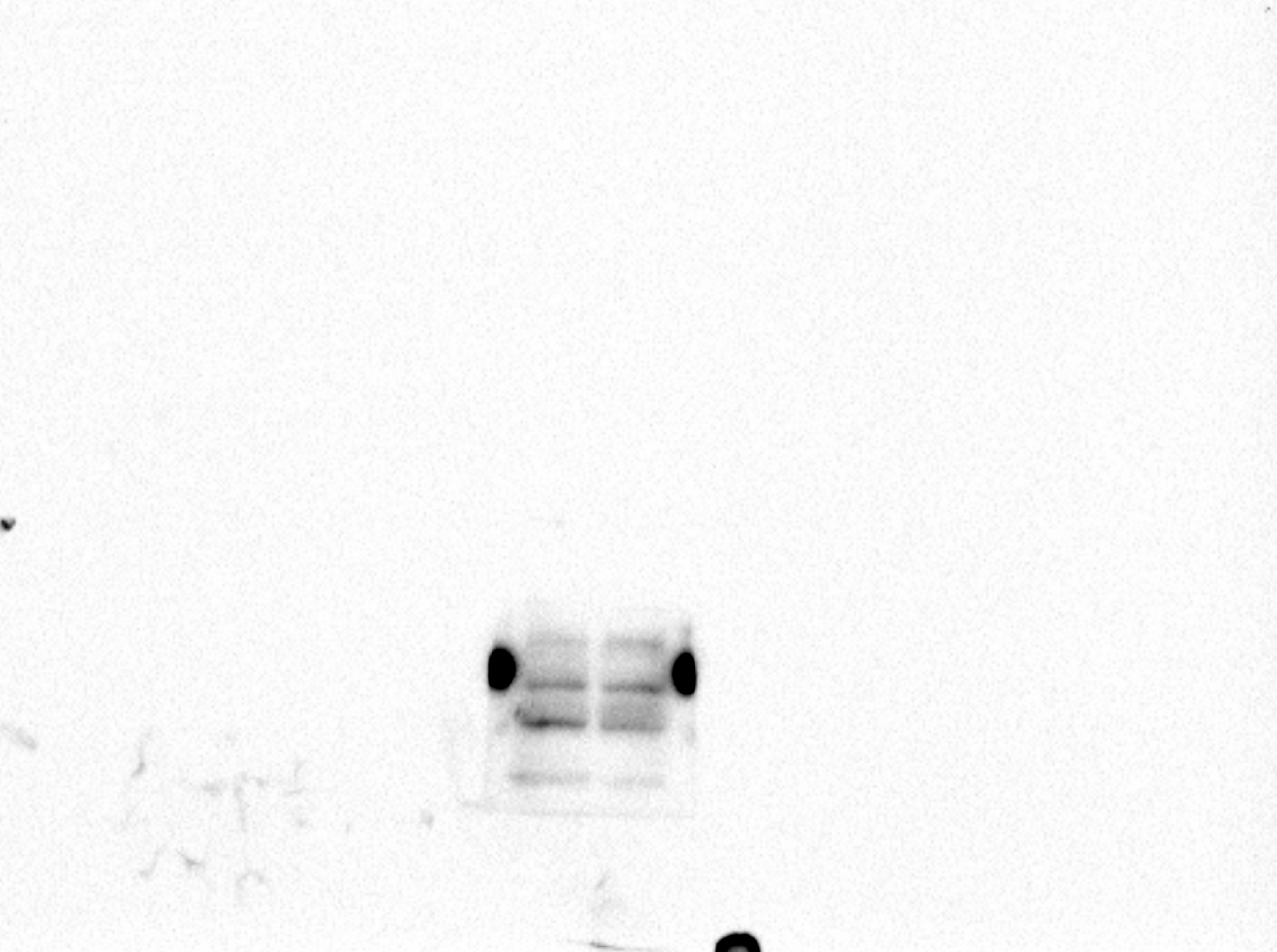

Supplement: Supplementary file 1 [file ijms-21-05939-s001.zip › Supplementary Files/Original images of western blots/Fig 5D-p70S6K.tif]

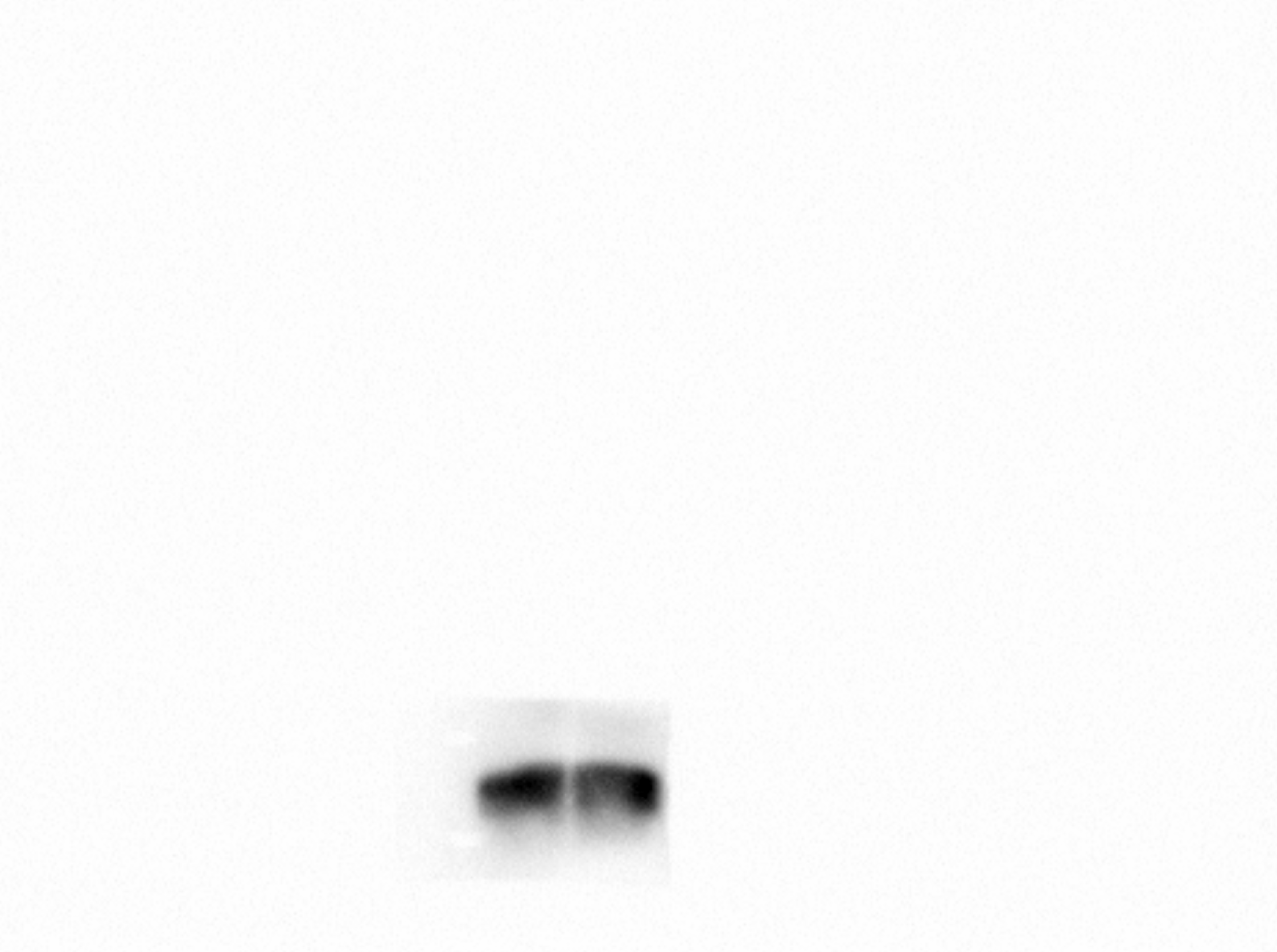

Supplement: Supplementary file 1 [file ijms-21-05939-s001.zip › Supplementary Files/Original images of western blots/Fig 5D-4EBP1.tif]

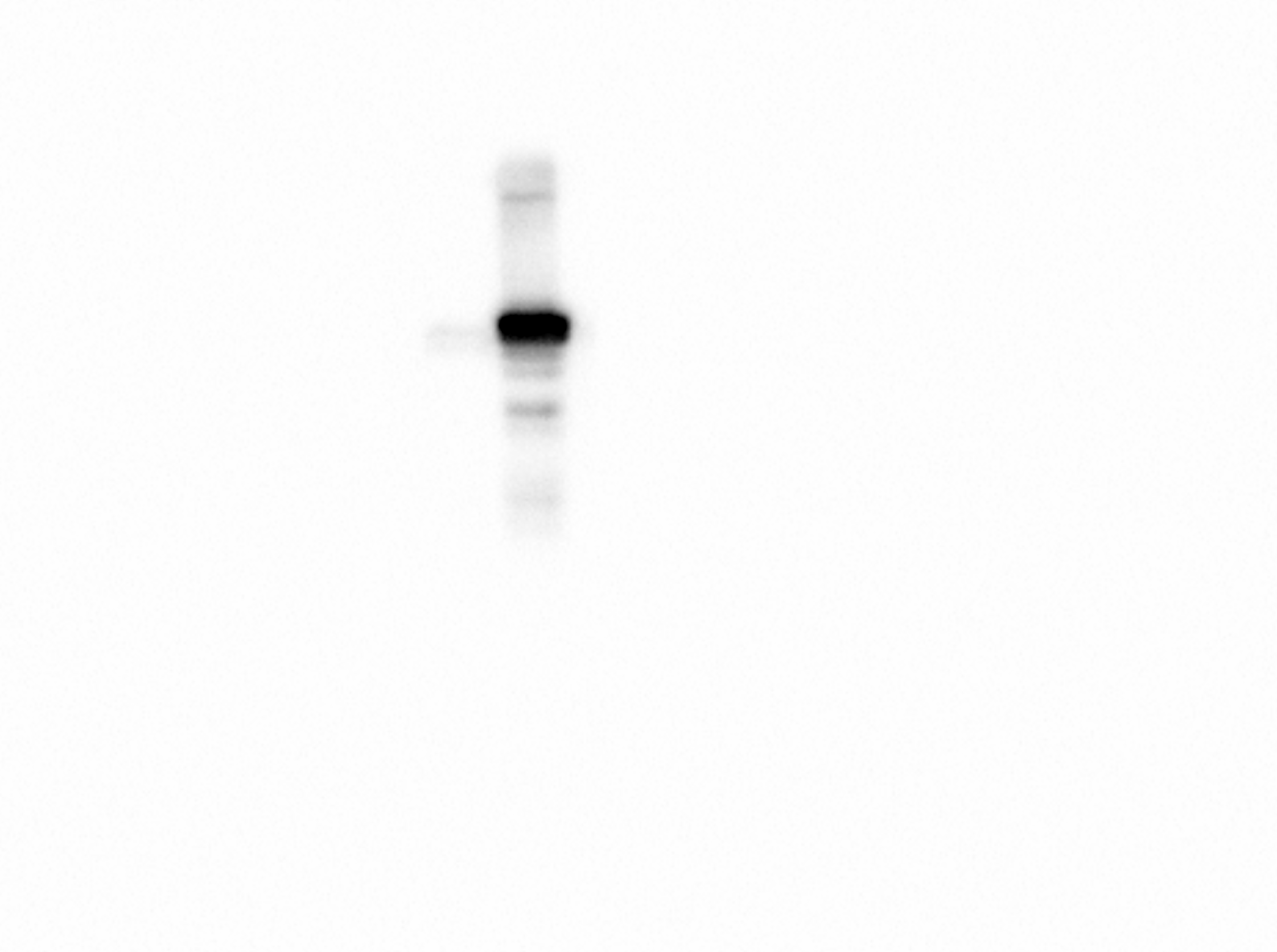

Supplement: Supplementary file 1 [file ijms-21-05939-s001.zip › Supplementary Files/Original images of western blots/Supplementary Figure S1C-CA9.tif]

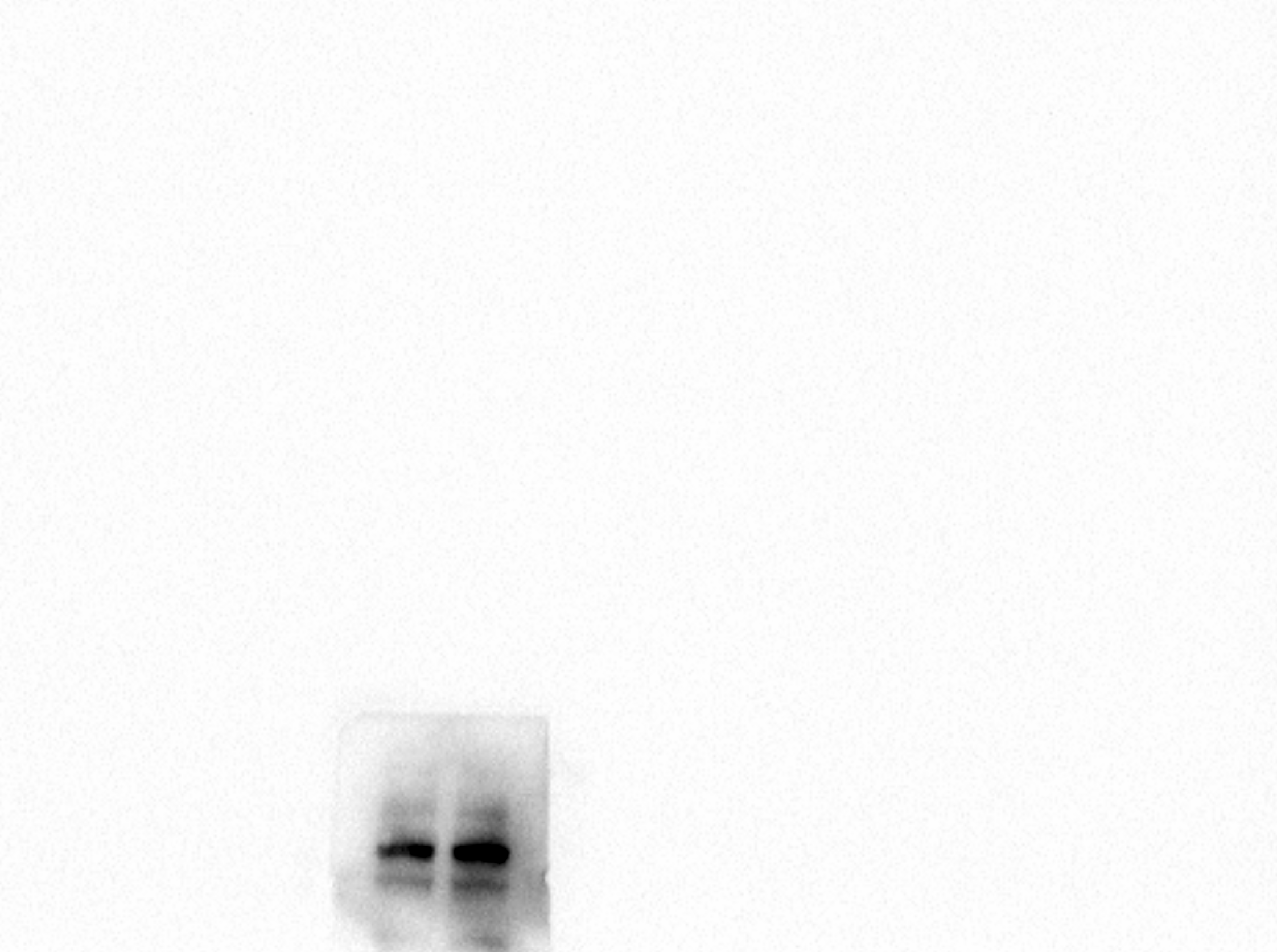

Supplement: Supplementary file 1 [file ijms-21-05939-s001.zip › Supplementary Files/Original images of western blots/Fig 3D-PGC-1╬▒.tif]

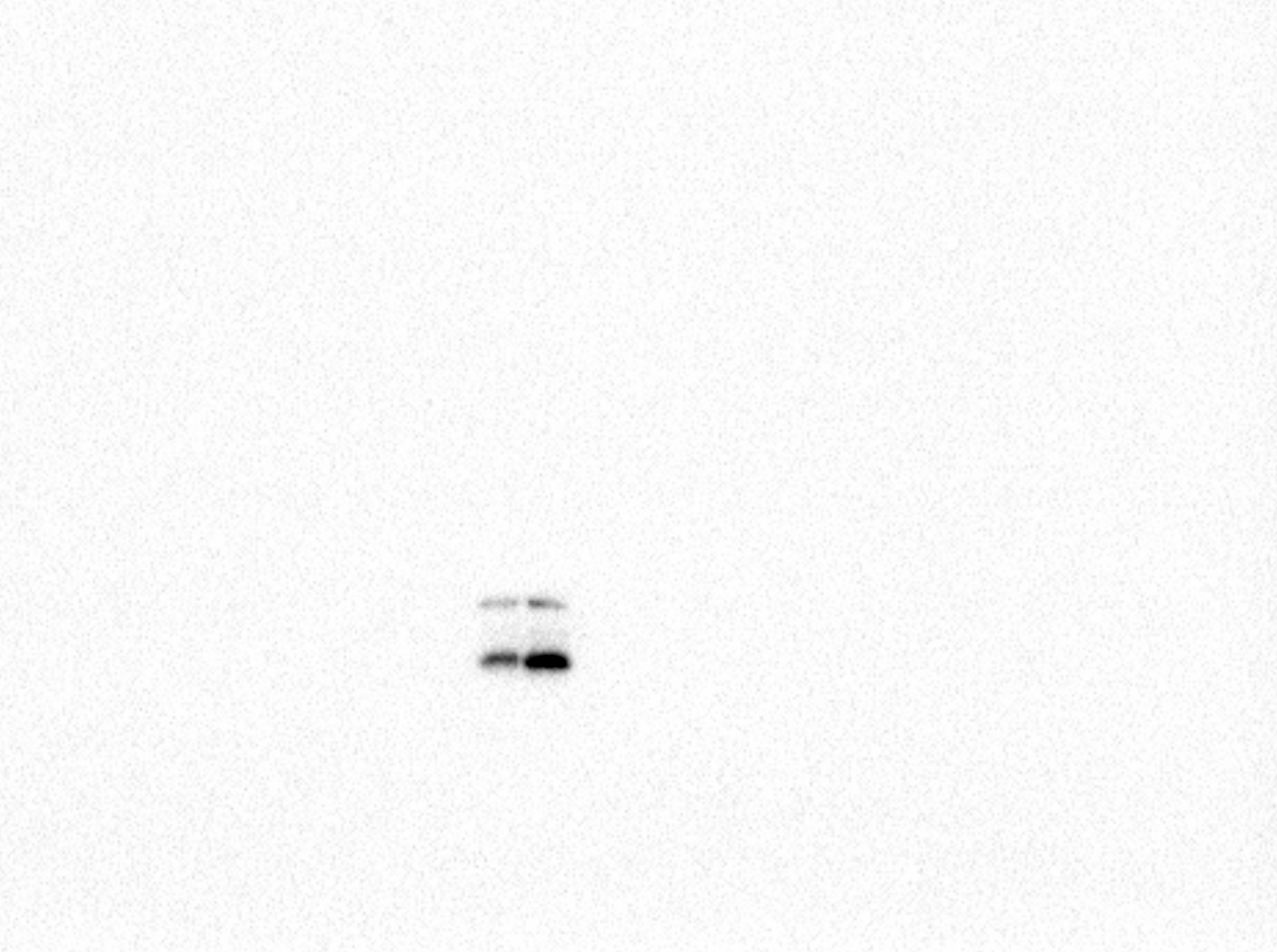

Supplement: Supplementary file 1 [file ijms-21-05939-s001.zip › Supplementary Files/Original images of western blots/Fig 4F-ARG2.tif]

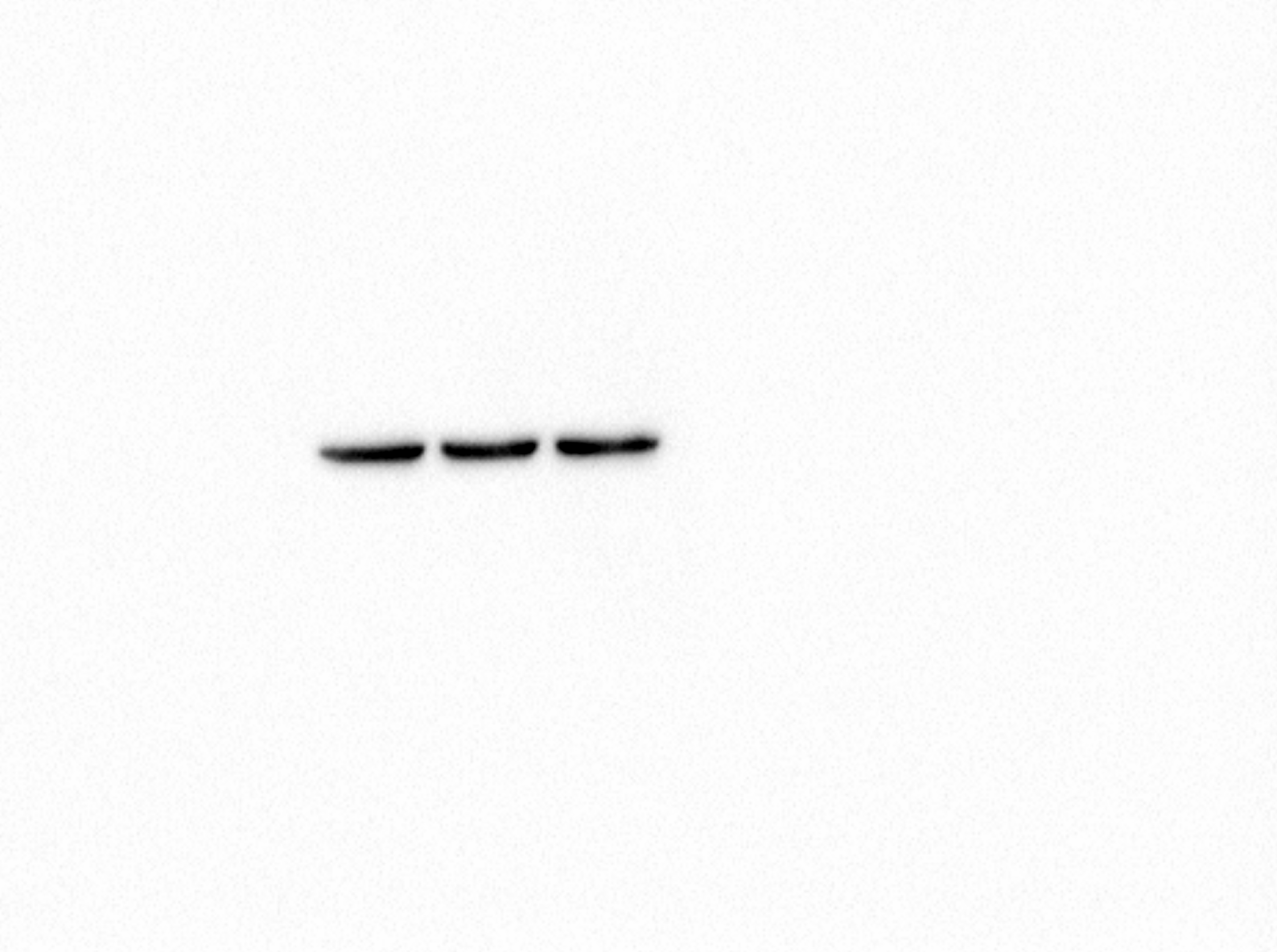

Supplement: Supplementary file 1 [file ijms-21-05939-s001.zip › Supplementary Files/Original images of western blots/Supplementary Figure S1A-╬▓-actin.tif]

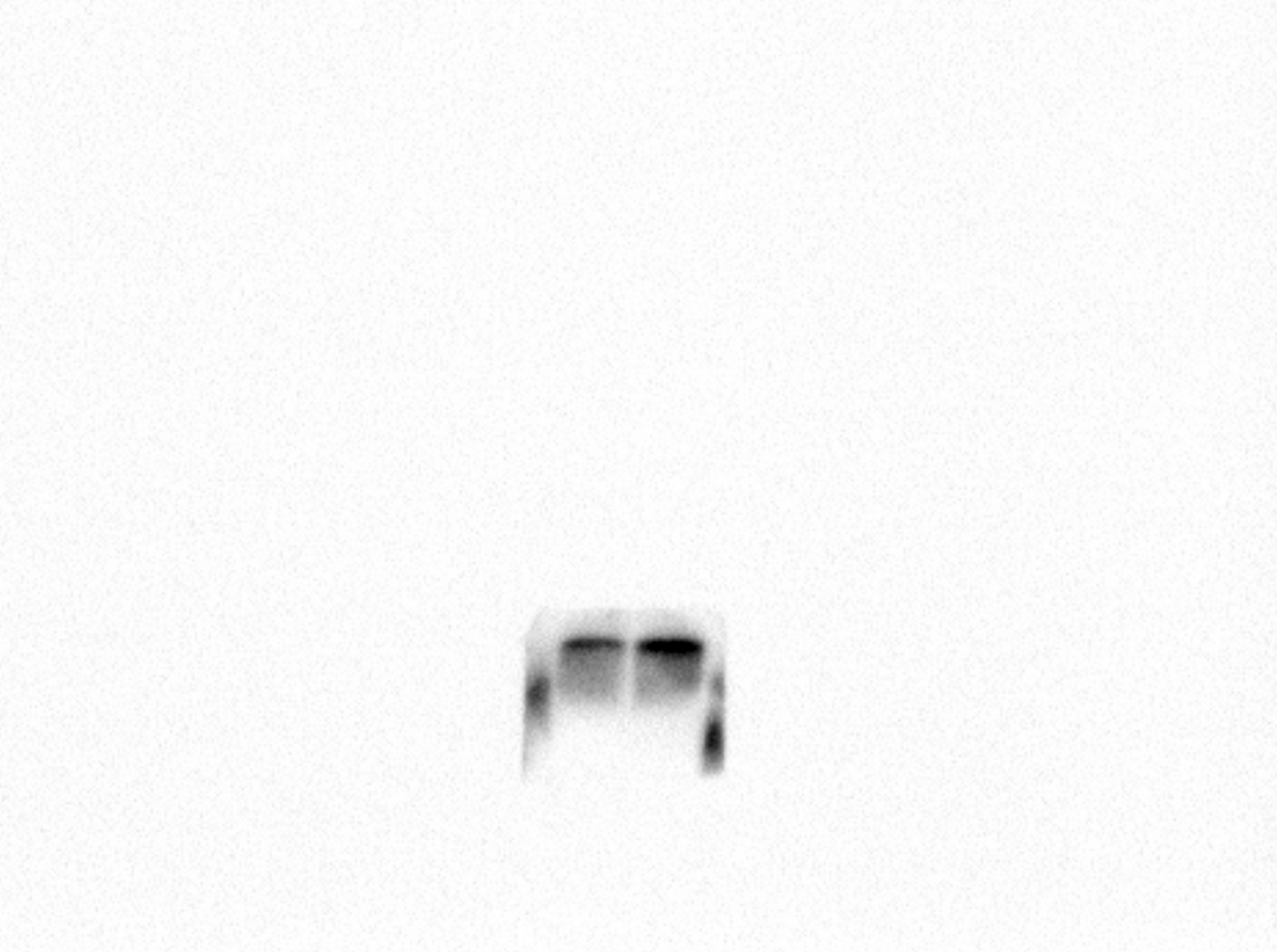

Supplement: Supplementary file 1 [file ijms-21-05939-s001.zip › Supplementary Files/Original images of western blots/Fig 3D-ATP5D.tif]

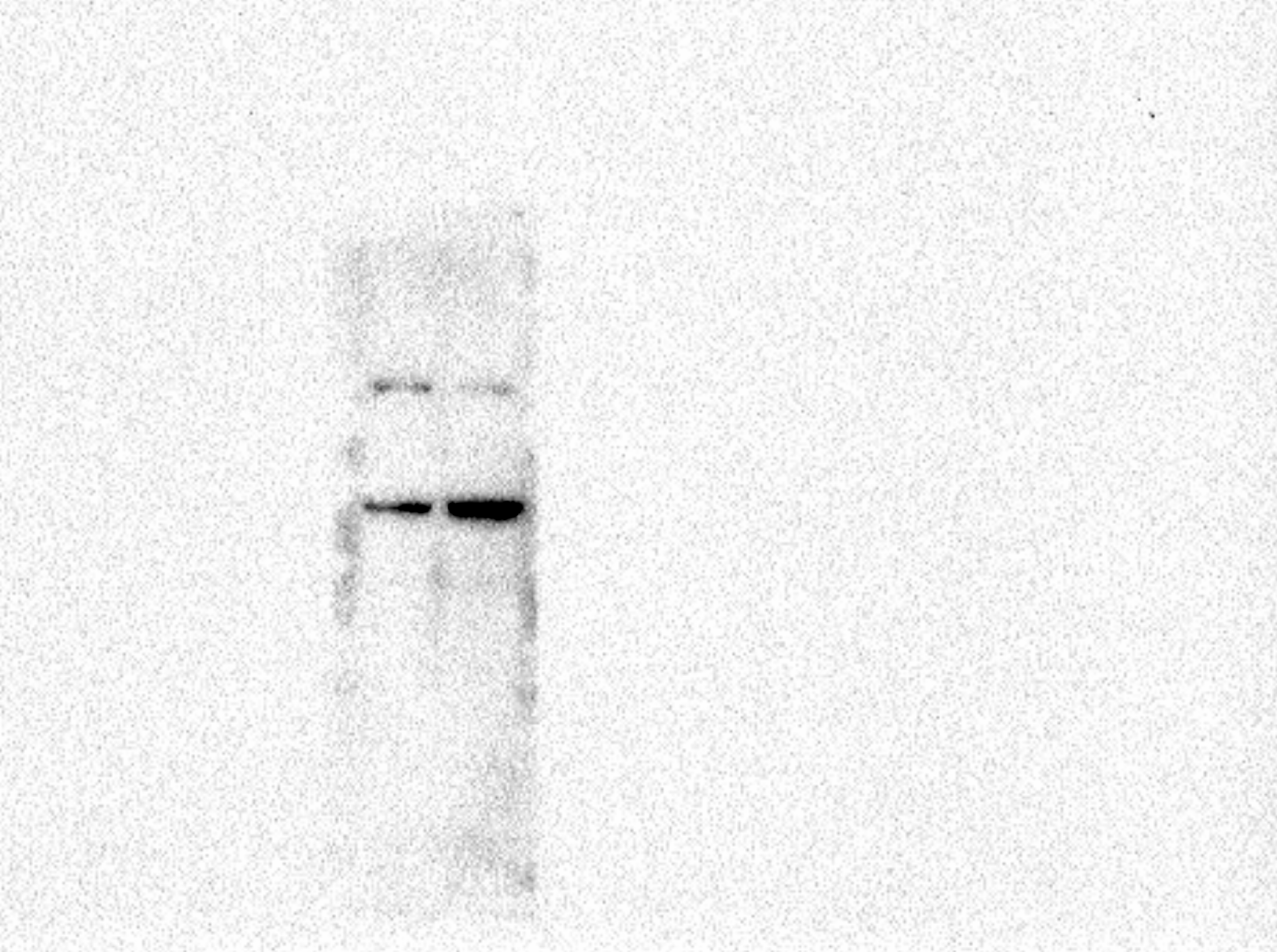

Supplement: Supplementary file 1 [file ijms-21-05939-s001.zip › Supplementary Files/Original images of western blots/Supplementary Figure S4A-ODC.tif]

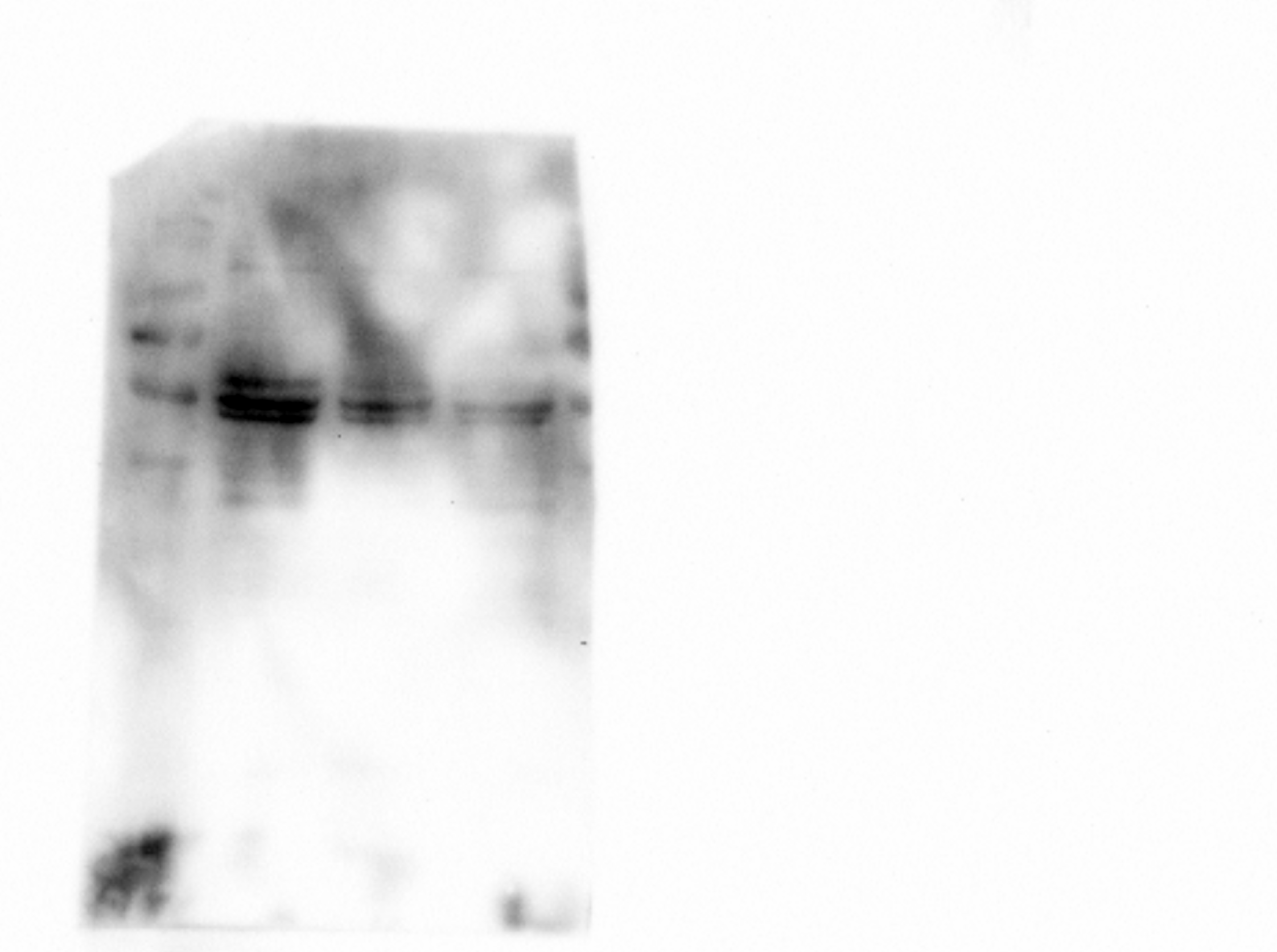

Supplement: Supplementary file 1 [file ijms-21-05939-s001.zip › Supplementary Files/Original images of western blots/Supplementary Figure S1A-CA9.tif]

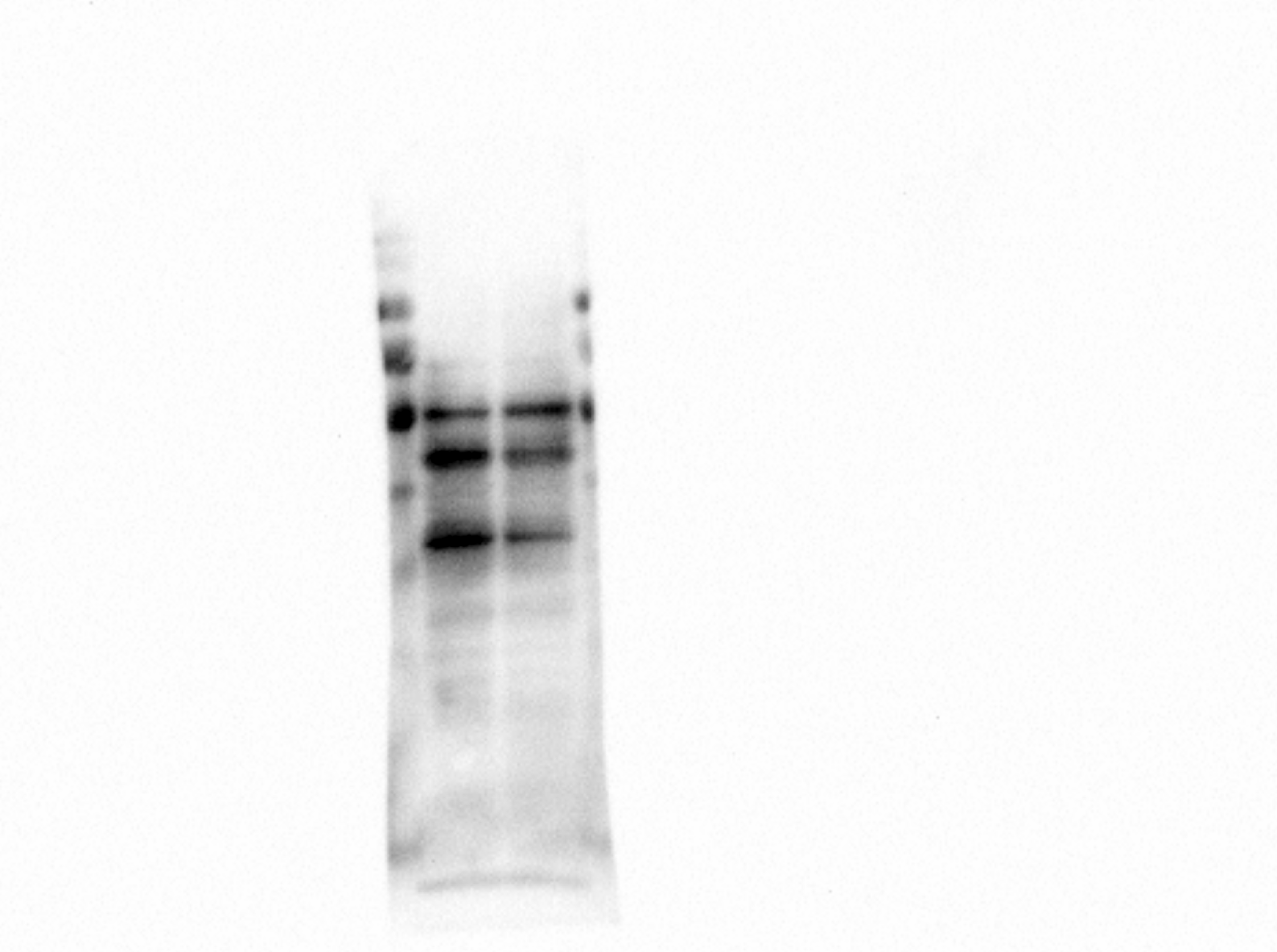

Supplement: Supplementary file 1 [file ijms-21-05939-s001.zip › Supplementary Files/Original images of western blots/Fig 4F-ASS1.tif]

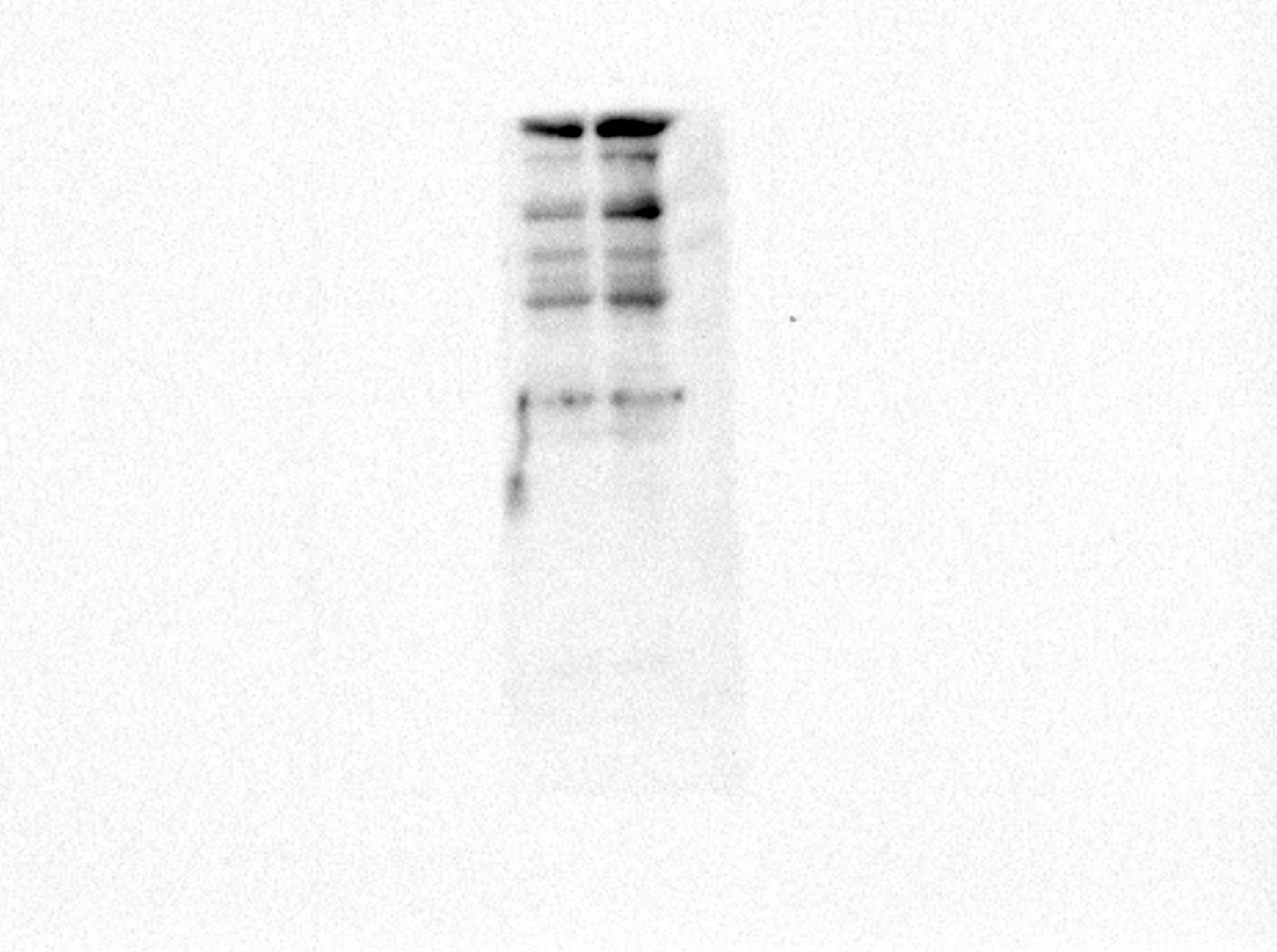

Supplement: Supplementary file 1 [file ijms-21-05939-s001.zip › Supplementary Files/Original images of western blots/Supplementary Figure S2B-PGC1-╬▒.tif]

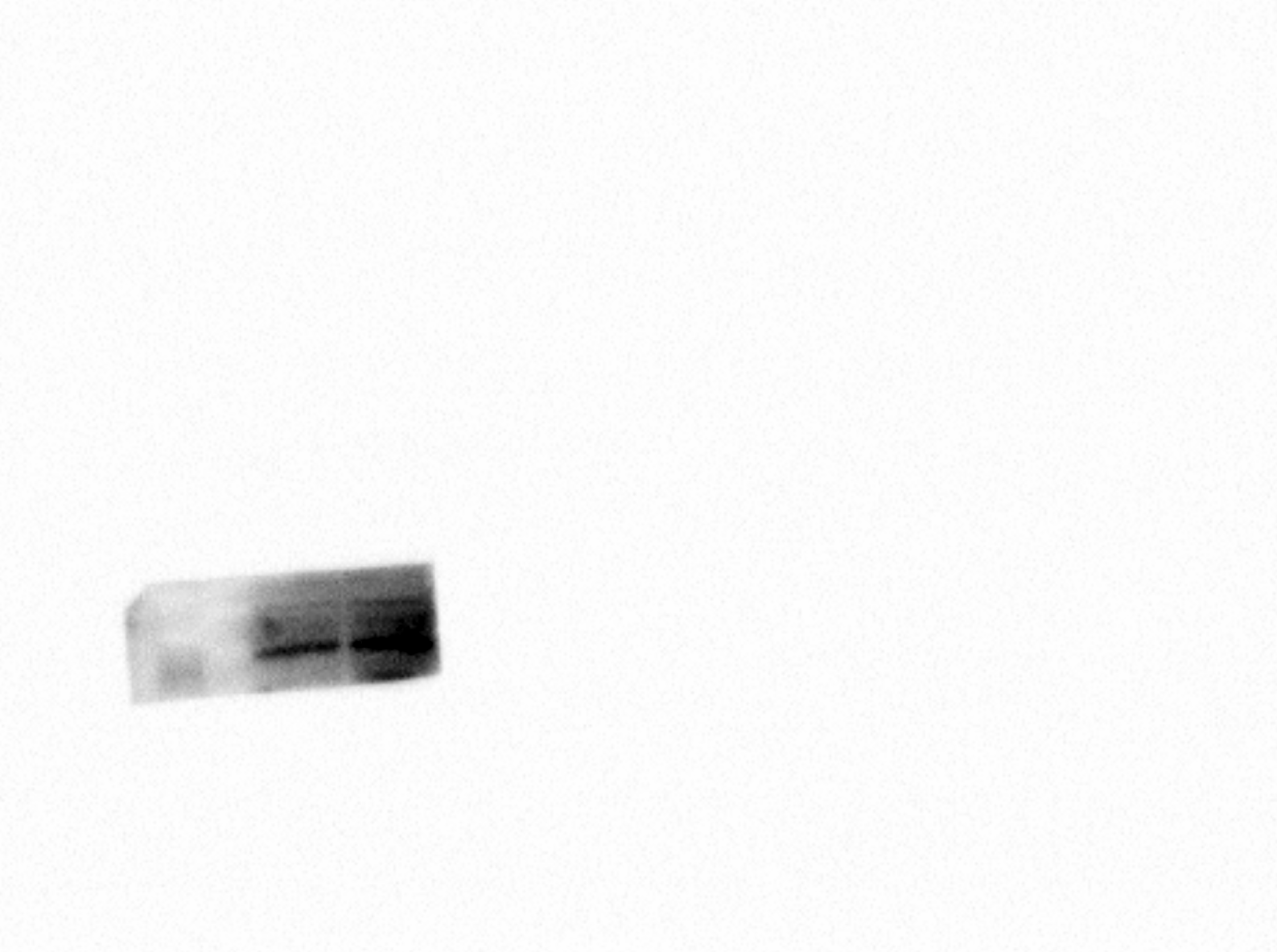

Supplement: Supplementary file 1 [file ijms-21-05939-s001.zip › Supplementary Files/Original images of western blots/Fig 3D-ATPAF1.tif]

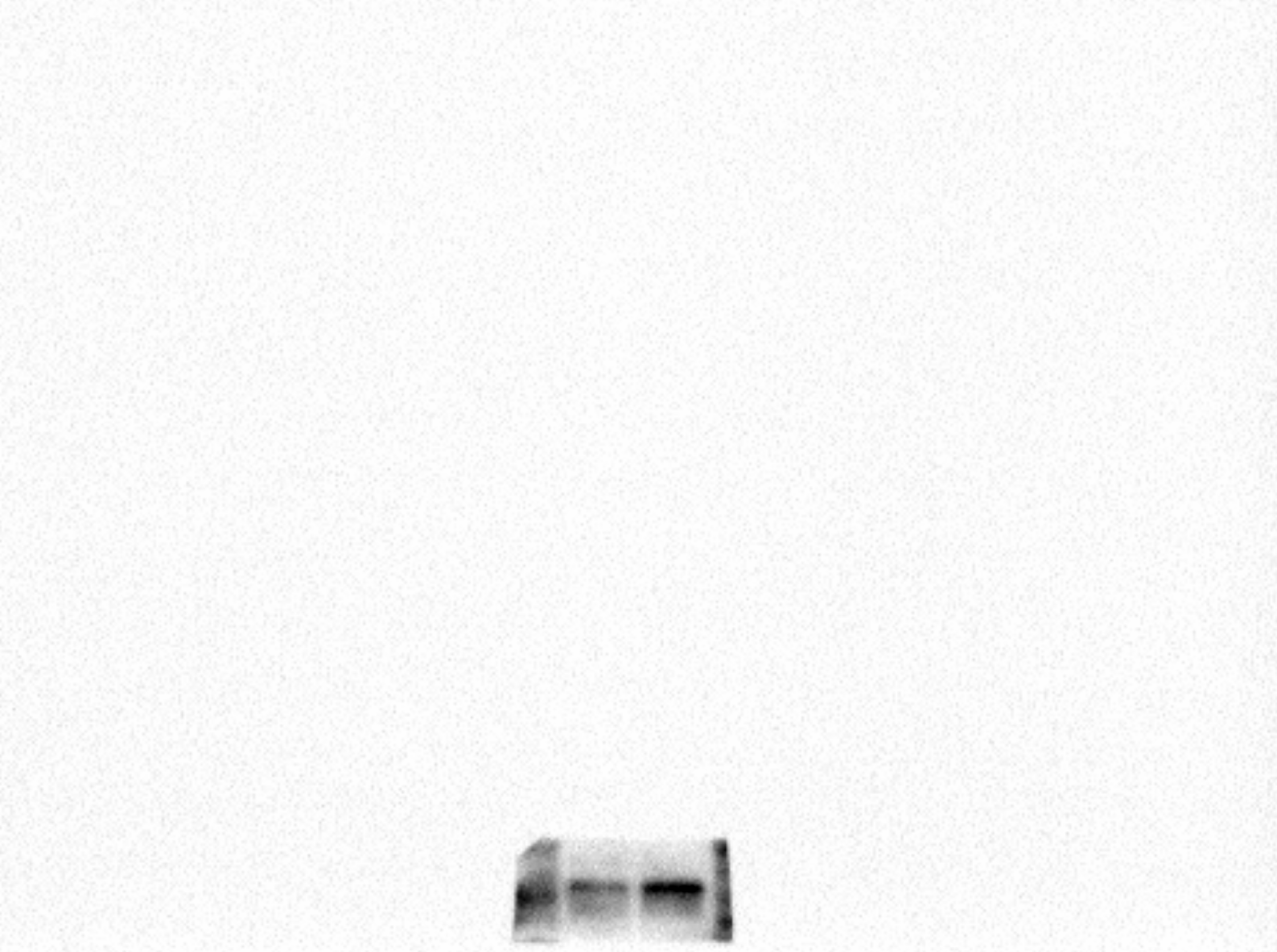

Supplement: Supplementary file 1 [file ijms-21-05939-s001.zip › Supplementary Files/Original images of western blots/Fig 3D-TFAM.tif]

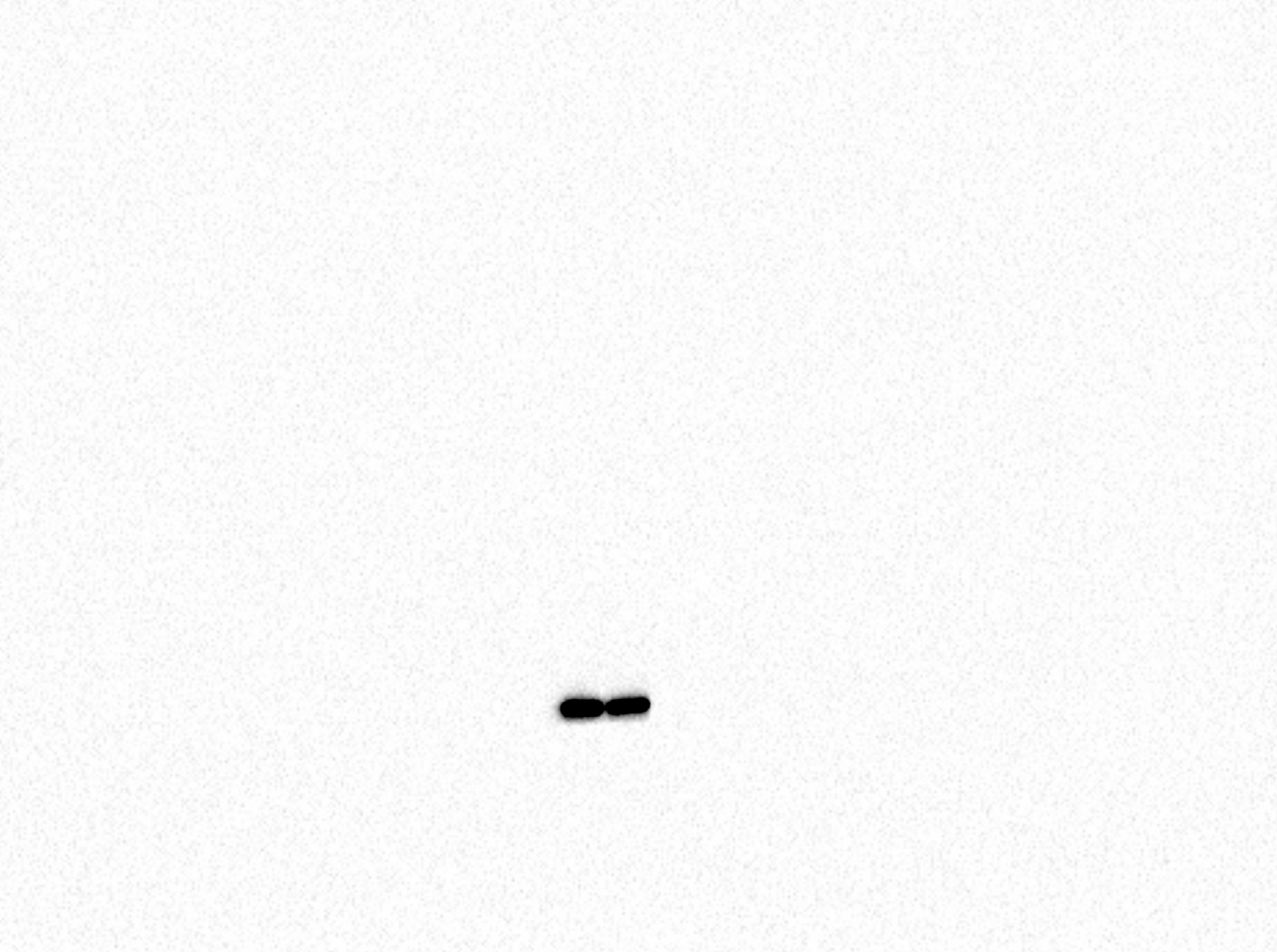

Supplement: Supplementary file 1 [file ijms-21-05939-s001.zip › Supplementary Files/Original images of western blots/Fig 4F-╬▓-actin.tif]

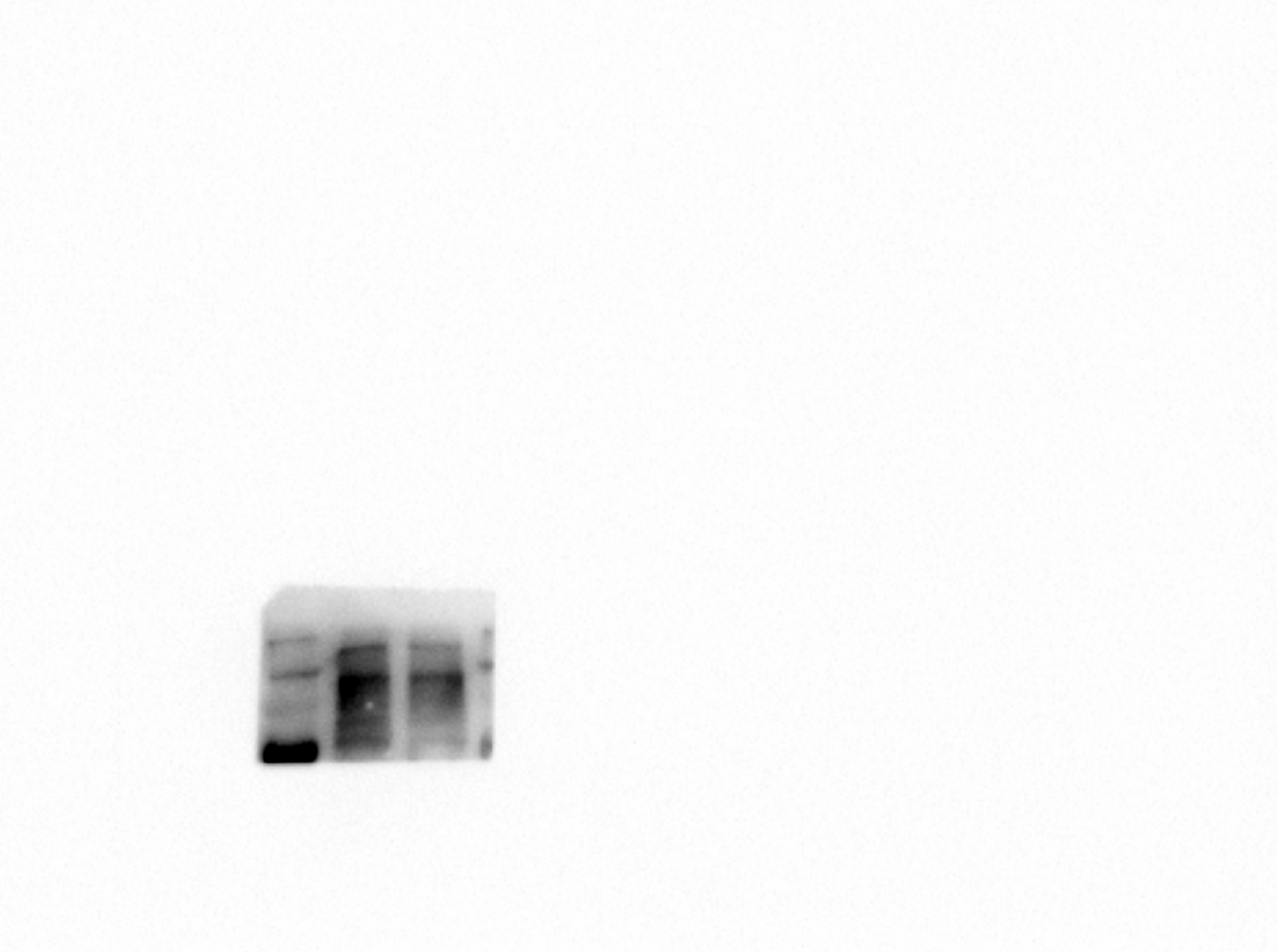

Supplement: Supplementary file 1 [file ijms-21-05939-s001.zip › Supplementary Files/Original images of western blots/Fig 5D-mTOR.tif]

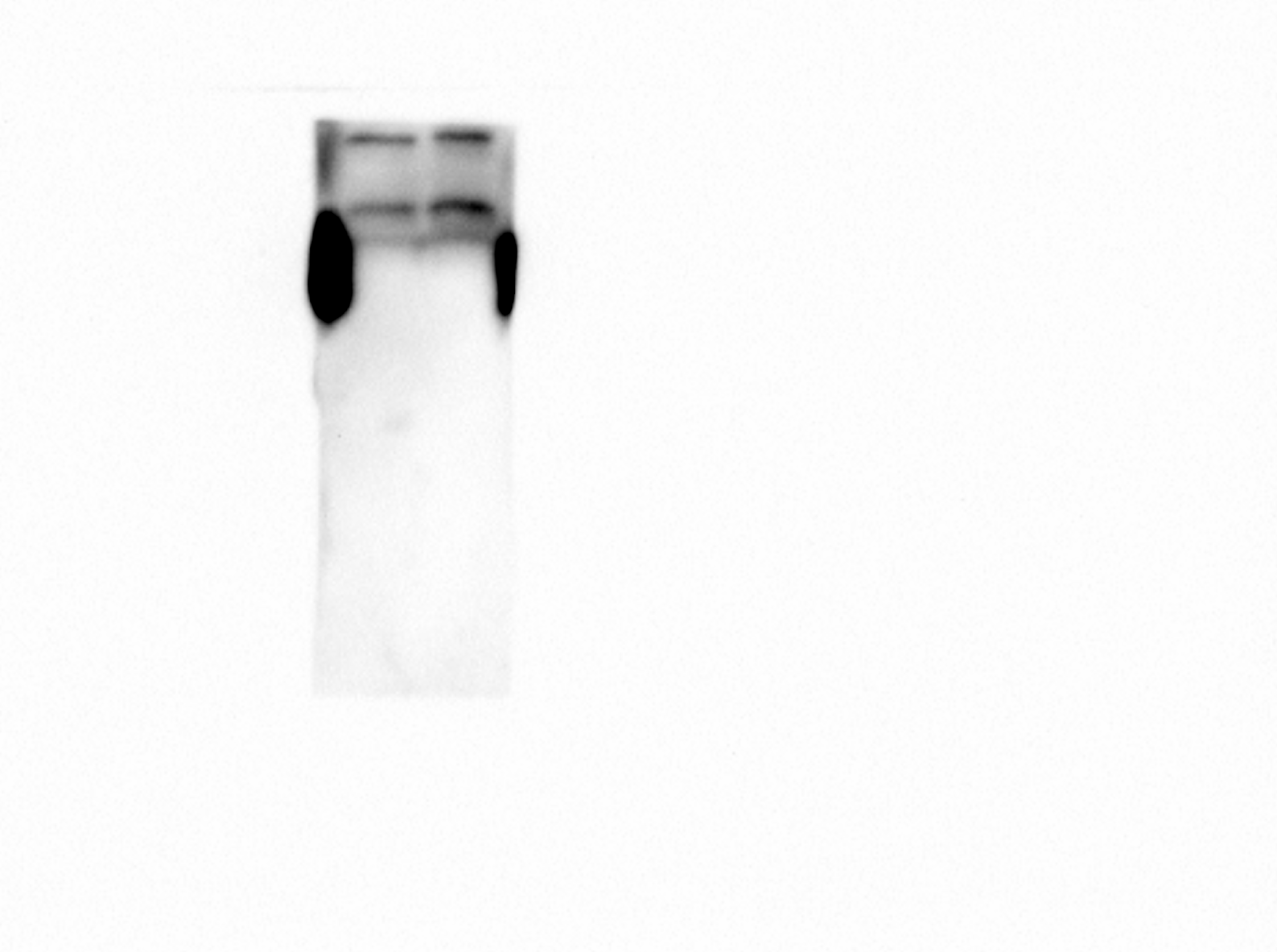

Supplement: Supplementary file 1 [file ijms-21-05939-s001.zip › Supplementary Files/Original images of western blots/Supplementary Figure S2B-ATP5D.tif]

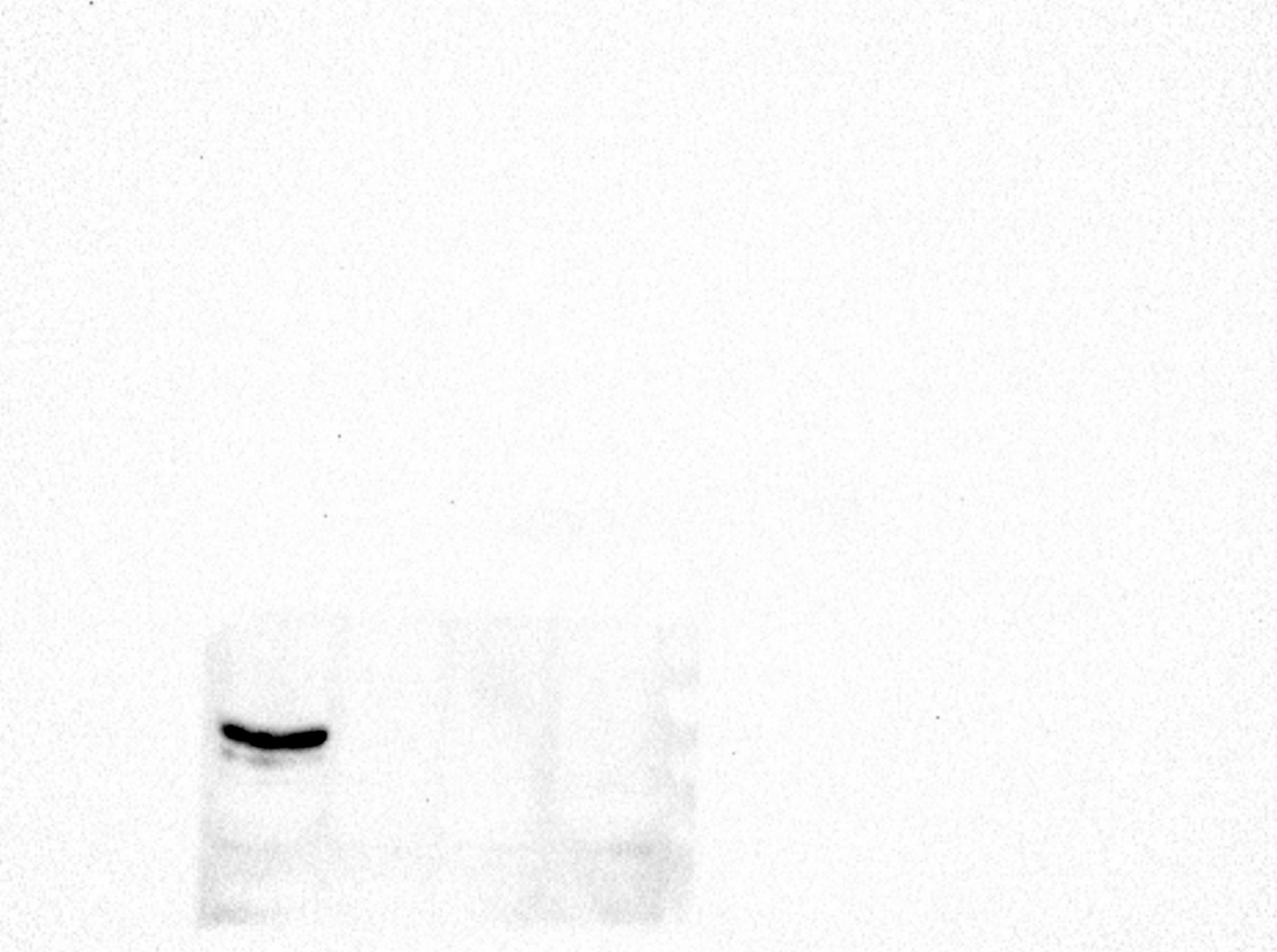

Supplement: Supplementary file 1 [file ijms-21-05939-s001.zip › Supplementary Files/Original images of western blots/Fig 1D-CA9.tif]

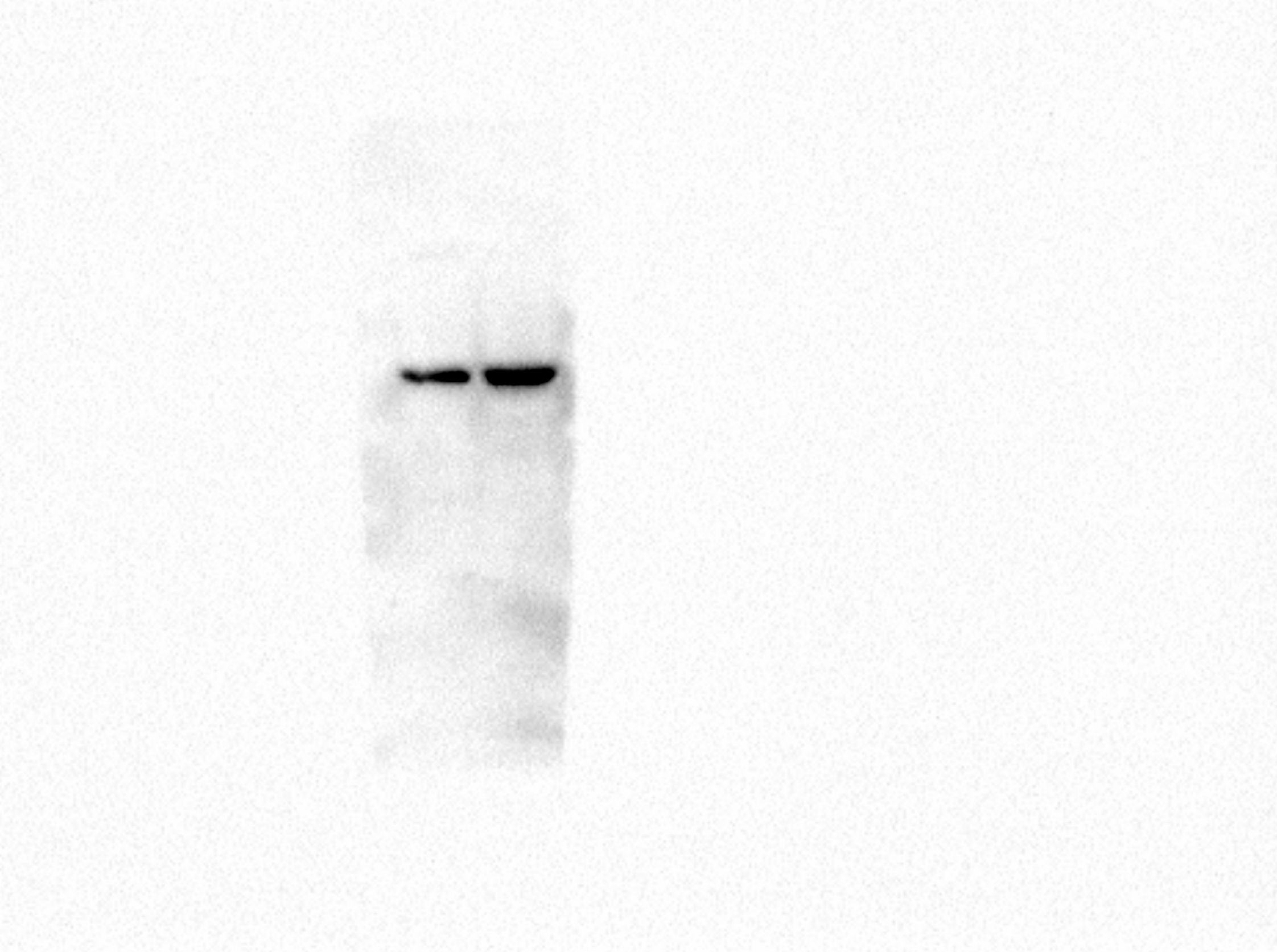

Supplement: Supplementary file 1 [file ijms-21-05939-s001.zip › Supplementary Files/Original images of western blots/Fig 4F-ODC.tif]

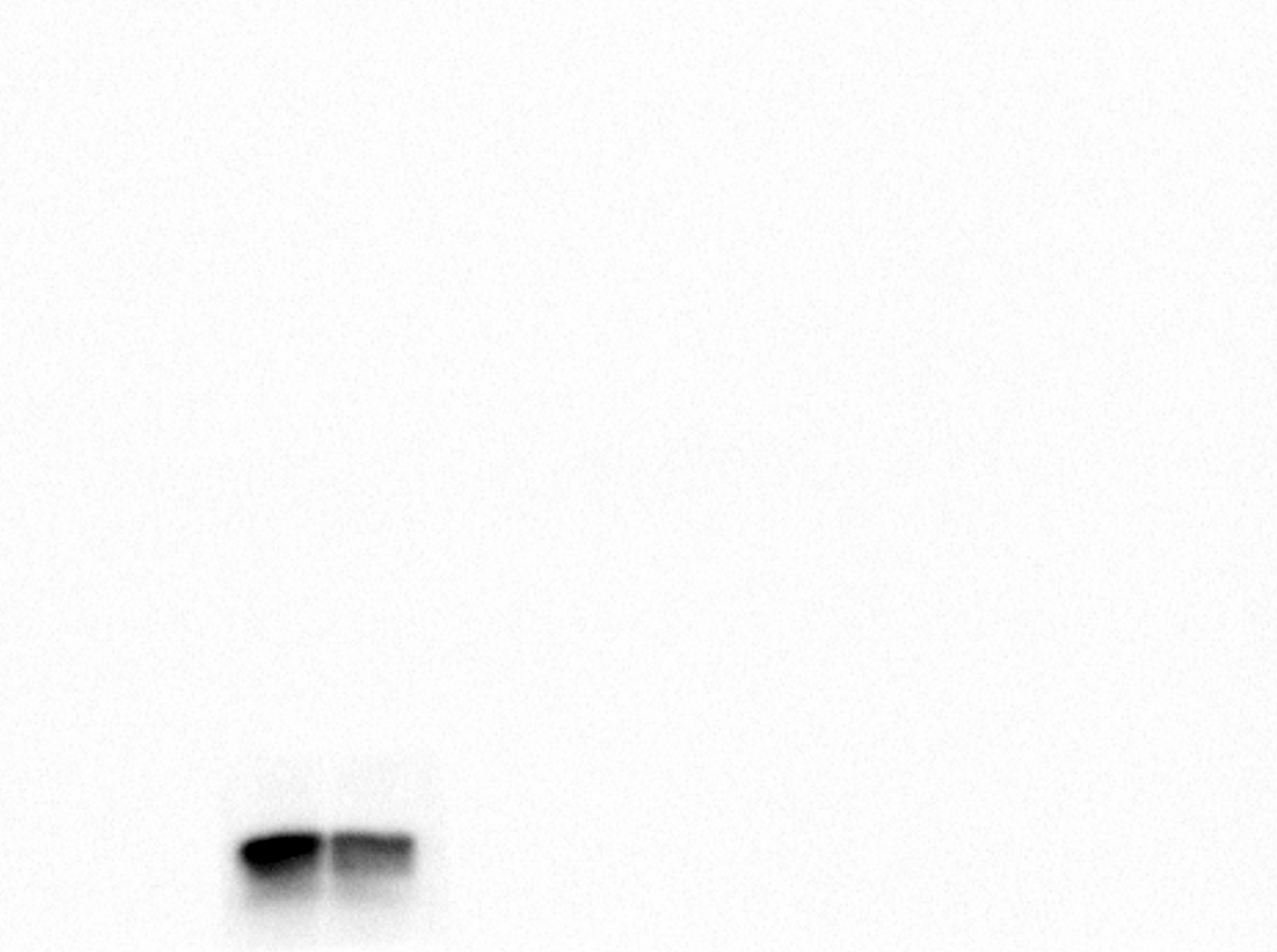

Supplement: Supplementary file 1 [file ijms-21-05939-s001.zip › Supplementary Files/Original images of western blots/Fig 5D-p-4EBP1-T37:46.tif]

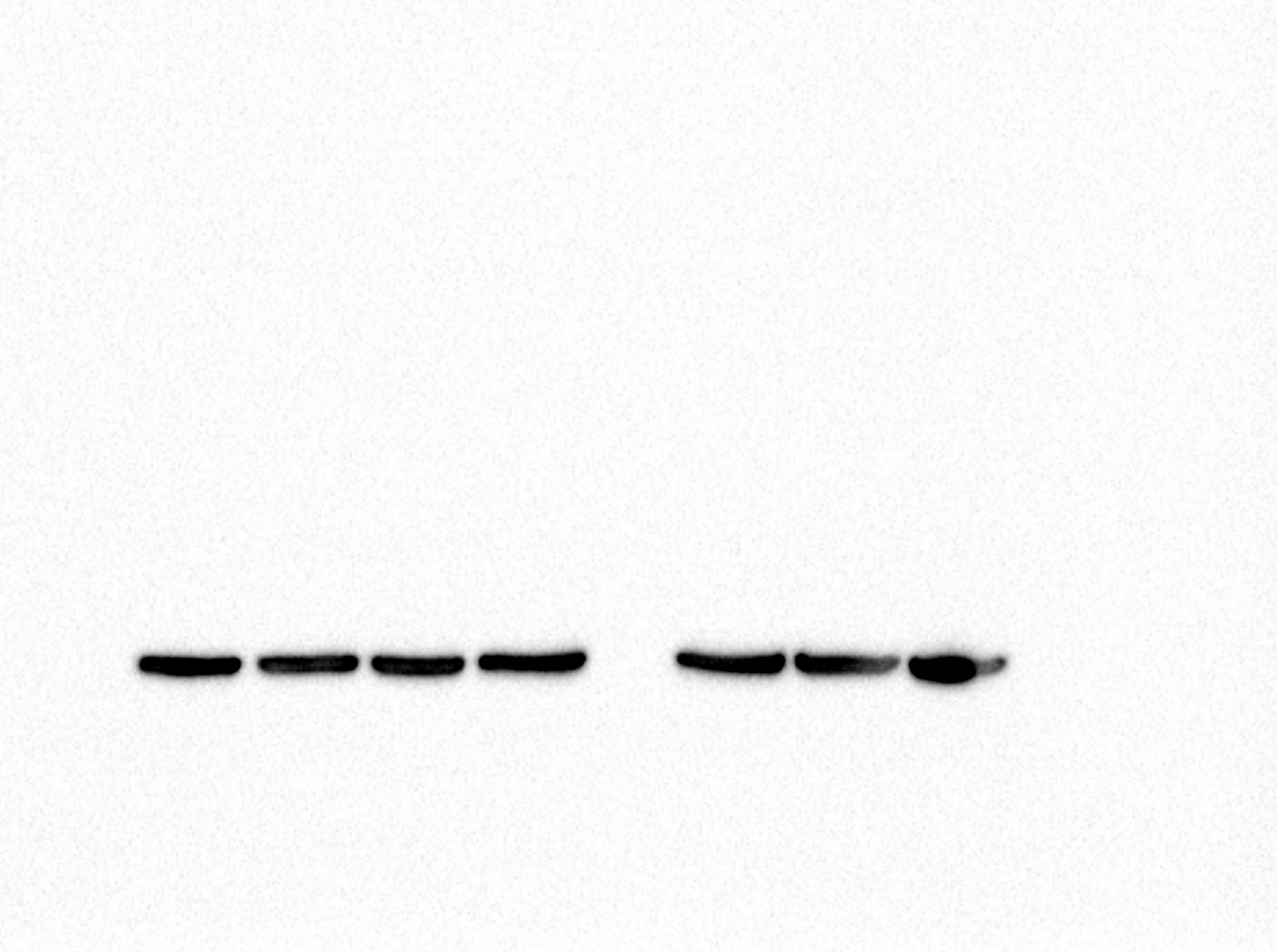

Supplement: Supplementary file 1 [file ijms-21-05939-s001.zip › Supplementary Files/Original images of western blots/Fig 1D-╬▓-actin.tif]

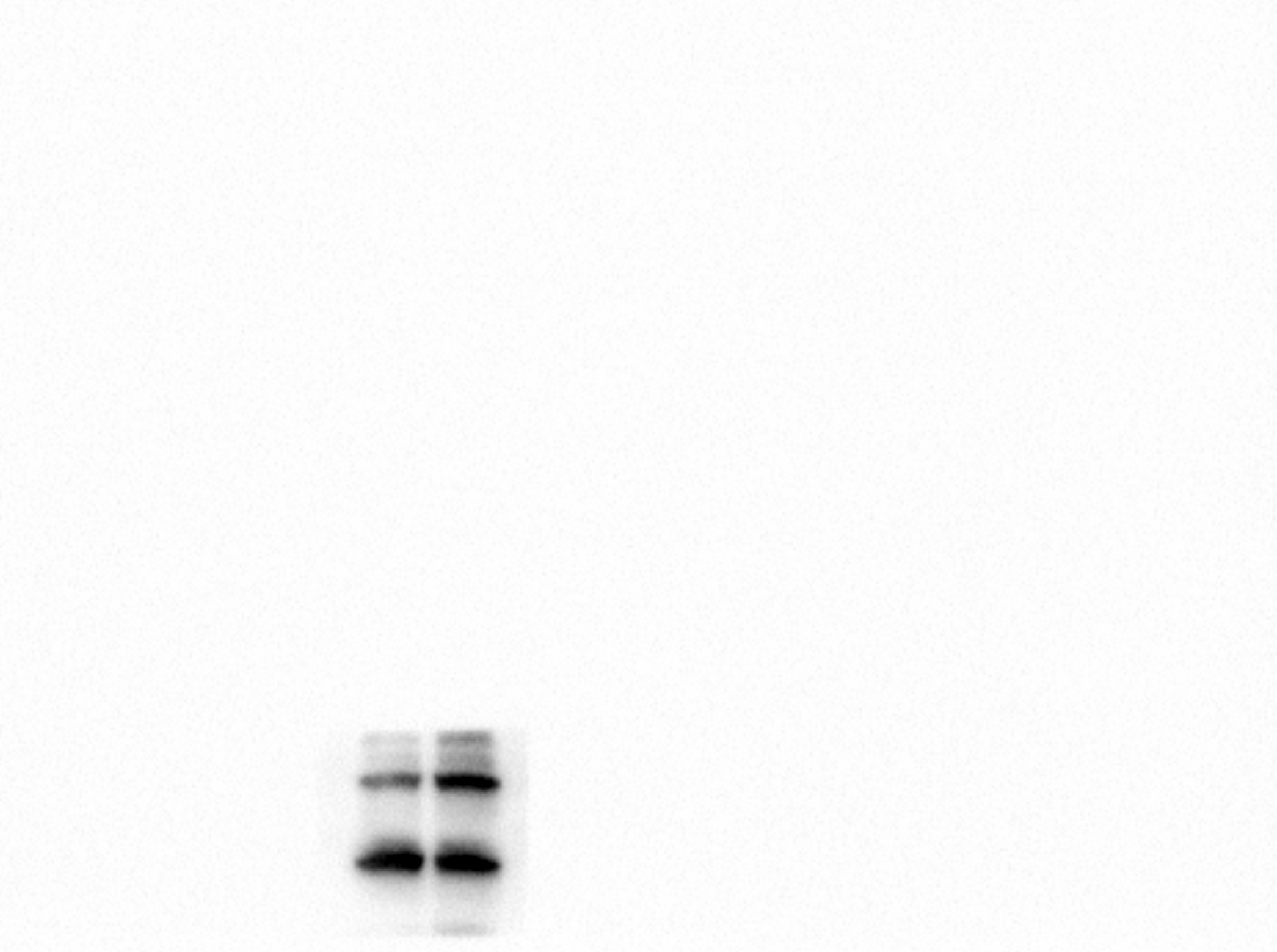

Supplement: Supplementary file 1 [file ijms-21-05939-s001.zip › Supplementary Files/Original images of western blots/Fig 3D-Nrf2.tif]

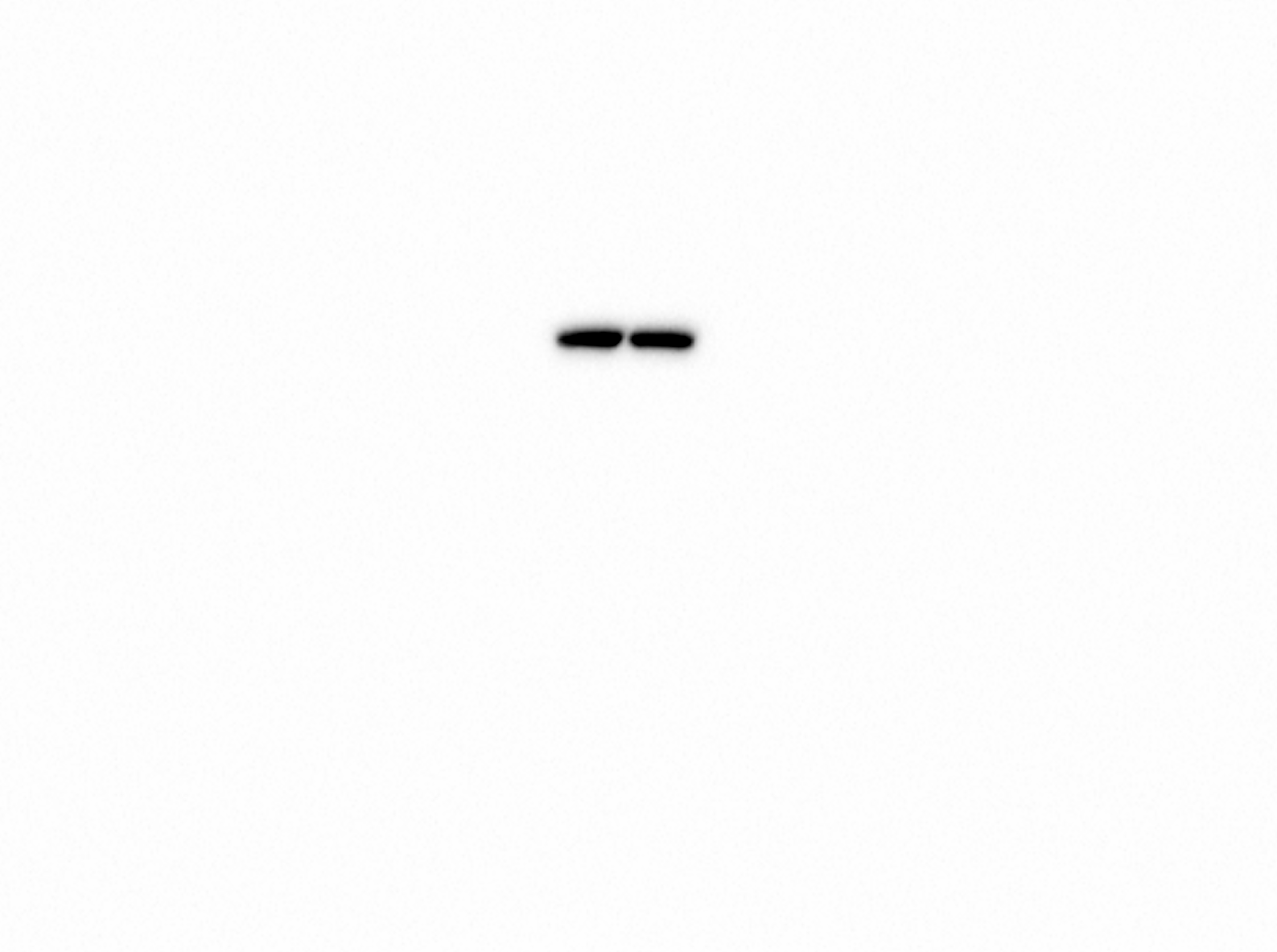

Supplement: Supplementary file 1 [file ijms-21-05939-s001.zip › Supplementary Files/Original images of western blots/Supplementary Figure S2B-╬▓-actin.tif]

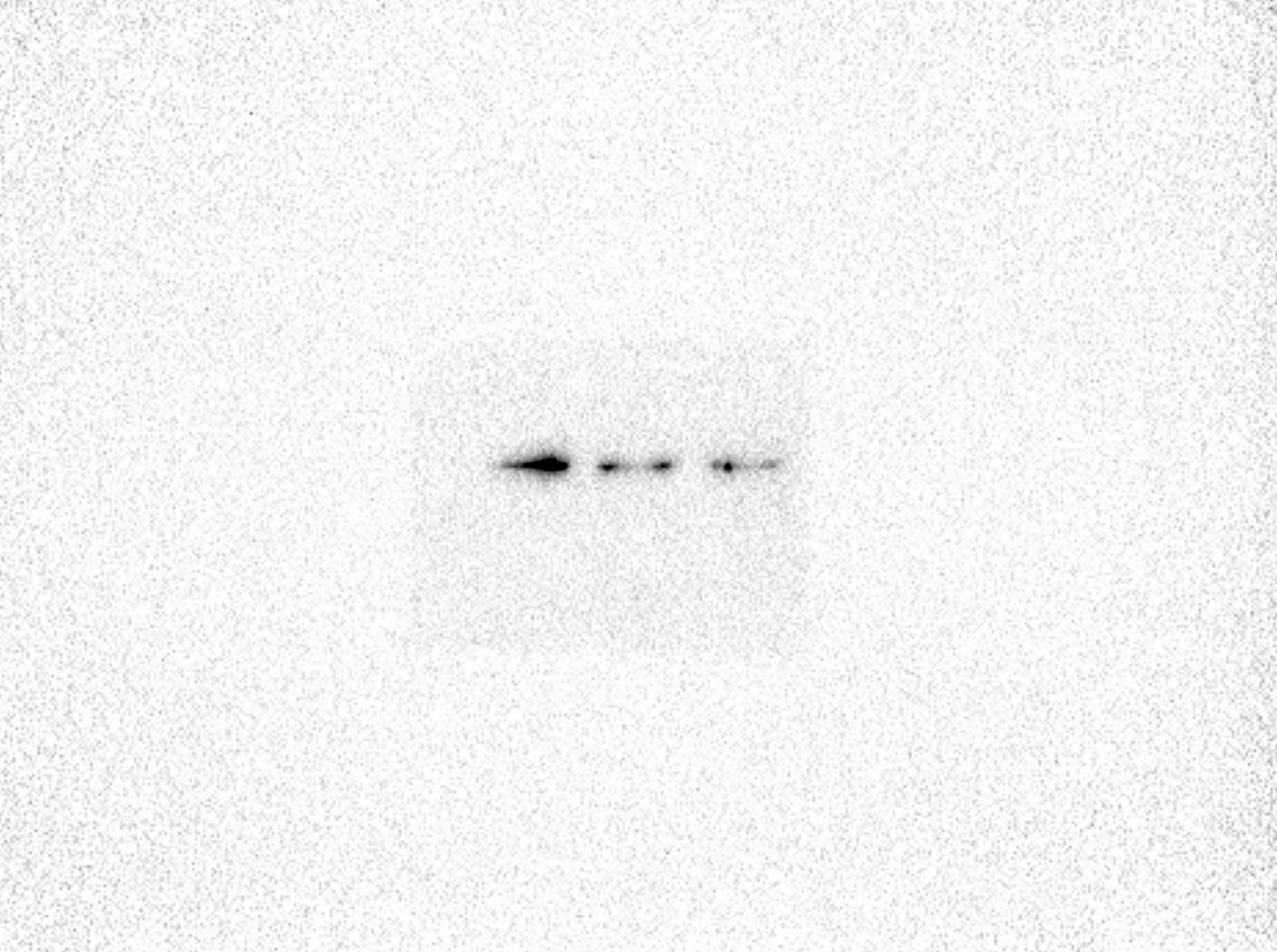

Supplement: Supplementary file 1 [file ijms-21-05939-s001.zip › Supplementary Files/Original images of western blots/Supplementary Figure S1E-CA9.tif]

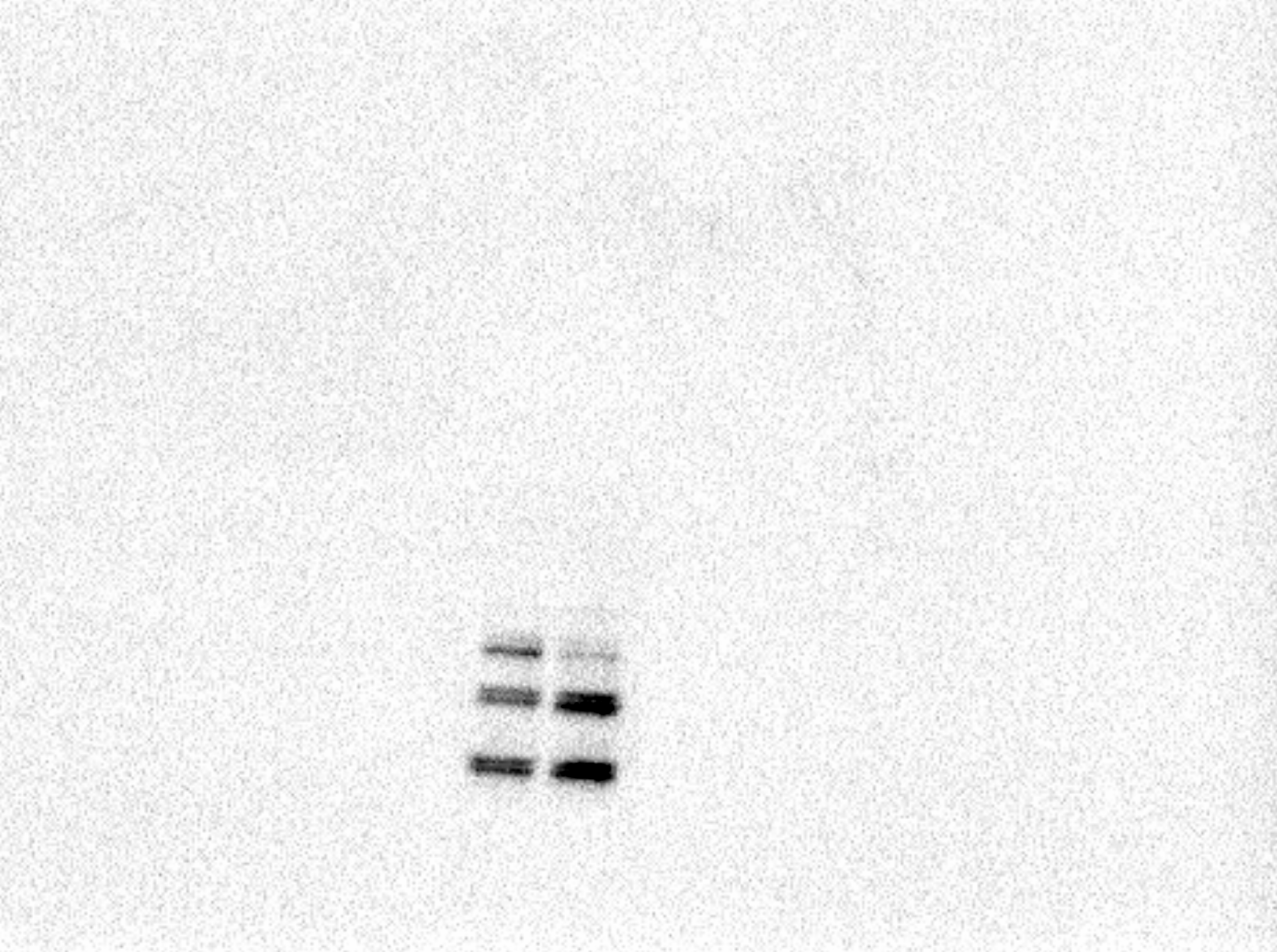

Supplement: Supplementary file 1 [file ijms-21-05939-s001.zip › Supplementary Files/Original images of western blots/Supplementary Figure S2B-NRF2.tif]

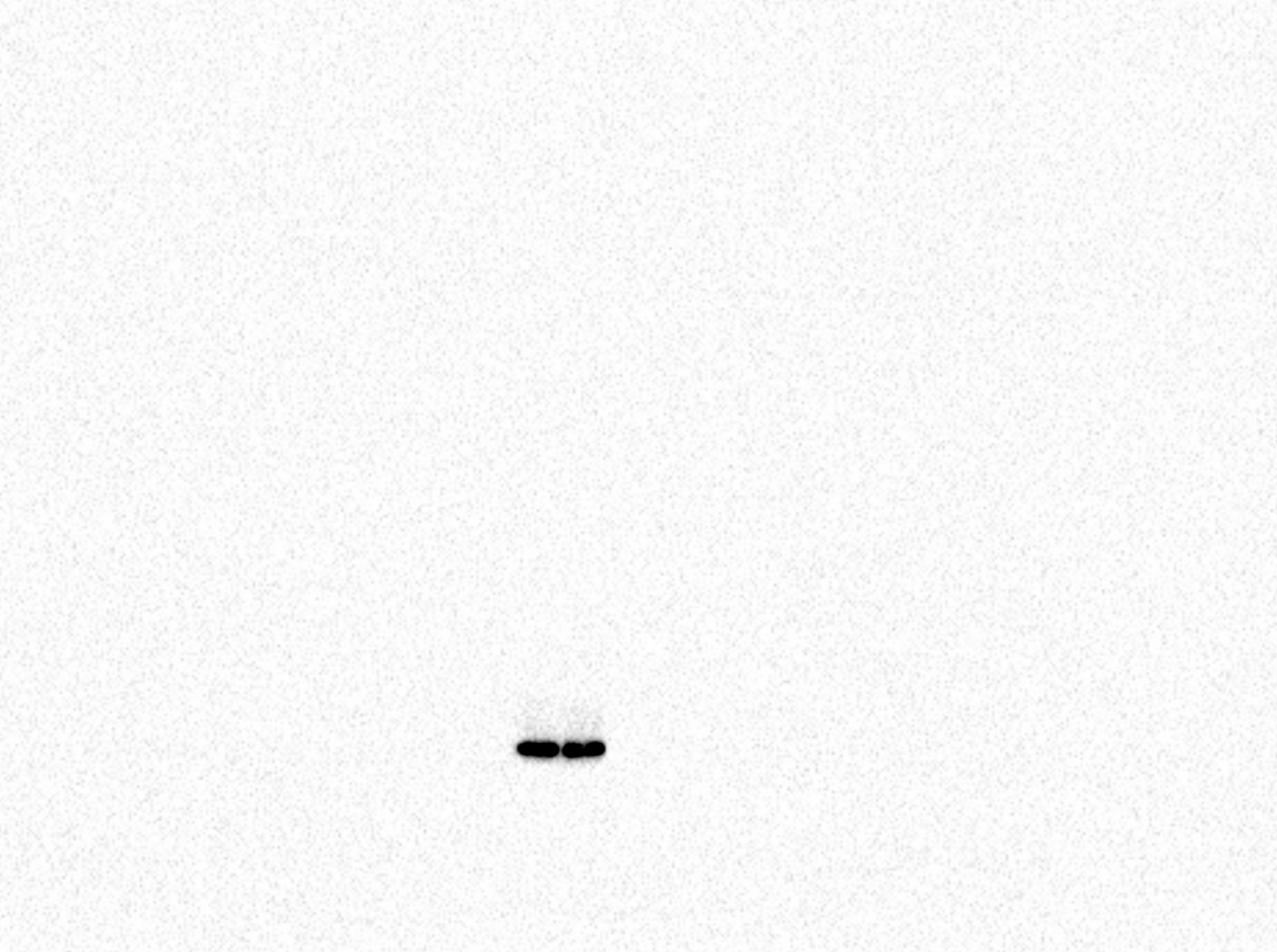

Supplement: Supplementary file 1 [file ijms-21-05939-s001.zip › Supplementary Files/Original images of western blots/Fig 3D-╬▓-actin.tif]

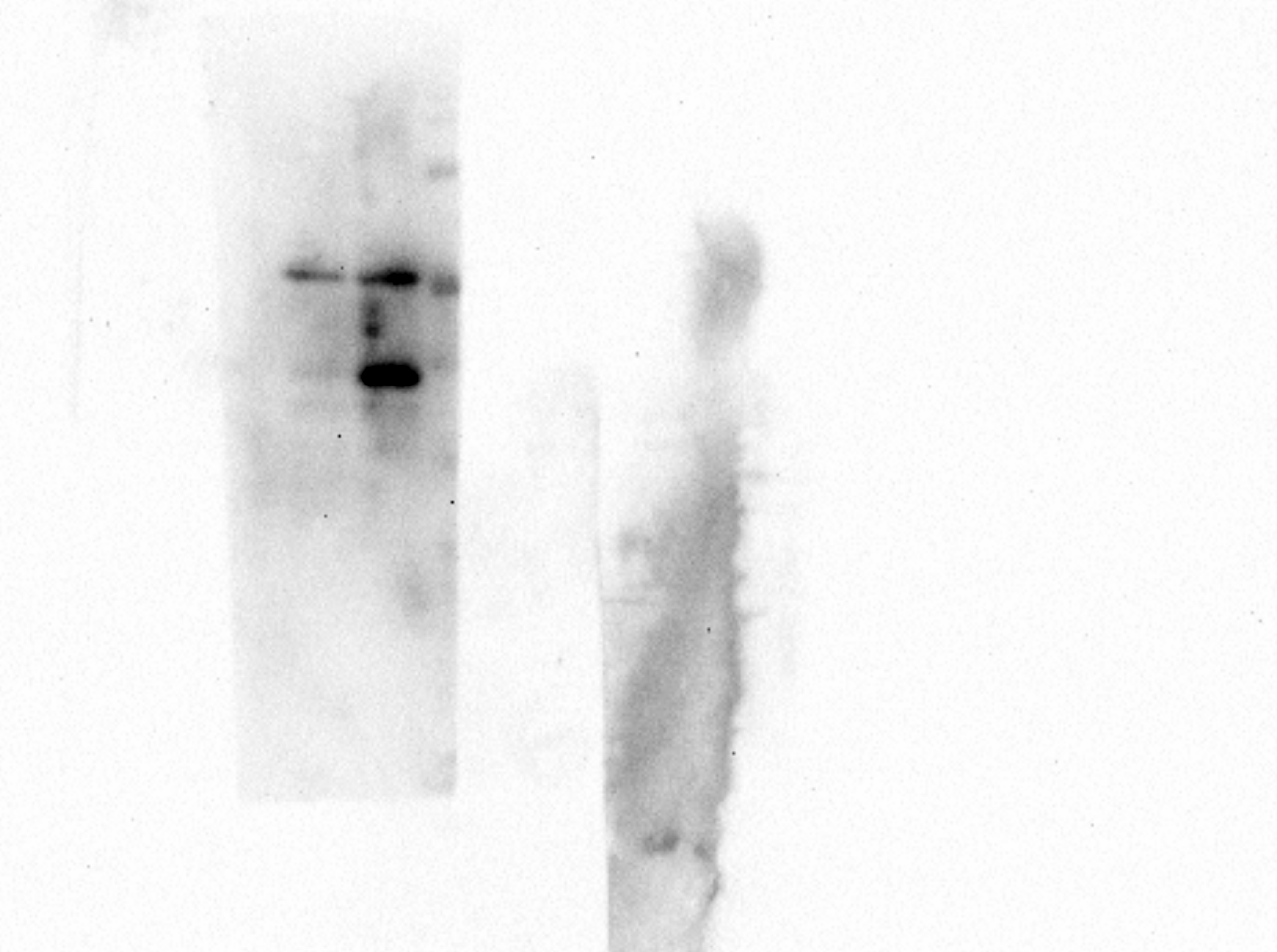

Supplement: Supplementary file 1 [file ijms-21-05939-s001.zip › Supplementary Files/Original images of western blots/Supplementary Figure S4A-ARG2.tif]

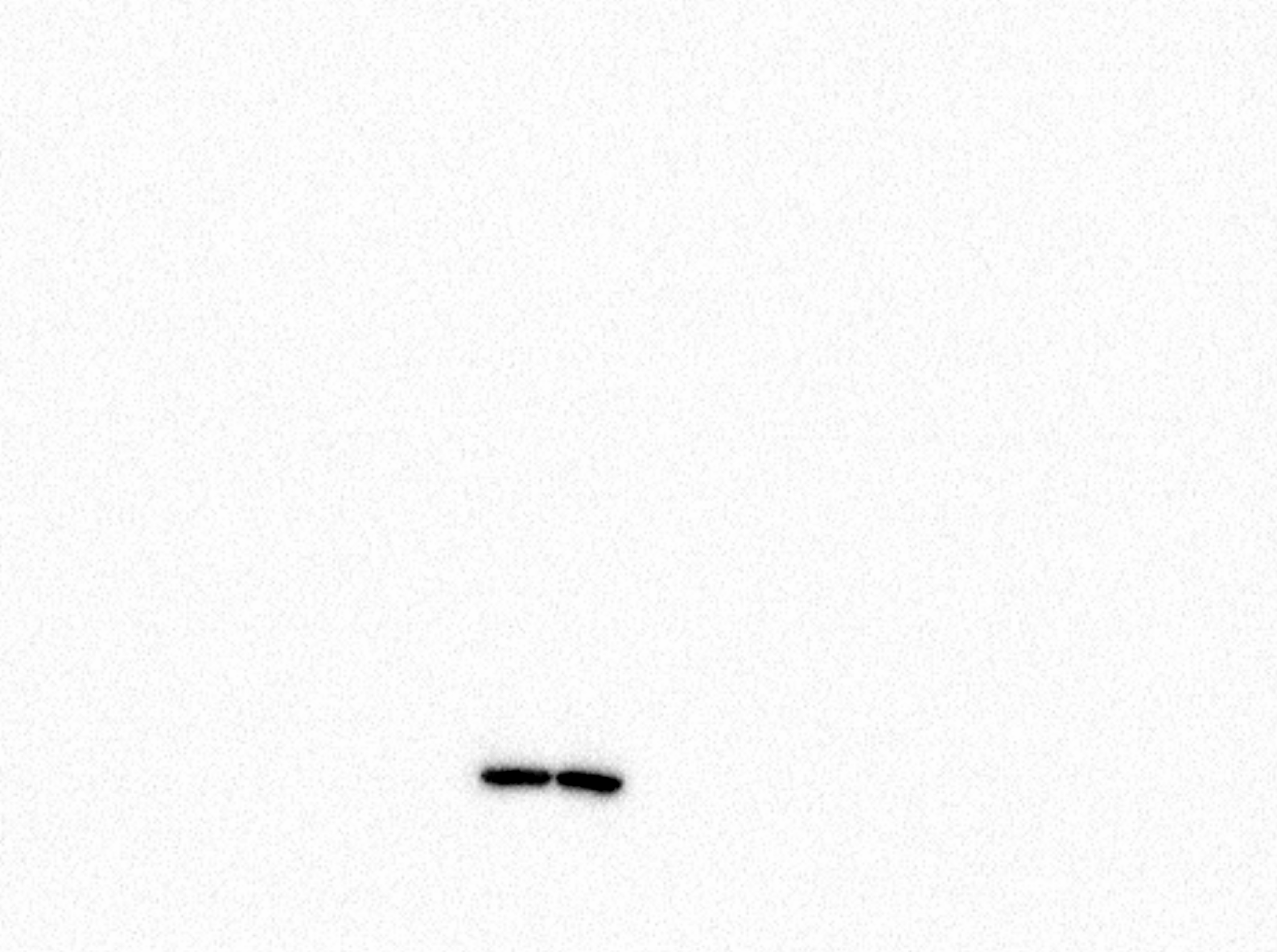

Supplement: Supplementary file 1 [file ijms-21-05939-s001.zip › Supplementary Files/Original images of western blots/Fig 5D-╬▓-actin.tif]

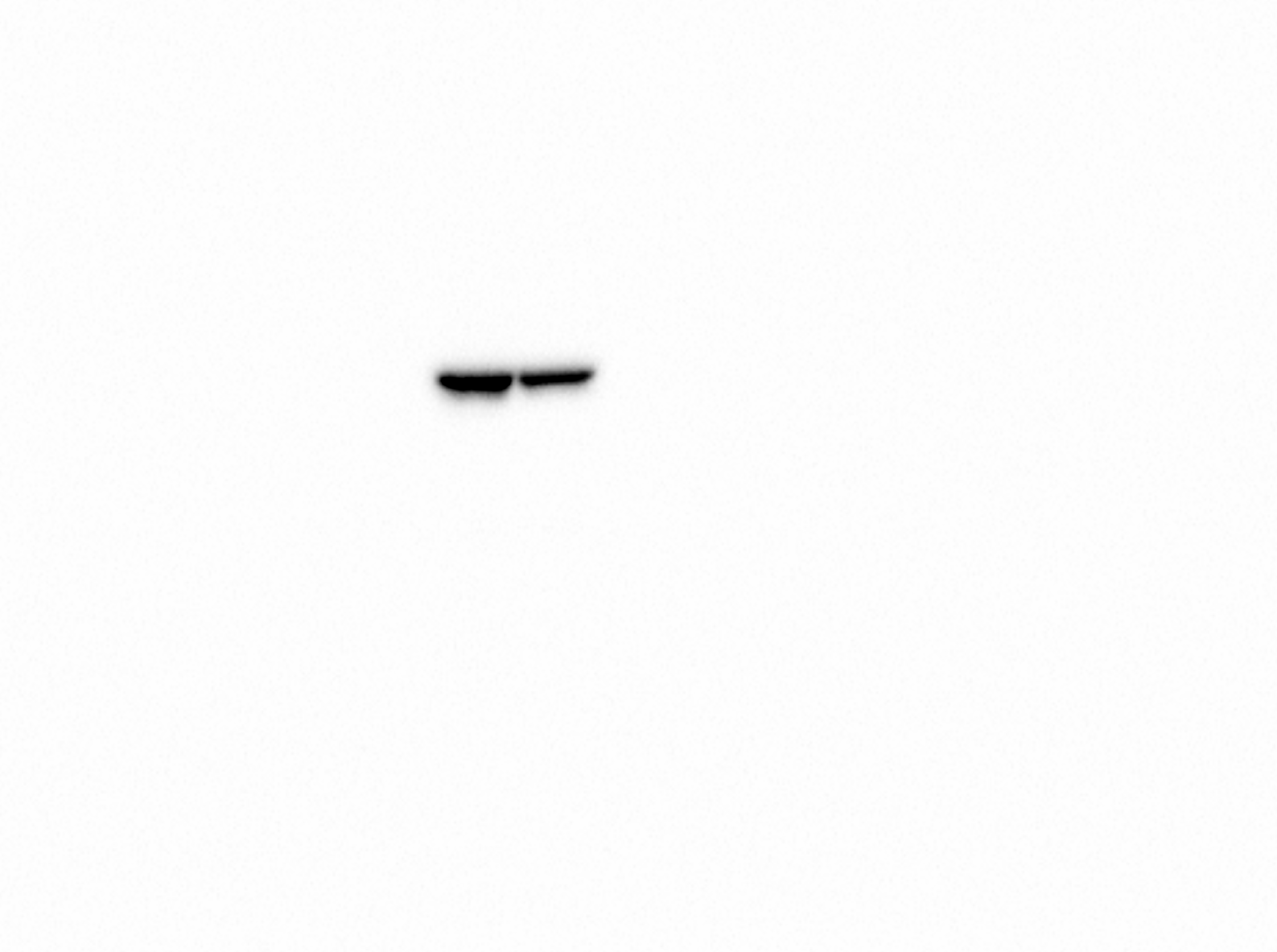

Supplement: Supplementary file 1 [file ijms-21-05939-s001.zip › Supplementary Files/Original images of western blots/Fig 4F-ASL.tif]

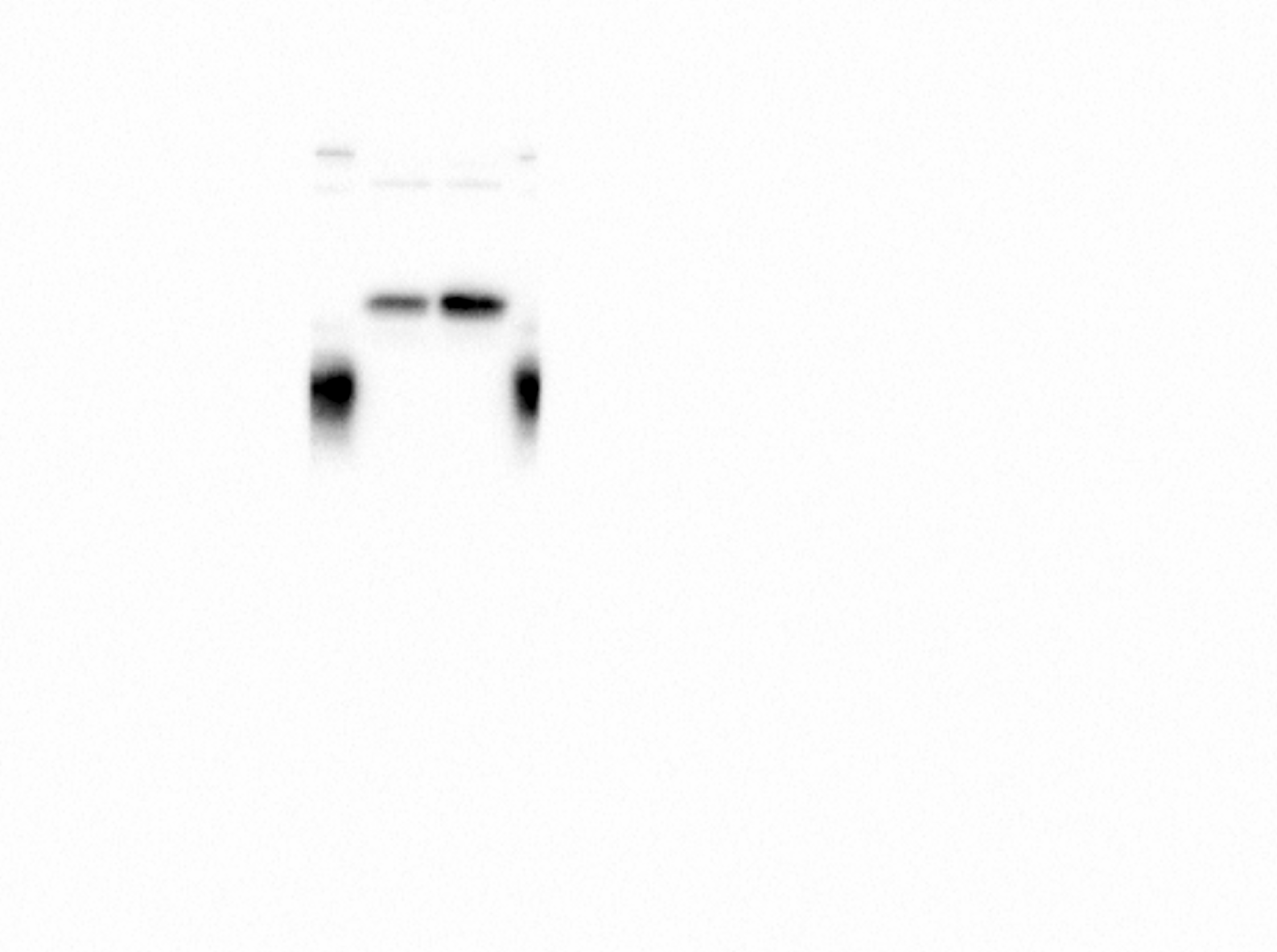

Supplement: Supplementary file 1 [file ijms-21-05939-s001.zip › Supplementary Files/Original images of western blots/Supplementary Figure S2B-TFAM.tif]

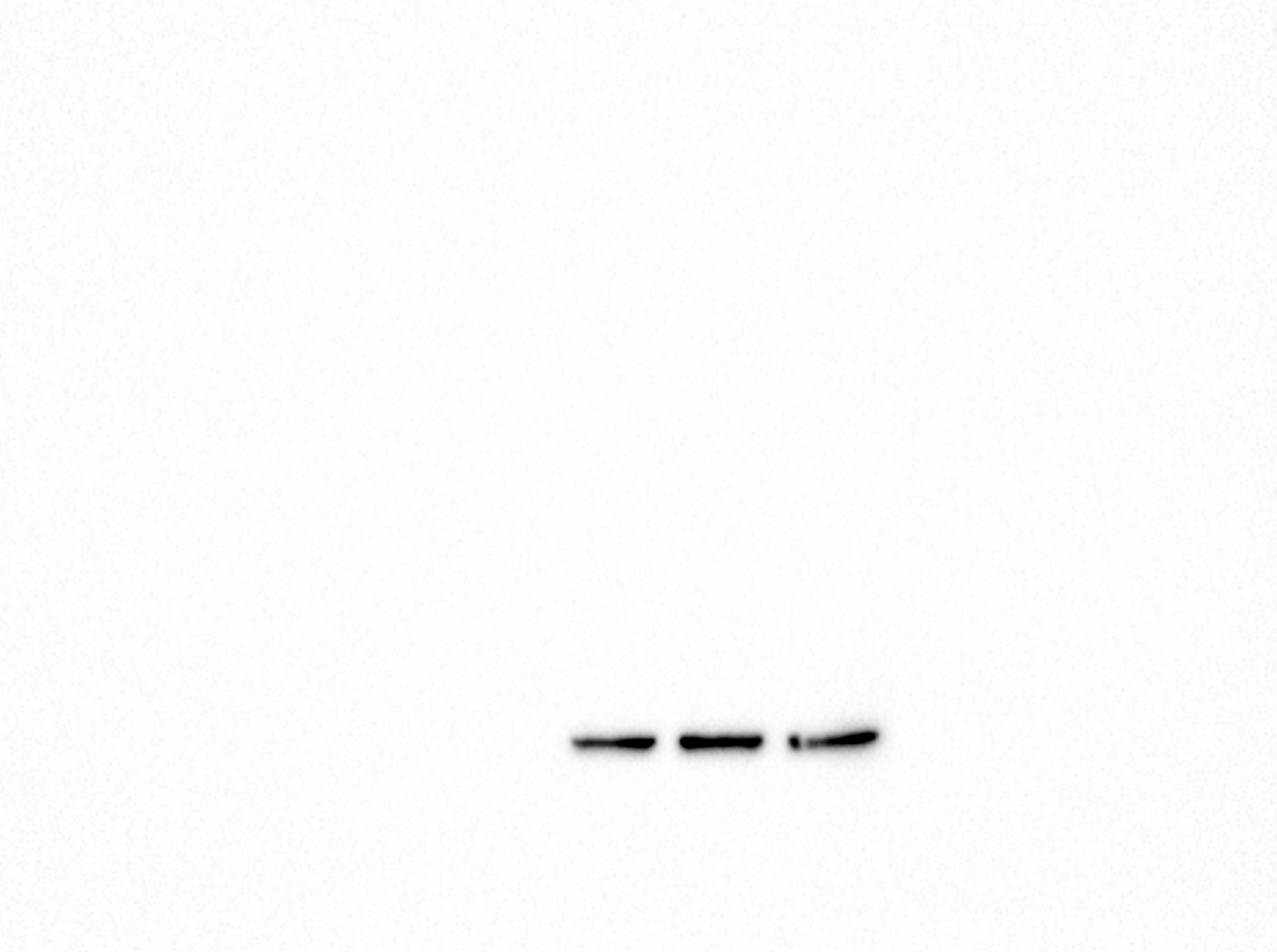

Supplement: Supplementary file 1 [file ijms-21-05939-s001.zip › Supplementary Files/Original images of western blots/Supplementary Figure S1E-╬▓-actin.tif]

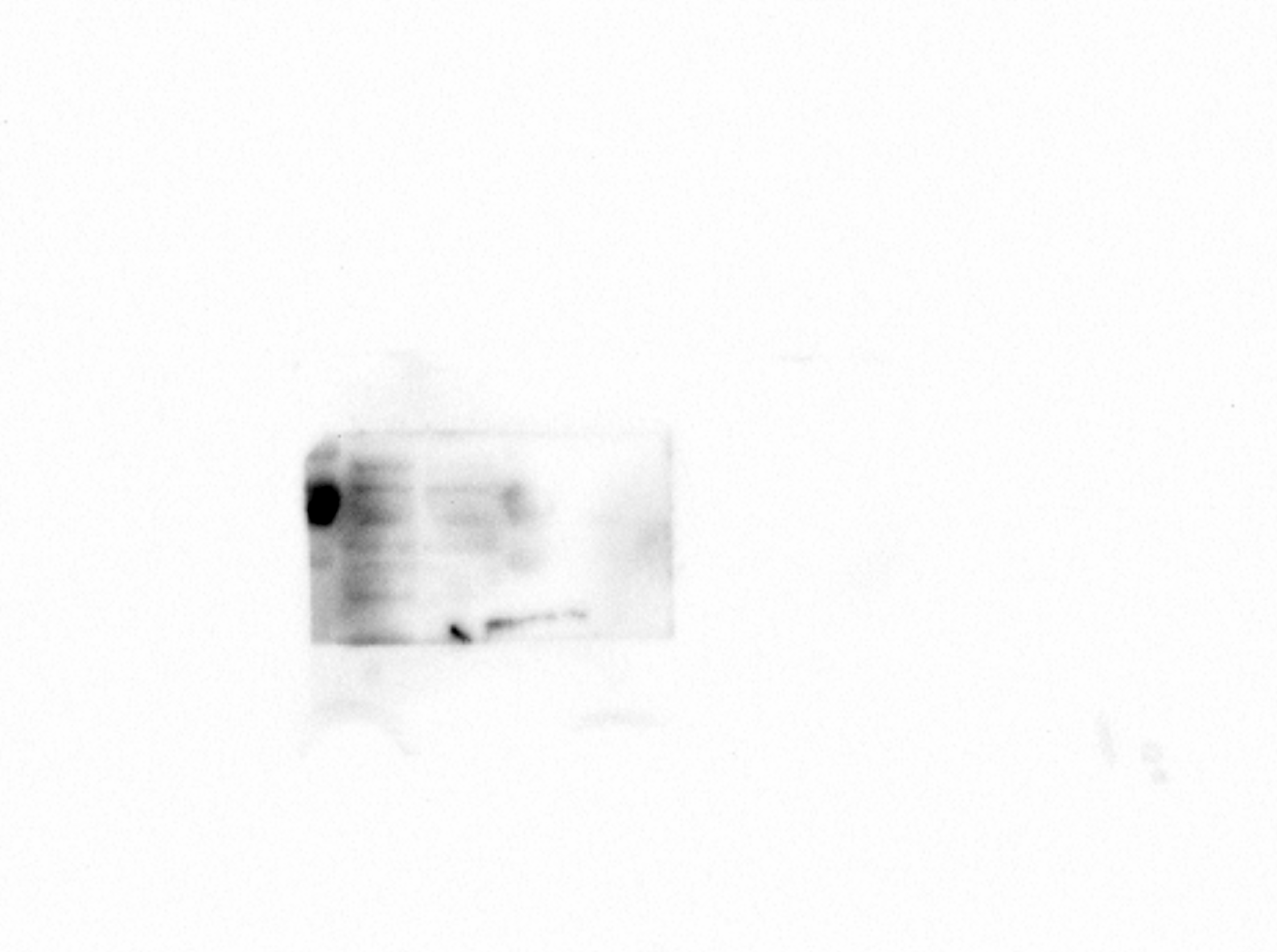

Supplement: Supplementary file 1 [file ijms-21-05939-s001.zip › Supplementary Files/Original images of western blots/Fig 5D-p-p70S6K-T389.tif]

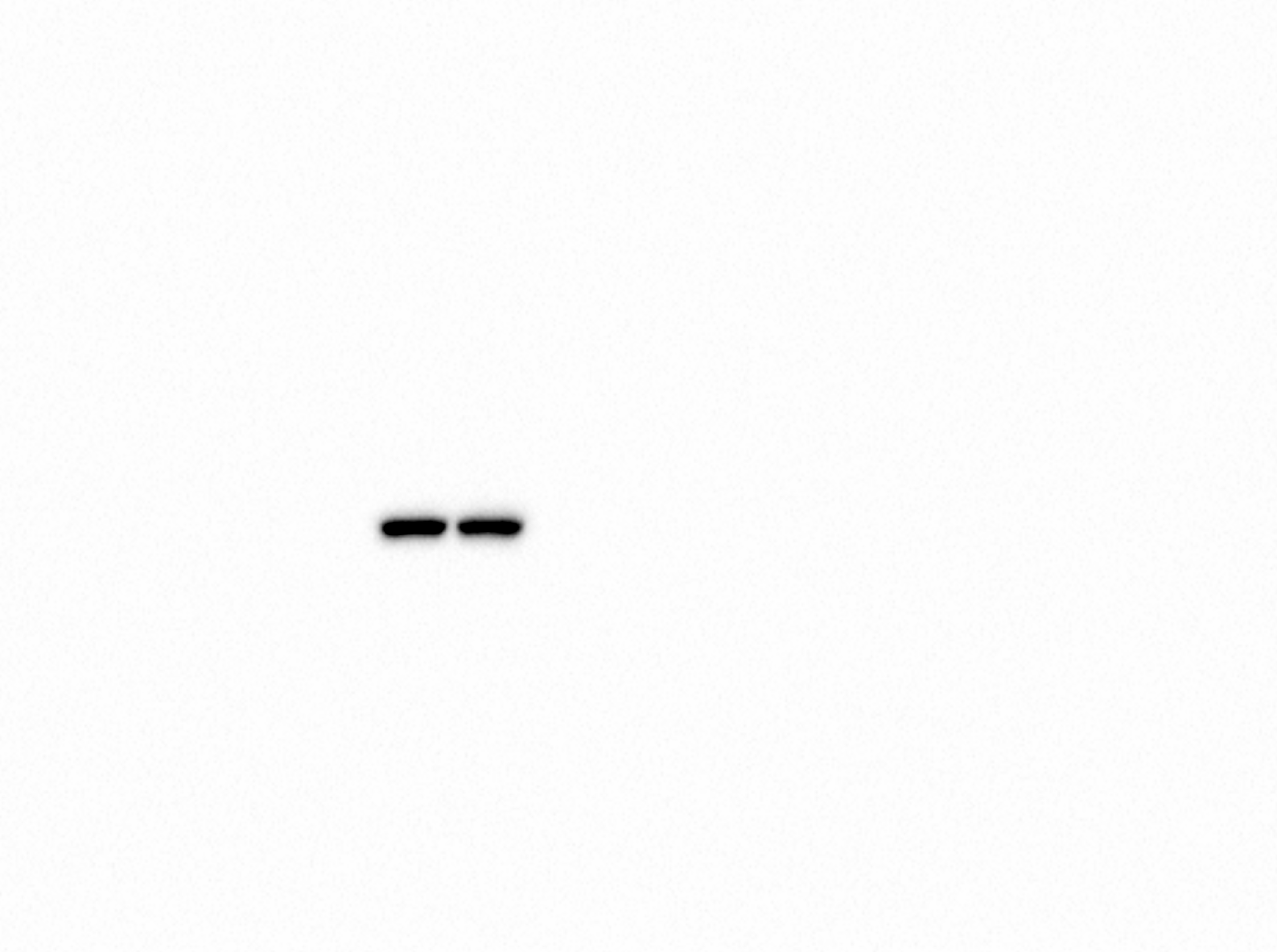

Supplement: Supplementary file 1 [file ijms-21-05939-s001.zip › Supplementary Files/Original images of western blots/Supplementary Figure S4A-╬▓-actin.tif]

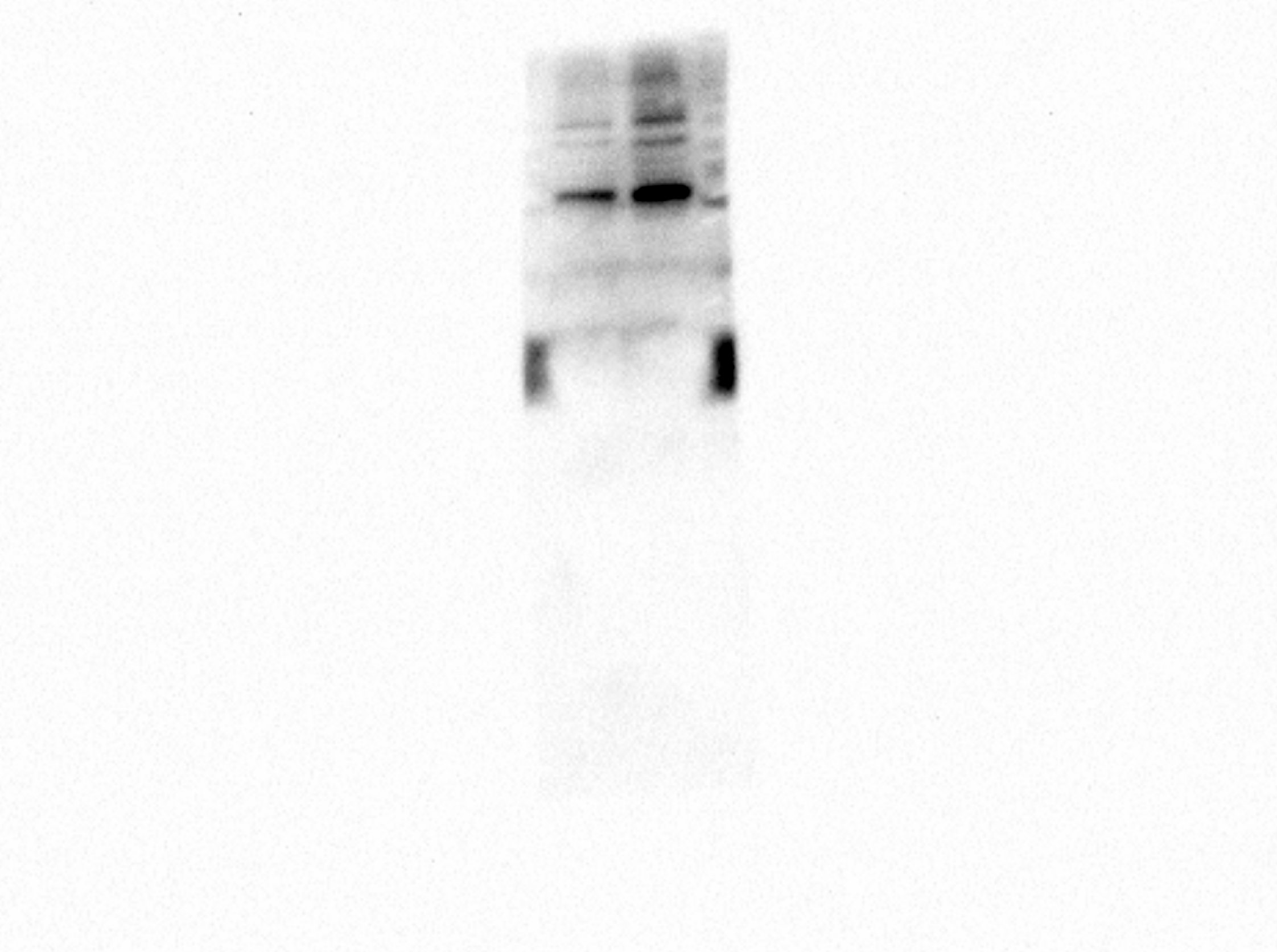

Supplement: Supplementary file 1 [file ijms-21-05939-s001.zip › Supplementary Files/Original images of western blots/Supplementary Figure S2B-ATPAF1.tif]

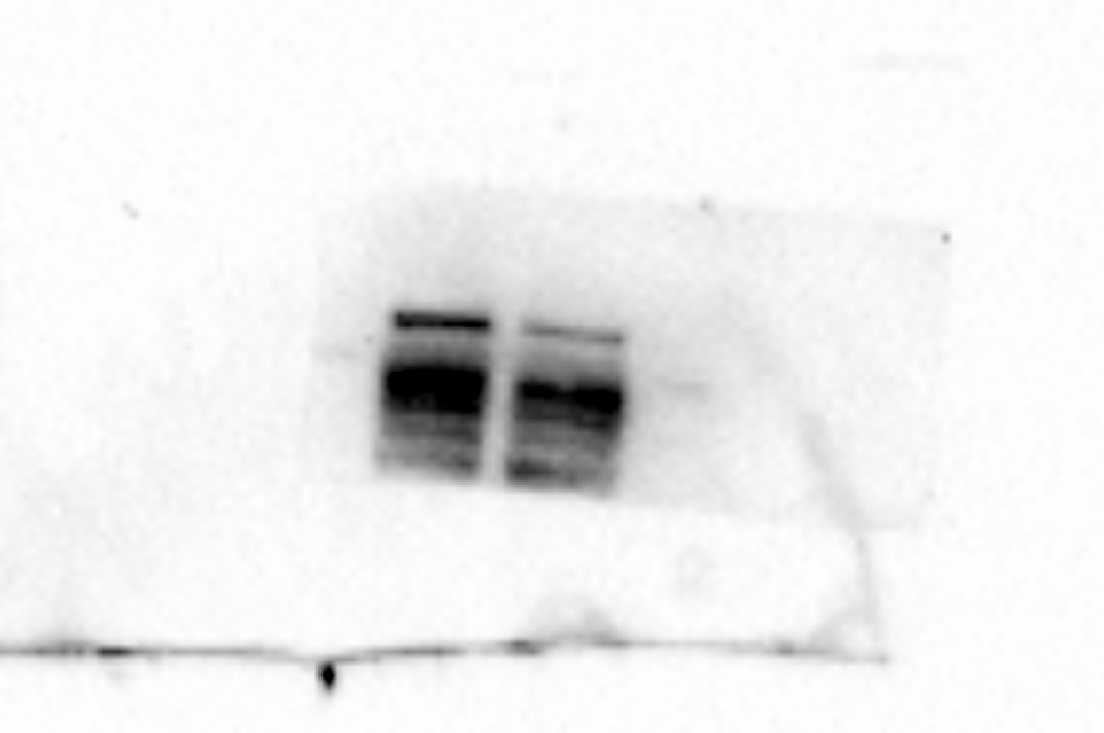

Supplement: Supplementary file 1 [file ijms-21-05939-s001.zip › Supplementary Files/Original images of western blots/Fig 5D-p-mTOR-S2448.tif]

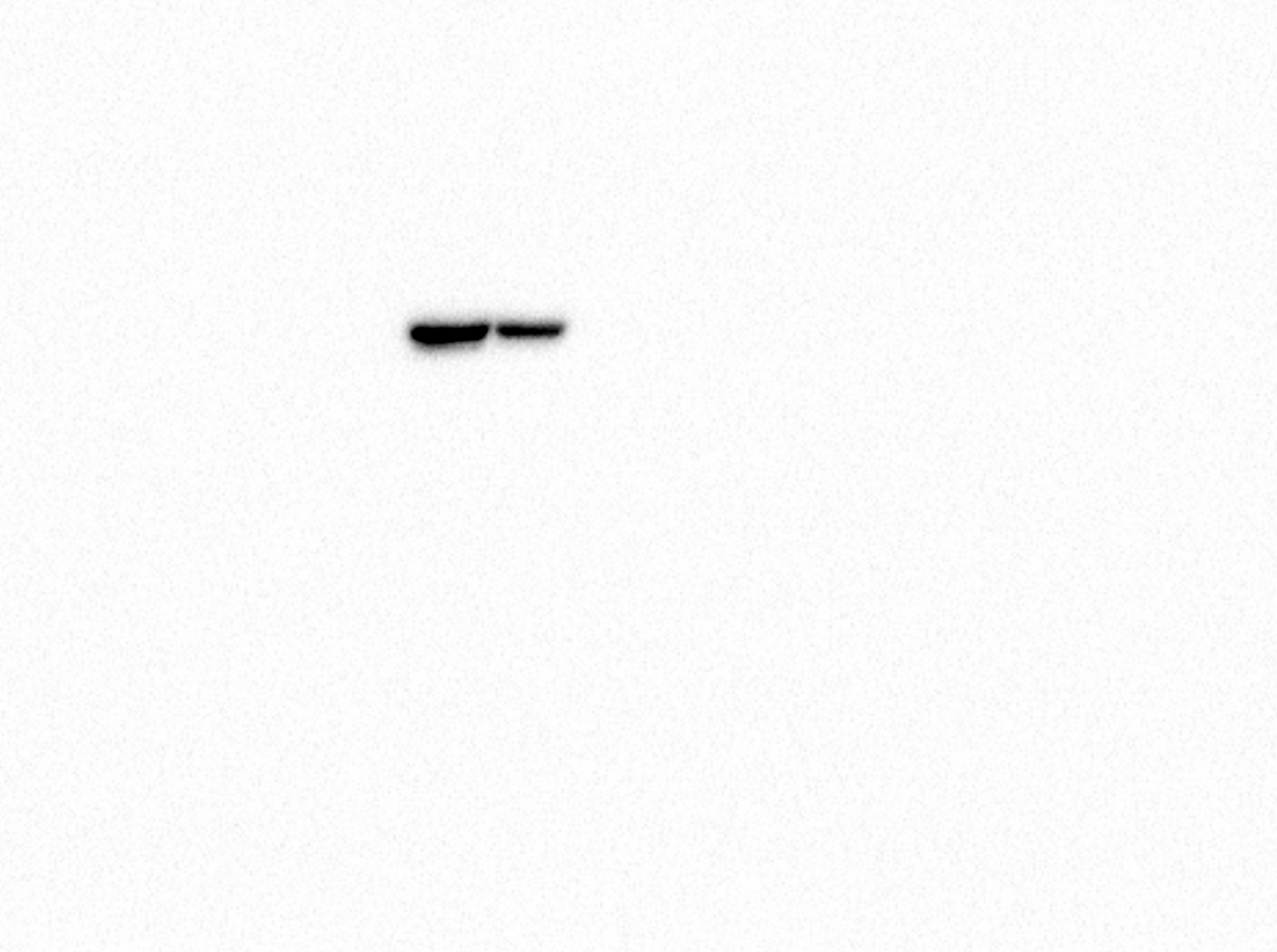

Supplement: Supplementary file 1 [file ijms-21-05939-s001.zip › Supplementary Files/Original images of western blots/Supplementary Figure S4A-ASL.tif]

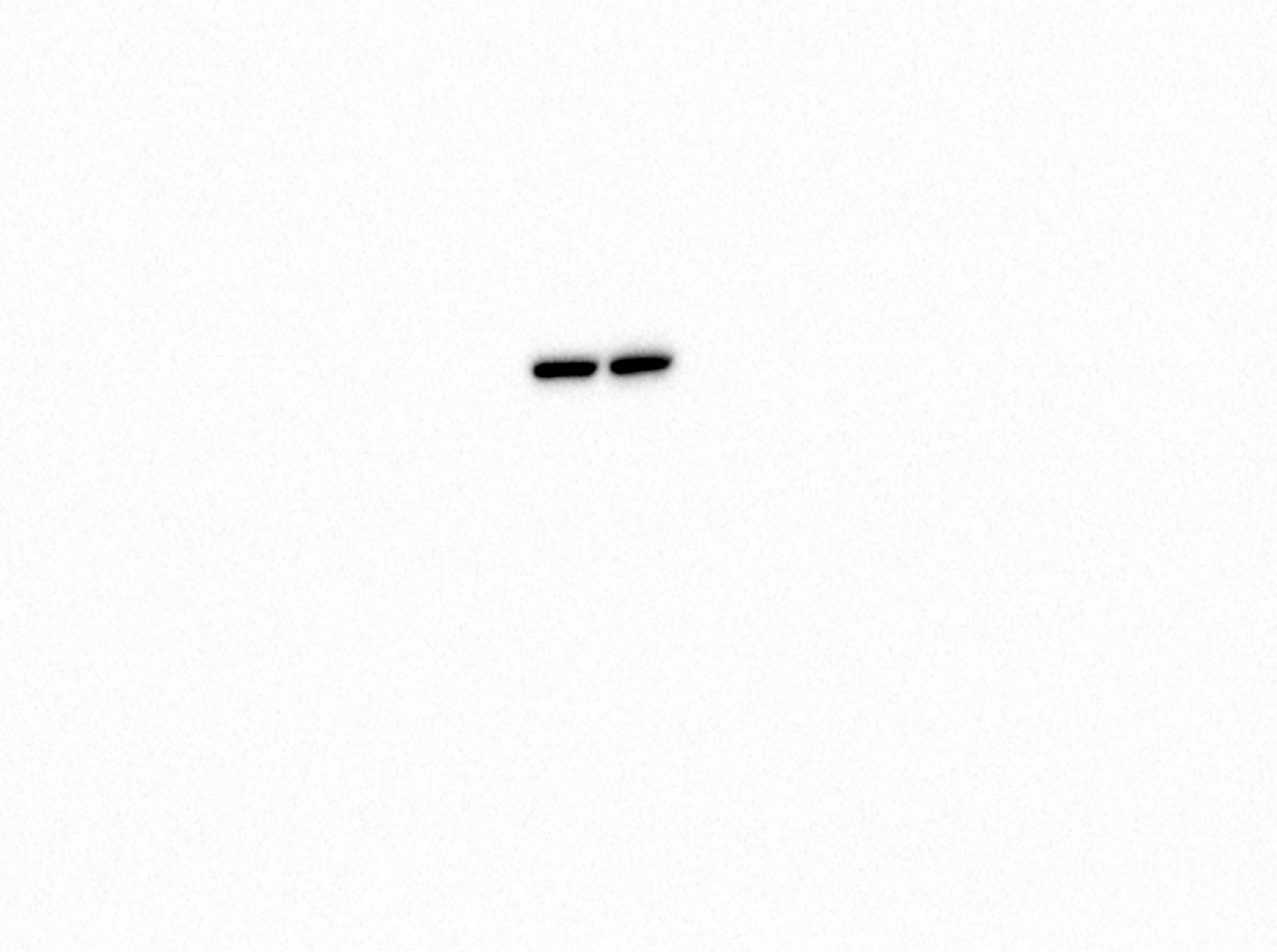

Supplement: Supplementary file 1 [file ijms-21-05939-s001.zip › Supplementary Files/Original images of western blots/Fig 4F-╬▓-actin-2.tif]

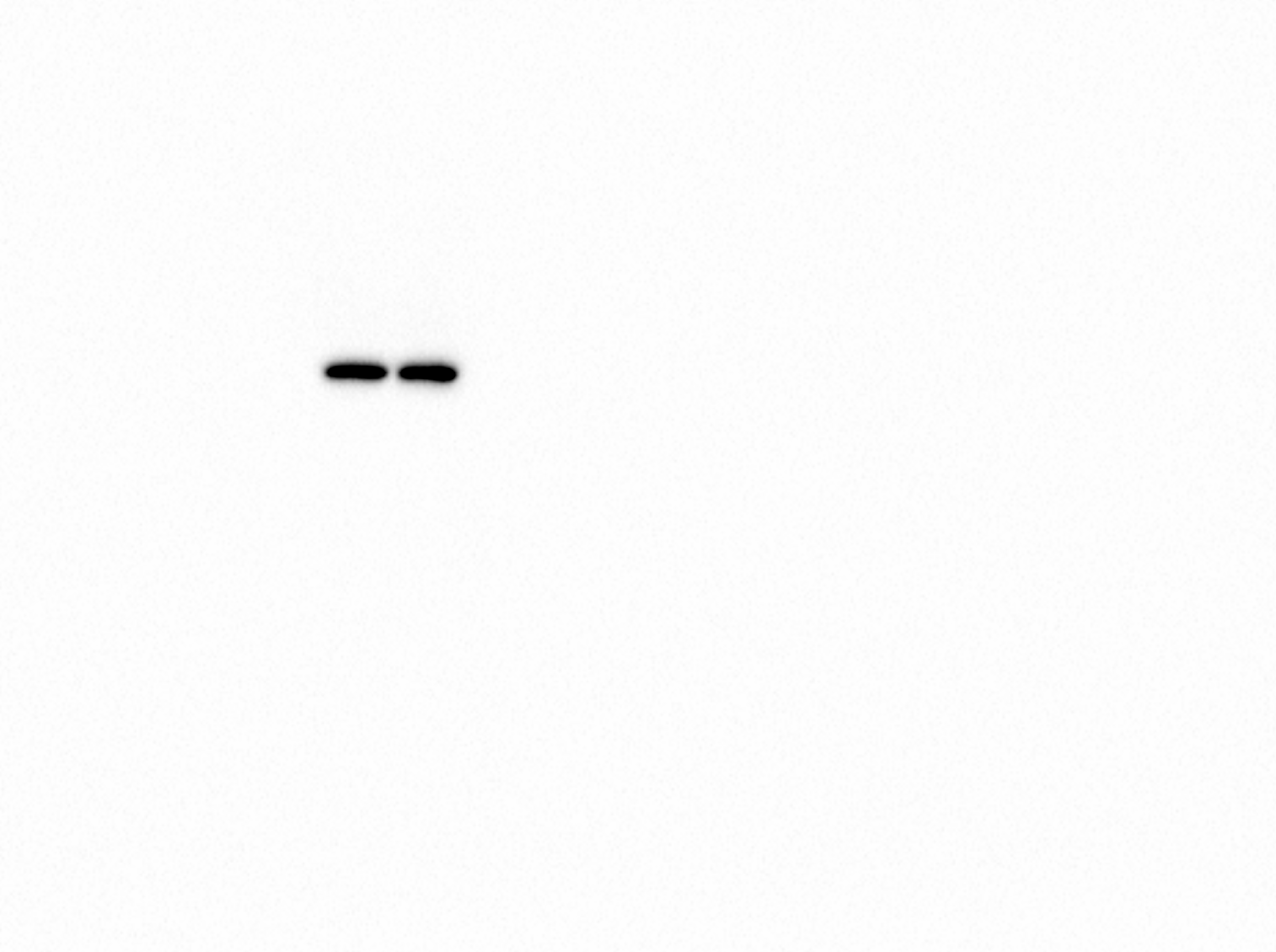

Supplement: Supplementary file 1 [file ijms-21-05939-s001.zip › Supplementary Files/Original images of western blots/Supplementary Figure S1C-╬▓-actin.tif]
